# The erosion of biodiversity and biomass in the Atlantic Forest biodiversity hotspot

**DOI:** 10.1038/s41467-020-20217-w

**Published:** 2020-12-11

**Authors:** Renato A. F. de Lima, Alexandre A. Oliveira, Gregory R. Pitta, André L. de Gasper, Alexander C. Vibrans, Jérôme Chave, Hans ter Steege, Paulo I. Prado

**Affiliations:** 1 Departamento de Ecologia, Instituto de Biociências, Universidade de São Paulo, Rua do Matão, trav. 14, 321, 05508-090 São Paulo, Brazil; 2 Tropical Botany, Naturalis Biodiversity Center, Darwinweg 2, 2333 CR Leiden, The Netherlands; 3 Departamento de Ciências Naturais, Universidade Regional de Blumenau, Rua Antônio da Veiga, 140, 89030-903 Blumenau, Brazil; 4 Departamento de Engenharia Florestal, Universidade Regional de Blumenau, Rua São Paulo, 3250, 89030-000 Blumenau, Brazil; 5 Laboratoire Evolution et Diversité Biologique, UMR 5174 CNRS, Université Paul Sabatier, IRD. 118, route de Narbonne, 31062 Toulouse, France; 6 Systems Ecology, Vrije Universiteit Amsterdam, De Boelelaan 1087, Amsterdam, 1081 HV Netherlands

**Keywords:** Biodiversity, Community ecology, Forest ecology

## Abstract

Tropical forests are being deforested worldwide, and the remaining fragments are suffering from biomass and biodiversity erosion. Quantifying this erosion is challenging because ground data on tropical biodiversity and biomass are often sparse. Here, we use an unprecedented dataset of 1819 field surveys covering the entire Atlantic Forest biodiversity hotspot. We show that 83−85% of the surveys presented losses in forest biomass and tree species richness, functional traits, and conservation value. On average, forest fragments have 25−32% less biomass, 23−31% fewer species, and 33, 36, and 42% fewer individuals of late-successional, large-seeded, and endemic species, respectively. Biodiversity and biomass erosion are lower inside strictly protected conservation units, particularly in large ones. We estimate that biomass erosion across the Atlantic Forest remnants is equivalent to the loss of 55−70 thousand km^2^ of forests or US$2.3−2.6 billion in carbon credits. These figures have direct implications on mechanisms of climate change mitigation.

## Abstract (web summary)

Quantifying forest degradation and biodiversity losses is necessary to inform conservation and restoration policies. Here the authors analyze a large dataset for the Atlantic Forest in South America to quantify losses in forest biomass and tree species richness, functional traits, and conservation value.

## Introduction

Tropical forests are major stocks of biodiversity and carbon; and these stocks are declining worldwide. Half of their original cover has already vanished and current deforestation rates are about 1% per year^1^. Human impacts on tropical forests, however, are not restricted to deforestation. Beyond the reduction in habitat availability and connectivity, deforestation triggers a myriad of modifications that can penetrate up to 1.5 km into the remaining fragments^2–4^. In addition, forest fragments are more accessible, increasing their exposure to fire, selective logging, hunting, and biological invasions. These human-induced impacts on forest fragments (i.e. forest degradation) impose a long-lasting burden on forest biodiversity and biomass stocks^5–12^ that can be as severe as deforestation^13,14^. Protected areas can mitigate the erosion of biodiversity and biomass^15–17^, but their effectiveness is contingent on the type of management and level of anthropogenic pressure surrounding the protected areas^15–17^.

Forest degradation can be assessed by high-resolution remote sensing (e.g. LiDAR^18^), but the coverage of this approach is limited and the impact on biodiversity cannot be measured. This is why large-scale quantifications of the impacts of forest degradation are mostly available for biomass^10–12^. Therefore, field surveys remain essential to quantify the erosion of both biodiversity and biomass^2–4,7,8,10,19^. The simultaneous evaluation of forest degradation on tropical biodiversity and biomass at large-scales provides crucial knowledge for the conservation and climate change agenda^13,17,20^ and to refine regional assessments of biodiversity and ecosystem services (e.g. Intergovernmental Science-Policy Platform on Biodiversity and Ecosystem Services—IPBES).

Here, we aim at quantifying the impacts of forest degradation on a major biodiversity hotspot located in eastern South-America, the Atlantic Forest (Supplementary Fig. 1). Home to 35% of the South American population, the Atlantic Forest is one of the most fragmented tropical/subtropical forests in the world^12,21^, which may well represent the present or future of other tropical forests worldwide^22^. To achieve our goal, we create one of the largest datasets of forest surveys ever assembled for the tropics and subtropics^23^, both inside and outside protected areas. This dataset includes data on forest biomass and tree species richness and/or composition, as well as carefully curated metadata associated with each survey, representing a total of 1819 field surveys, 1.45 million trees, 3124 tree species, and 1238 ha of sampling coverage (Table 1, Supplementary Data 1). The dataset covers the entire range of environmental conditions, landscape contexts, and disturbance histories of the Atlantic Forest (Supplementary Fig. 2, Supplementary Table 1). It also contains information on multiple species properties, including plant functional traits (i.e. wood density, maximum height, seed mass), ecological groups (or successional status, e.g. pioneer) and their conservation value (i.e., threat status and endemism level), which enable to assess human-induced impacts on community composition sensu lato.

**Table 1:** Summary of the Atlantic Forest surveys studied.

Dbh inclusion criteria (cm)	Number of surveys	Total effort (ha)	Number of trees included	Number of tree species
≥3.0–3.2	130	35.9	87,019	907
≥4.8–5.0	1063	703.5	1,059,410	2987
≥10.0	626	498.4	301,933	1291
All criteria	1819	1237.8	1,448,362	3124

Description of the tree community surveys used to estimate human-related impacts on forest fragments, separated by diameter at breast height (dbh) inclusion criteria. Number of species per dbh inclusion criterion refers only to the tree community surveys included in the analyses of multiple species properties (*n* = 1213).

Using this dataset, we quantify forest degradation impacts on the above-ground biomass stocks, tree species richness, and multiple species properties. More specifically, we assess the extent and magnitude of those impacts by asking: (i) how pervasive negative impacts are across this biodiversity hotspot? (ii) how much these biodiversity and biomass losses represent compared to low-disturbance Atlantic Forests? And (iii) can protected areas and human presence explain those losses? Next, we explore the implications of our results to the conservation of what’s left of this biodiversity hotspot by (iv) projecting forest degradation impacts to the remaining Atlantic Forest area to estimate the total amount of carbon lost. We also (v) explore the costs and benefit of two contrasting scenarios of restoration of Atlantic Forest fragments: one focusing only on the reduction of within-fragment disturbance level (i.e. ‘fragment restoration’ scenario) and another focusing on the increase of fragment size and landscape connectivity (i.e. ‘landscape restoration’ scenario).

In general, we quantify forest degradation impacts on Atlantic Forest biodiversity and biomass as follows. First, the variation in forest biomass, species richness and species properties are described using linear mixed-effects regression models. These models account for the effects of environmental and human-related variables, as well as sampling and biogeographical effects (see Methods and Supplementary Figs. 3−7), which explain 53% of the variation in biomass, 71% in species richness and 26−44% in species properties (Supplementary Fig. 8, Supplementary Table 2 and 3). Next, we use these regression models to generate baseline predictions in the absence of major human impacts, i.e. predictions as if all sites were large, low-disturbance forest patches in landscapes with 100% of forest cover. We validate the precision of these predictions using simulations (Supplementary Table 4). Finally, we calculate an index of loss due to human-induced impacts, defined as the standardized difference between observed values and baseline predictions. Values of the index close to zero indicate little human impact and the more negative the value, the greater the impact. We reveal that human-induced impacts on forest biodiversity and biomass are pervasive across Atlantic Forest remnants, with losses reaching up to 42% of the predicted for a human-free scenario. We estimate that the biomass erosion across this biodiversity hotspot is equivalent to the deforestation of over 50 thousand km^2^, translating into the loss of billions of dollars in carbon credits. Thus, our results support the idea that the conservation of tropical carbon and biodiversity depends not only on halting deforestation or restoring degraded lands, but also on mitigating forest degradation in both protected areas and private lands.

## Results and discussion

### Extent and magnitude of human impacts

**Fig. 1:**
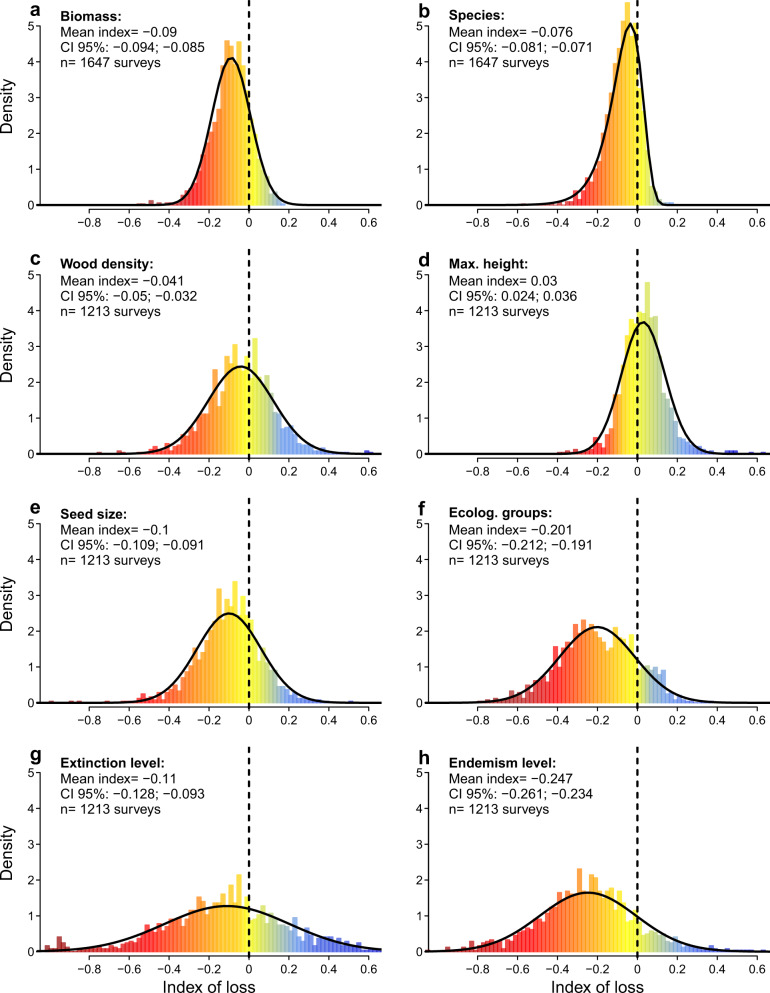
The distribution of the standardized indices of biomass, species richness, and properties loss in the Atlantic Forest hotspot. Frequency distribution (coloured bars) of the index of loss for **a** forest biomass; **b** tree richness; **c** wood density; **d** maximum height; **e** seed mass; **f** ecological groups; **g** extinction threat; and **h** endemism level, with their fits by the Normal or Weibull distributions (solid bold lines), the estimated mean and its 95% confidence interval (CI). Dashed lines separate negative indices (losses due to human-related impacts) from positive ones (gains due to human-related impacts). Bars are highlighted by colours ranging from dark red (high losses) to blue (gains). The standardized index of loss is dimensionless and is highlighted by different colours ranging from dark red (high losses) to blue (gains).

**Fig. 2:**
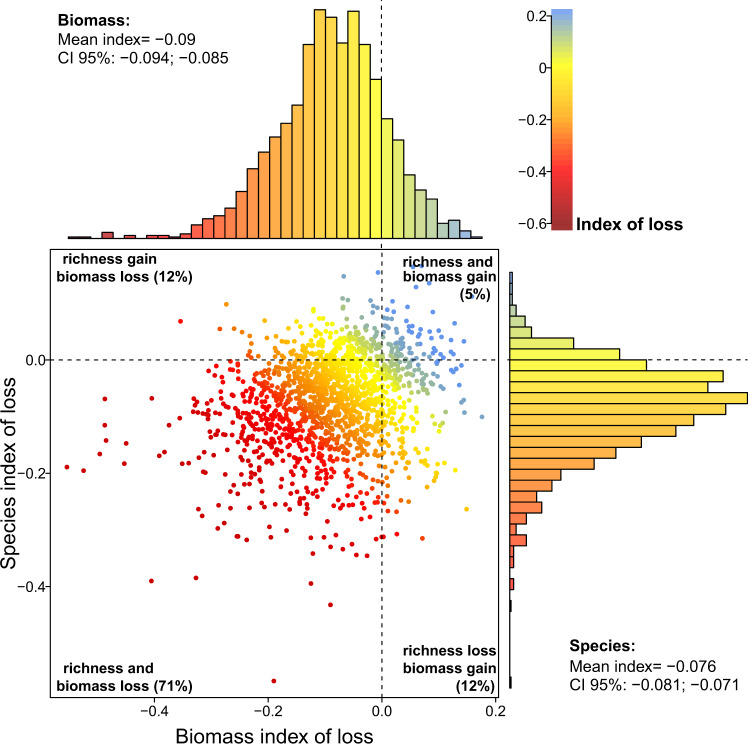
The relationship between losses of species richness and forest biomass due to human-induced impacts. The standardized indices of loss were estimated for each forest survey (points), with biomass on the *x*-axis and species on the *y*-axis. By the margin of each axis, the distribution of the index is presented with the mean and the corresponding 95% confidence interval (CI 95%). Dashed lines separate negative indices (losses) from positive ones (gains). The index of loss is dimensionless and is highlighted by different colours ranging from dark red (high losses) to blue (gains). Pearson’s correlation coefficient between the two indices was 0.22 (*p* < 0.0001, *n* = 1647).

The distribution of the standardized indices of loss was significantly negative for species richness, forest biomass and most of the species properties (Fig. 1), with the majority of the Atlantic Forest surveys (71%) presenting negative indices of loss (i.e. losses) for both forest descriptors (Fig. 2). These losses were correlated with each other (Fig. 2), meaning that fragments that suffer greater losses of biomass also lose more species. In absolute terms, human-induced impacts corresponded to average declines of 23–32% of the richness and biomass relative to low-disturbance Atlantic Forests (Table 2, Supplementary Table 5). Similar estimates (18–57%) have been reported at smaller spatial scales for Neotropical rainforests^8,10,13^ and at global scale^11,14,17^, suggesting that our estimates of richness and biomass erosion are representative of the Atlantic Forest. We did not explicitly model the distances to forest edge (see Supplementary Methods), where biomass erosion can be even greater^2,6–8^. But assuming that researchers tend to avoid edges while establishing plots, our estimates are probably conservative.

**Table 2:** The magnitude of the loss of forest biomass, species richness, and species properties due to human-induced impacts.

Forest descriptor	Dbh cut-off (cm)	Absolute loss (%)
Forest biomass	≥5.0	25.3
	≥10.0	32.0
Tree species richness	≥5.0	30.9
	≥10.0	22.9
Species properties		
Wood density	≥5.0	2.2
Max. adult height	≥5.0	−1.9
Seed mass	≥5.0	35.7
Ecological group	≥5.0	32.6
Extinction level	≥5.0	25.2
Endemism level	≥5.0	42.1

The average proportion of losses were obtained from the averages of the absolute loss predicted (predicted–observed values) for each survey normalized by the reference values of low-disturbance Atlantic Forest fragments (Supplementary Table 5). The average of the proportional absolute loss was weighted by the probability of having a surveyed fragment of the same size in the entire pool of Atlantic Forest fragments, a probability obtained from a log-normal distribution fitted to the size distribution of the ~250,000 forest fragments. This procedure was conducted separately for each biogeographical region of the Atlantic Forest and then averaged across regions weighted by the area of each region.

**Fig. 3:**
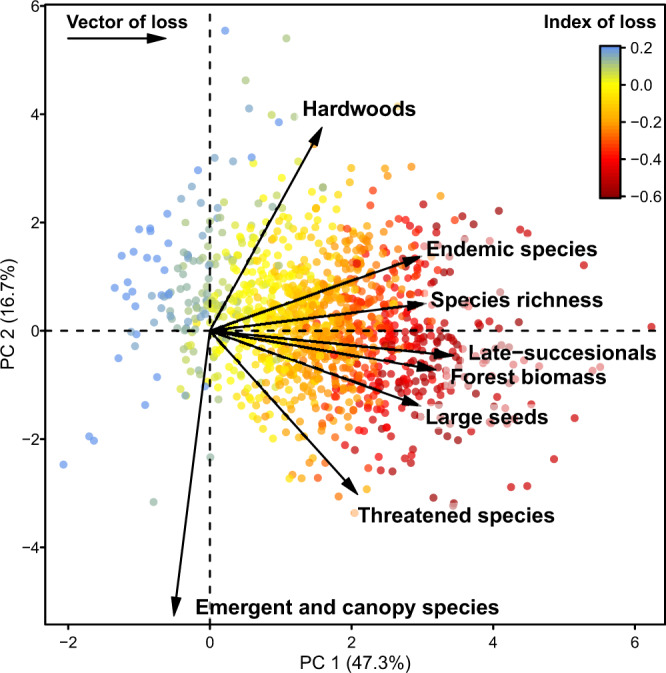
Human-induced impacts on tree species richness, multiple species properties, and forest biomass. Two-dimensional representation of the standardized indices of loss for each forest survey (points, *n* = 1154) plotted on the two first principal component (PC) axes, which explain 64% of the variation in the indices of loss. Arrows and their length represent the vectors of loss, i.e. the direction and strength of each individual index of loss. The more aligned the arrows, the more correlated the indices are. The average of all indices of loss is highlighted by a colour scale for each survey ranging from dark red (high losses) to blue (gains).

Human-induced impacts also caused a decline in the abundance of late-successional, large-seeded, and endemic species (Fig. 1), with reductions of 25‒42% when compared to low-disturbance Atlantic Forests (Supplementary Table 5 and 6). Shifts in species composition caused by human impacts have been reported for tropical forests, including the Atlantic Forest^3,7,19^. Here we also found greater shifts in species properties in surveys with greater losses of species richness and biomass (Fig. 3, Supplementary Figs. 9‒11). This means that the erosion of richness and biomass is being accompanied by a parallel decline of species that can enhance the provision of ecosystem services^24,25^ and safeguard the conservation value of the Atlantic Forest. In the long run, these losses can reinforce each other^24^, leading to a greater erosion of biodiversity and biomass.

The highest impact we found was related to the decline in the abundance of endemic species, meaning that endemics are being replaced by species with wider geographical ranges. Because species with wider ranges tend to occur over a wider variety of environmental conditions (i.e. generalists)^26^, widespread species can benefit at the expense of endemic and more specialist species in degraded fragments. Eventually, this process leads to the decrease of beta-diversity through time and thus to the biotic homogenization^19,27–29^. Thus, our results support the argument that human-induced impacts are driving the biotic homogenization of Atlantic Forest fragments^19,30^. Our approach based on community-weighted means (CWMs) of species properties does not allow us to distinguish which groups of species are causing this homogenization, but we can draw some propositions. Widespread tree species in the Atlantic Forest are often small-seeded pioneers that proliferate at forest edges and small fragments^19,31,32^. However, we found only a weak correlation between the losses of endemism level and of seed mass and ecological groups (*r* = 0.14 and 0.17, respectively; Supplementary Fig. 11). This suggests that not all species proliferating in disturbed Atlantic Forests are small-seeded pioneers. In addition, because exotics represented only 0.3% of the trees in our dataset, the proliferation of widespread native species is the most probable cause of the Atlantic Forest homogenization.

Wood density and maximum tree height, both related to carbon storage potential, presented the smallest changes. The latter even presented a positive mean index of loss, which may be explained by an increase in the abundance of late-successional, understorey species as human-impacts decrease. In the Atlantic Forest, species with those characteristics (common within Celastraceae, Erythroxylaceae, Myrtaceae, and Rutaceae) often have wood densities above 0.7 g cm^−3^, explaining the negative correlation found between maximum height and wood density losses (Fig. 3, Supplementary Fig. 11). Although taller species often have larger seed mass and higher wood density across vascular plants^33,34^, within trees there is still a wide spectrum of trait variation related to different regeneration strategies. Tree species that demand high irradiance for their development tend to have smaller seed sizes than species able to regenerate under mature canopies^35^, which was confirmed here by the relatively high correlation (*r* = 0.51) between the indices of loss of seed mass and ecological groups (Supplementary Fig. 11). Moreover, some canopy and emergent trees are long-lived pioneers, which have relatively light wood and small seeds^35,36^ (e.g. *Albizia*, *Ceiba*, *Ficus*, *Gallesia*, *Jacaratia*, *Parkia*, *Phytolacca*, *Piptadenia, Tachigali*). Altogether, these results suggest that declines in forest carbon stocks can be more easily explained by changes in forest structure than in its trait composition^37^.

Overall, our results show that postdeforestation, human-induced impacts are pervasive across the Atlantic Forest for species richness, forest biomass and most of the species properties. However, about 17% of the sites presented positive indices of loss (i.e. gains) of species richness or forest biomass and 5% presented gains in richness and biomass simultaneously (Figs. 1 and 2, Supplementary Table 4). Increases in species diversity in disturbed forests can occur depending on the frequency and intensity of the disturbances, by a balance of the contribution of early successional and late-sucessional species^38,39^. Increases in carbon storage potential can also follow the increase in the availability of resources due to global changes, such as atmospheric CO_2_, solar radiation and rainfall^40^; although these gains are small compared to losses caused by forest disturbances^41^. Our modelling approach to quantify human-induced losses is an alternative to overcome the lack of predisturbance data for the Atlantic Forest (see Methods for more details). This is a limitation of this study because ideally measurements before and after human disturbance should be used. Even though the goodness of fit measures of our models were fairly high for species richness and forest biomass (pseudo-*R*^2^ = 71 and 53%, respectively), model fits were not perfect and they will not generate accurate predictions for all surveys. If a given survey was conducted in a forest under a combination of climate, soil, and human-related conditions that were underrepresented in our dataset, predictions in the human-free scenario may be smaller than the observed values. Thus, caution must be taken when interpreting the index of loss for individual surveys.

### Influence of protected areas and human pressures

**Fig. 4:**
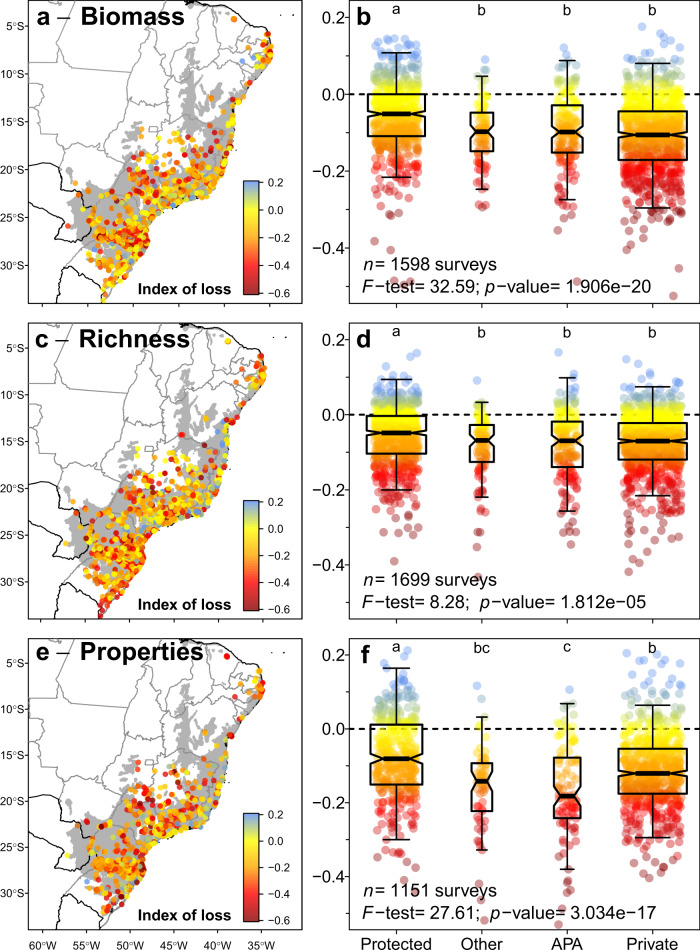
The distribution of the indices of loss across the Atlantic Forest and the effects of land conservation category. Each point represents one forest survey for which the index of loss was estimated for forest biomass (**a**, **b**), tree species richness (**c**, **d**) and species properties (**e**, **f**). In panels **a**, **c**, and **e**, the shaded area is the original extent of the Atlantic Forest. In panels, **b**, **d**, and **f**, the box-and-whisker diagrams summarize the distribution of the indices of loss for each land-use category (i.e. bold centre line, median; box limits, upper and lower quartiles; whiskers, 1× interquartile range), organized from left to right in decreasing order of protection. The *F*-statistics, the associated *p*-value, and the number of observations of the models are presented, as well as the result of the Tukey’s honest significance test (one-sided, with an adjustment for group sample sizes) for difference among group means (lowercase letters above each box-and-whisker diagram). Degrees of freedom: 1579 (**b**), 1698 (**d**), and 1150 (**f**). Legend: Protect indicates Strict protection and Sustainable Use conservation units; Other indicates other public and protected lands (e.g. research centres, university campuses, botanical gardens, military, and indigenous land); APA indicates private land inside areas with sustainable use of natural resources, locally known as “Environmental Protection Areas”; Private indicates private land.

**Fig. 5:**
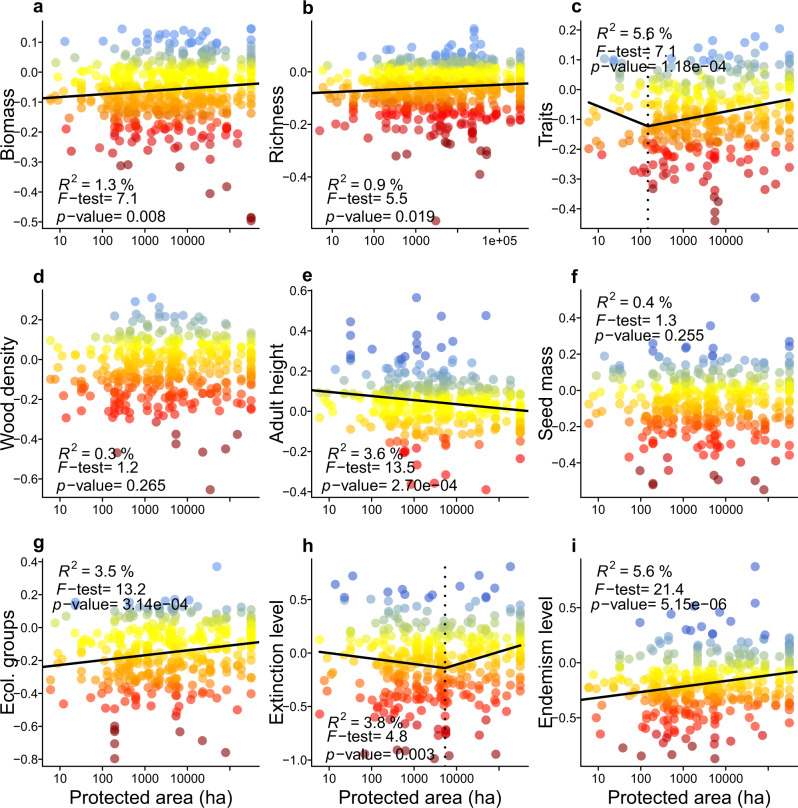
The effect of the size of the protected area on the loss of forest biomass, species richness, and species properties. For each survey (points), panels present the indices of loss of **a** forest biomass (*n* = 555 surveys); **b** tree species richness (*n* = 617 surveys); **c** weighted average species property loss; **d** wood density; **e** maximum height; **f** seed mass; **g** ecological groups; **h** threat of extinction; and **i** endemism level. The summary of the linear or of the piecewise regression models is given in the top of each panel, along with the adjusted *R*^2^ of the model, its summary *F*-statistics and the associated *p*-value (one-sided statistical test). Degrees of freedom for the regression models are: panel **a** = 554, panel **b** = 616, panels **c**–**h** = 364. The vertical dashed line is the estimated break-point of the piecewise model, which is plotted only for the variables were this model had a better performance than the linear model. The standardized index of loss is dimensionless and is highlighted by different colours ranging from dark red (high losses) to blue (gains).

Human-induced losses were lower inside than outside protected areas (Fig. 4) and they decreased as the size of the protected area increased (Fig. 5). Thus, large protected areas are important to reduce human-induced degradation, besides preventing deforestation itself. However, even inside protected areas, we detected pervasive losses of species richness, species properties and forest biomass (Fig. 4), revealing the practical limits of conservation policies focused solely on the establishment of protected areas^16^. In addition, human-induced impacts in areas where human settlements and use of resources are allowed but regulated (i.e. the Brazilian “Environmental Protection Areas”) were as high as, or higher than, in other areas of private land (Fig. 4). Thus, not all types of protected areas are equally effective at conserving biomass and biodiversity^15^. This pervasiveness of human impacts, irrespective of the land protection category, implies that strengthening the regulation within and surrounding existing protected areas is as important as the creation of new protected areas^16^.

**Fig. 6:**
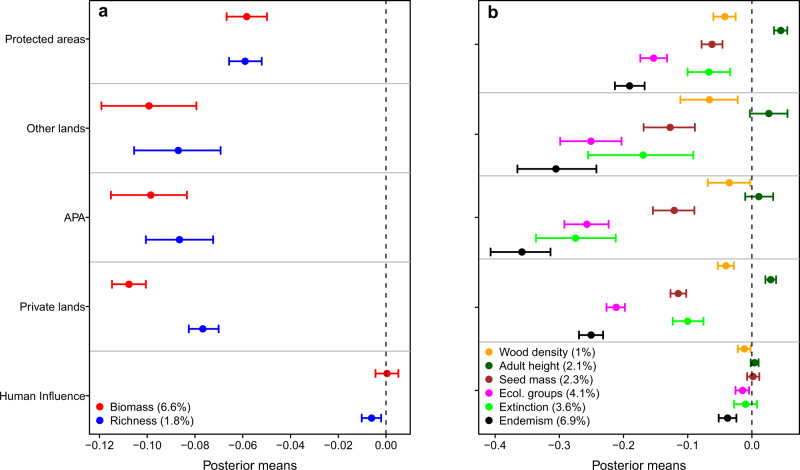
The effect of land conservation category and of the Human Influence Index on the carbon and biodiversity losses in the Atlantic Forest. Each point represents the posterior mean of the standardized effect sizes of the multivariate regression models containing the joint losses of **a** forest biomass and species richness (*n* = 1647 surveys) and **b** of community-weighted means of species properties (*n* = 1213 surveys). For the categorical variable ‘land conservation’, posterior means refer to the estimates of the model intercept, while for the continuous variable ‘human influence’ the means are the estimates of the model slope. The error bars (i.e. 95% credibility intervals) touching the dashed line reflect the lack of support of a significant effect of the covariables. The coefficient of correlation of each model (*R*^2^) is reported in parentheses. Legend: Protected areas indicate Strict protection and Sustainable use conservation units; Other lands indicate research centres, university campuses, botanical gardens, and military and indigenous lands; APA indicate private lands inside areas with sustainable use of natural resources, locally known as “Environmental Protection Areas” (APA is the acronym in Portuguese).

Although the land conservation category explained a small portion of the variation in all indices of loss, this factor explained more variation than other metrics of human pressure (Fig. 6). The Human Influence Index (HII)^42^ around the surveyed fragments had small or no effects on the magnitude of human-related impacts (Fig. 6). This is unexpected since the HII combines different vectors of human pressure and accessibility to forest fragments. Exchanging the HII with the distance of fragments from main cities (>100,000 inhabitants) did not increase the explanatory power of this analysis (not shown). This result may be explained by (i) the highly fragmented nature of the Atlantic Forest: few fragments are very large, undisturbed and really “far from” human presence, and by (ii) the nature of the human impact itself: disturbed fragments are the combination of different and idiosyncratic histories of land-use changes and degradation. For instance, urban forests can be in better conditions than suburban ones because they represent a vital resource for society (both aesthetic and monetary). On the other hand, small and isolated fragments in private lands can be well-conserved and diversified, depending on the understandings of the land-owner regarding the natural environment or whether the surrounding community has access and can use the forest resources. Other authors also found that the effects of human impacts in tropical forests can be quite unpredictable at regional scales^43–45^. Therefore, human impacts in the Atlantic Forest are the result of a complex history that makes current patterns of biodiversity and biomass loss remained difficult to predict in space from human pressure indices. Nonetheless, we found differences in average losses across the Atlantic Forest biogeographical regions for most indices of loss (Supplementary Fig. 12), reinforcing the idea that strategies to overcome biodiversity and carbon loss in the Atlantic Forest should be planned regionally^30^ (see discussion below).

### Total carbon losses in the Atlantic Forest

The erosion of biodiversity and biomass was pervasive across the entire Atlantic Forest hotspot. We projected the biomass loss across the remaining Atlantic Forest area and obtained losses of 451–525 Tg of carbon—equivalent to the deforestation of 55−70 thousand km^2^ (Table 3, Supplementary Fig. 13, Supplementary Table 7). This is about 1.4 times the carbon loss due to deforestation of the Atlantic Forest between 1985 and 2017, and about a quarter of its remaining forest area (26%). Assuming a value of US$5 per Mg C in international markets, this human-induced carbon loss translates into US$2.3−2.6 billion in carbon credits alone (see Methods). Although carbon-related impacts can be priced, biodiversity loss is far more difficult to value. The loss of a species locally does not necessarily translate into regional extirpation. Moreover, impacts on tree diversity may be protracted over decades^5^ and the recovery of tree diversity and species composition occurs at a much slower rate than forest biomass^9^. Biodiversity losses can be valued indirectly from their negative impacts on ecosystem services^20,24,25^, but the underpinning of these relationships is currently not understood well-enough to provide accurate economical assessments, particularly regarding shifts in species properties within ecological communities.

**Table 3:** The average loss of forest carbon and its projection to the remaining Atlantic Forest area.

Dbh cut-off (cm)	Carbon loss (%)	Equivalent forest loss (km^2^)	Equivalent carbon loss (Tg C)	Carbon credits (Billion US$)
≥5	25.3	55,082	451.3	2.257
≥10	32.0	70,279	524.5	2.622

The proportion of carbon loss was obtained per biogeographical region and then multiplied by the remaining forest area of each region to obtain the equivalent forest loss, i.e. the Atlantic Forest area that would match the carbon losses caused by postdeforestation human impacts. The equivalent carbon loss was computed based on the equivalent forest loss and the reference values of carbon storage per biogeographical region. We assumed a value of US$5 per Mg C paid for carbon credits obtained from projects of forestry and land use.

### Implications for the Atlantic Forest restoration

**Fig. 7:**
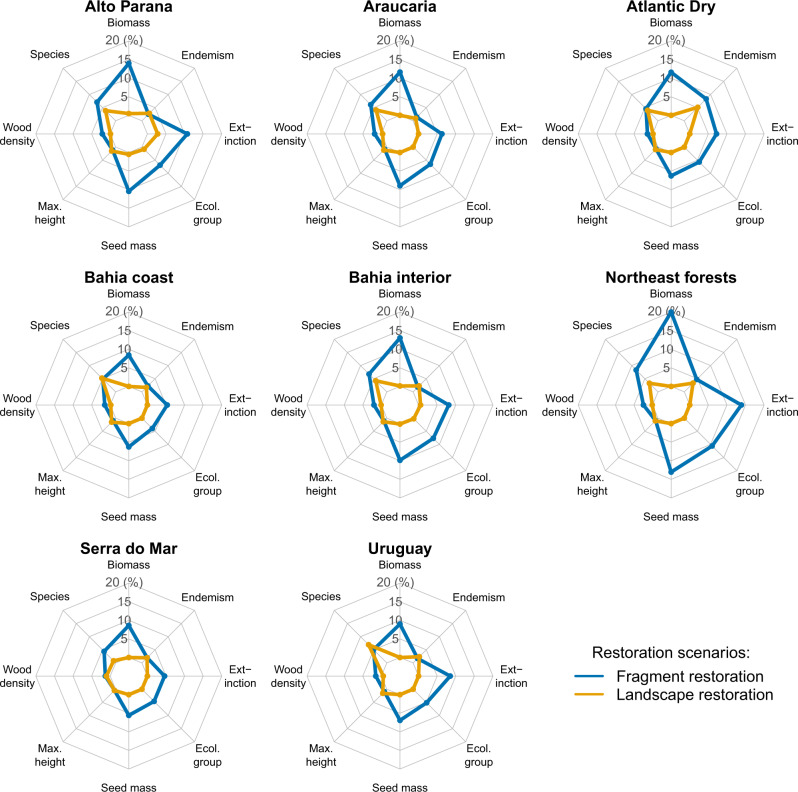
Multiple outcomes of two restoration scenarios of the Atlantic Forest. In each radar plot, we provide the proportional gain of forest biomass, species richness, wood density, maximum adult height, seed mass, ecological groups, extinction, and endemism levels for the ‘Fragment restoration’ (blue line) and ‘Landscape restoration’ scenarios (orange line). Gains (in %) are the difference between each restoration scenario and the ‘current scenario’ (see Methods), which are presented separately for each biogeographical region of the Atlantic Forest.

To explore strategies to mitigate biodiversity and carbon losses in Atlantic Forest remnants, we used our models to compare the outcomes of two contrasting restoration strategies: ‘fragment restoration’ that aimed at halving the within-fragment disturbance levels of Atlantic Forest remnants; and ‘landscape restoration’ that aimed at restoring 20% of landscape forest cover around them. The first scenario aimed at simulating restoration activities such as the reduction of forest-edge effects, control of invasive species and enrichment plantings, while the second aimed at increasing fragment size and thus landscape connectivity, e.g. forest corridors (see Methods for details). We found that proportional gains were greater for the ‘fragment restoration’ scenario for all variables, except for wood density, maximum height and endemism levels (Fig. 7). This means that the most effective strategy to restore the remaining Atlantic Forest fragments is to reverse forest degradation inside them. This statement was particularly true for forest biomass, seed mass, ecological groups and extinction level (Fig. 7). Despite being an expected result, it reinforces the role of forest disturbances as an important driver of biodiversity and biomass losses^13,14^.

We found a tendency for gains to be proportionally higher in the Alto Paraná and Northeast forests than in other regions of the Atlantic Forest (Fig. 7). These areas are within the more fragmented and disturbed regions of this biodiversity hotspot, meaning that restoration efforts there would provide better outcomes regarding carbon and biodiversity recovery within existing fragments. Consequently, these regions also presented fragment restoration yields that were comparatively higher (Supplementary Table 6). Thus, the fragment restoration strategy in these regions should be more cost-effective than in other Atlantic Forest regions, which adds up to our knowledge about how to prioritize restoration efforts in the Atlantic Forest^46^. The Atlantic Dry region was also among the regions with the highest proportional carbon gains in the fragment restoration scenario, but in this case due to its higher forest cover and smaller carbon storage potential (less restored area combined with less carbon gain per hectare to attain the 20% forest cover target of the landscape restoration scenario). In contrast, in the Serra do Mar and Bahia Coastal regions, which combine higher carbon storage potentials with lower disturbance levels, the expected outcomes of restoration within fragments were among the lowest when compared to restoration of the surrounding landscape. On average, these areas have higher landscape resilience and thus lower implementation costs of restoration, making landscape restoration proportionally more efficient.

The ‘landscape restoration’ scenario, on the other hand, can recover much more carbon at the landscape-scale, since it includes the restoration of non-forest lands. Predicted carbon gains and restoration yield (i.e. carbon gains divided by total costs of restoration) for the ‘fragment restoration’ scenario were about 5‒9% and 19‒33% of the gains and yield for the ‘landscape restoration’ scenario, with restoration outcomes depending on the fragmentation and disturbance levels of the different Atlantic Forest regions (Supplementary Table 6). The ‘landscape restoration’ strategy can thus sequester carbon more efficiently, but it had little impact in reducing forest degradation within the remaining forest fragments (Fig. 7). Thus, restoration will require regionally-planned strategies that combine landscape with fragment restoration to efficiently attenuate biodiversity and carbon losses in human-modified tropical forests^30^.

The two restoration scenarios predicted small improvements for hardwood, tall and/or endemic tree species within fragments (Fig. 7). Greater improvements would probably require the restoration of fragments to their full predisturbance conditions and targeting landscape restoration to forest covers much larger than 20%. A 50% of landscape forest cover can decrease the uncertainty in restoration success^47^, but establishing such a high target for the restoration of rural Atlantic Forest landscapes would probably not be economically feasible. In this context, selecting the appropriate species to maximize restoration outcomes becomes a more efficient strategy^48^. To help the species selection for restoration we provide a list of 242 common tree species (Supplementary Data 2) that have a high potential to increase the carbon storage and the conservation value of disturbed Atlantic Forest fragments (see Methods for details). The reintroduction of these species could mitigate the effects of local extirpation in the short term, while the connectivity of the landscape can be restored and allow the frugivore fauna to return in the long run. Seedlings from hardwood, tall or endemic species may be less available from nurseries and thus increase restoration costs^48,49^. So, local seed production cooperatives and nurseries able to propagate these species will be essential for the support of the Atlantic Forest restoration^48^, particularly regarding the reintroduction of large-seeded Atlantic Forest endemics.

This is a simplified exercise of the possible outcomes of contrasting restoration strategies because it assumes that all fragments/landscapes will have high restoration success and follow the same restoration trajectories^50^. A more complete appraisal would require spatialized, fragment-specific information on the disturbance level, landscape conditions, restoration success/unpredictability, and land opportunity costs, as well as a more complete and detailed assessment of the impact of the different restoration scenarios and restoration targets on species survival and on ecosystem services other than carbon storage (e.g. refs. ^46,47,51^). However, these two simple scenarios provided an approximation of costs and how outcomes may vary across the Atlantic Forest. In addition, they allowed us to indirectly explore the role of within-fragment disturbances and patch/landscape metrics as drivers of biomass and biodiversity recovery inside Atlantic Forest remnants.

### Implications for biodiversity and carbon conservation

The imprint of the human-induced degradation on the Atlantic Forest may be an indicator of the future condition of other tropical forests. Thus, the fate of tropical forests depends not only on avoiding the deforestation of intact forests or on promoting the reforestation of degraded lands^9,12,52^, but also on mitigating forest degradation in the remaining forest fragments^10,13,17,25^. The impacts of forest degradation are hard to quantify at regional scales and have therefore received less priority in the climate change and conservation agendas. Despite recent changes in the Brazilian government’s environmental priorities, the Brazilian pledges to mitigate greenhouse gas emissions (e.g. Atlantic Forest pact, Paris Agreement or Bonn challenge) do not include actions to reverse forest degradation or to re-introduce species with certain properties (e.g. species with a high conservation value or carbon storage potential). In human-modified landscapes, the mitigation of forest degradation can be a more cost-effective strategy than reforestation depending on the outcomes desired. Moreover, it conflicts less with other land uses, such as agriculture^53^ and thus has good potential for engaging stakeholders.

Therefore, besides safeguarding part of the biodiversity and catalysing the restoration of nearby areas^54^, restoring disturbed fragments is an opportunity to enhance tropical biodiversity and biomass. In regions such as the Atlantic Forest, where most of the forest remnants are on private land^21^, this opportunity has direct ramifications implications to the compensation mechanisms for climate change mitigation. In Brazil, funds to reduce carbon emissions from deforestation and forest degradation (REDD+) are mainly concentrated on Amazonia and focused on avoiding deforestation (e.g. the currently inactive Amazon Fund). Currently, only the State of Rio de Janeiro has a fund for the Atlantic Forest (http://www.fmarj.org). Our results indicate that the potential for a biome-wide compensation fund is in the order of billions of dollars. The creation of national policies to mitigate forest degradation, although highly dependent on the vision and will of politicians, could be the key to attracting funds to the Atlantic Forest and therefore be decisive for the future of this biodiversity hotspot^55^.

## Methods

### Study region

The Atlantic Forest is a global biodiversity hotspot that once covered 1.63 million km^2^ mostly in Brazil (92% of the total area), but also in Paraguay (6%) and Argentina (2%—Supplementary Fig. 1). It covers a wide range of climatic and edaphic conditions, with forest types ranging from rainforests to seasonal forests, including cloud, swamp, and white-sand forests^56^. The Atlantic Forest has been suffering from deforestation and degradation for over 500 years. Today, it includes some of the largest cities in South-America, with over 148 million people currently living within the Atlantic Forest limits^57^. Less than 20% of the original Atlantic Forest remains and the remnants are characterized by small (<50 ha), isolated and altered fragments^21^. Although deforestation started centuries ago, the main period of fragmentation history occurred between the beginning of the 20th century and the 1970s, and deforestation rates have declined in the past two decades^58^. Regional differences in human population density, land conversion patterns, and cultural aspects have created a great diversity of landscapes in terms of forest cover, fragment size, and disturbance levels^21,59^.

### Forest surveys

We obtained 1819 tree community surveys of natural Atlantic Forests available from the Neotropical Tree Community database (TreeCo)^23^. We considered all types of forest formations in Brazil, Paraguay and Argentina (Supplementary Fig. 1, Supplementary Data 1), except for dry deciduous and early-secondary forests. Moreover, we considered only surveys including trees with diameter at breast height (dbh) ≥3, ≥5, and ≥10 cm and using plots or the point-centred quarter method. The surveys used in the analysis ranged from 0.03 to 26 ha in sampling effort (mean ± standard deviation: 0.68 ± 1.46 ha), with the majority being conducted in evergreen and semi-deciduous forests (73%), the two main Atlantic Forest formations^56^. They represented a total effort of 1.45 million trees and 1238 hectares (Table 1) and they covered a wide spectrum of environmental conditions and patch/landscape metrics (Supplementary Table 1). Sampling design and methods varied greatly across surveys, but this variation was taken into account during the analyses (see details below).

For each survey, we extracted the tree density (trees ha^−1^), basal area (m^2^ ha^−1^), species richness, sampling method (plots and point-centred quarter method), arrangement of sample units (contiguous and systematic/random), sampled area (ha), dbh inclusion criteria (cm) and geographical coordinates. We verified the precision of the geographical coordinates provided to ensure that they corresponded to the forest fragment studied, otherwise the survey was discarded. Whenever needed, plot coordinates were corrected, based on maps or the site description provided in the study, including internet searches of any valuable information on the fragment, farm or park location.

### Species data

We extracted data on species composition and abundances for surveys with a total sampling area of at least 0.1 ha, and with species data presented in an extractable format (i.e. complete phytosociological tables). The sample size cut-off ensures a minimum representativeness of the composition of the forests surveyed. The second filter is related to the fact that many quantitative surveys present the phytosociological table partially (e.g. only the most abundant species are given) or not at all. Also, the table was sometimes given in full but without the information needed to run analyses (e.g. tables presenting only the species name and its importance value). In those cases, we tried to contact the original authors of the publications asking for complete phytosociological tables, but the rate of success of our emails was low. Surveys including species data represented 72% of the 1819 surveys, but they contained 84 and 81% of the sampling cover and number of trees, respectively.

For all surveys providing information on voucher specimens associated with morpho-species, we checked for identification updates performed by taxonomists using the speciesLink network (http://splink.cria.org.br). Although only about one-third of the surveys provided vouchers, this effort improved the taxonomic resolution of around 10% of our records. We checked species names for typographical errors, synonyms, and orthographical variants, following the Brazilian Flora 2020^60^. Names marked as confer were assigned to the species suggested for confirmation, while those marked as *affinis* were considered at the genus level. We compiled 98,030 species records, totalling 1,171,935 trees measured and 3124 valid species names.

### Species properties

For the 3124 species included in the database, we compiled information on the following species properties: wood density (g cm^−3^)^61^, maximum adult height (m)^62^, seed mass (grams)^63–65^, ecological group (or successional group), IUCN threat category^66^, and endemism level. These properties are related to species’ role in carbon storage (i.e. provision of ecosystem services), ecological interactions and strategies (i.e. ecological functioning), and biodiversity conservation (i.e. species conservation value). Besides being relatively easy to obtain from the literature, these six properties had good coverage at the species level for Atlantic Forest species. Besides the references cited above, over 300 different sources of information were consulted to complete these species properties (see Supplementary Data 3).

Ecological groups (i.e. pioneer, early secondary, late secondary, and climax) were also obtained from information provided in the original surveys. The threat category was obtained from red lists at national and global scales and in case of inconsistencies, we used the category provided in the national red list. Endemism levels were defined based on species occurrences in different continents, countries and Brazilian states^60,62^. Species with Pantropical, Neotropical and South American distributions were classified as ‘not endemic’, while species restricted to one or two adjacent regions (e.g. São Paulo and Rio de Janeiro states) were classified as ‘local endemic’. Species restricted to South, South-eastern or North-eastern Brazil were classified as ‘regional endemic’. Records retrieved for numerical properties were averaged for each species. Maximum adult height was considered here as the 90% quantile of the distribution of maximum height records. For wood density and seed mass, we used genus level instead of species-level averages if necessary^67,68^. We also completed missing information on ecological groups for typical pioneer Neotropical genera (e.g. *Cecropia*, *Trema*, *Vernonanthura*). The final proportions of species with information available (at species or genus level) were: 99.5% for wood density, 93.4% for maximum height, 95.9% for seed mass, 65.4% for ecological groups, 100% for extinction threat status and 96.9% for endemism level.

We computed community-weighted means (CWM) for the species properties to summarize the community composition sensu lato of each survey. For continuous species properties, the CWM was simply the average of each property weighted by the total number of individuals in the community. For wood density and maximum height, we used the total basal area instead of the number of individuals of each species to calculate the CWM. For wood density, CWM was obtained after removing palms, palmoids, cacti, and tree ferns. For maximum height, we removed shrubs before the calculation of CWM. For seed mass, we removed tree ferns prior to the calculation of CWM.

To compute the CWMs for the categorical properties, we treated them as ordinal categorical data: Pioneer < Early secondary < Late secondary < Climax for ecological groups; Not threatened/Not evaluated/Least concern < Data deficient/Near threatened < Vulnerable < Endangered < Critically endangered for extinction level; and Exotic/Naturalized < Not endemic < Northern/Eastern/Southern South-America < Regional endemic < Local endemic for the endemism level. We assigned scores to these ordered categories and used them to compute the CWM. For ecological groups, we used the following scores: Pioneer = 1, Early secondary = 2, Late secondary = 3, Climax = 4. For the threat categories, we used: Not threatened/Not evaluated/Least concern = 0, Data deficient = 0.5, Near threatened = 1, Vulnerable = 2, Endangered = 3, Critically endangered = 4 for extinction risk. Finally, we scored species endemism level as follows: exotic/naturalized = −1, not endemic = 0, Northern/Eastern/Southern South-America = 1, regional endemic = 2, local endemic = 3. For the latter, we assumed that the presence of exotic species has a negative score of endemic species. These scores were chosen so that the higher the CWM, the better the community is regarding these species’ properties.

### Site descriptors

For each survey we obtained climatic (e.g. mean air temperature and rainfall) and topographic information (i.e. altitude, slope declivity, and aspect) from different sources (see Supplementary Methods). We also obtained soil classes from the original surveys, which were checked for their consistency using soil maps at state and national scales. Missing data and inconsistencies between sources were double-checked to assure soil data quality and homogeneity. We used soil classes to infer average soil properties for plant growth by cross-referencing them with a database of physical and chemical soil properties (see Supplementary Methods). We used forest cover maps (30 m resolution)^1^ to extract 4 × 4 km landscapes centred on the survey coordinates and we used a 70% canopy closure threshold to classify maps into forest or non-forest pixels. Classified maps were used to calculate the proportion of forest cover and core forest cover, the median edge-to-edge distance between fragments and different landscape aggregation indices (see Supplementary Methods for details).

Fragment size was obtained from the original publications and was cross-validated using the 2002 and 2012 maps of the Atlantic Forest fragments^58^. We completed missing values of fragment size if there was consistency of the fragment size obtained from these maps with the one obtained from the classified 4 × 4 km landscapes. Inconsistencies between these sources were solved in Google Earth Pro (© Google Inc.), including the manual recalculation of fragment size, which we conducted for ~400 surveys. The computation of the (mean) distance between surveys and forest edges was not possible for the majority of surveys for different reasons (see Supplementary Methods for details). Forest-edge effects are inextricably linked to fragment size and shape. Thus, their effects are indirectly accounted for in other landscape and patch metrics.

Forest disturbance level was assigned based on the information on the type, intensity and timing of human disturbances (i.e. selective logging, fire, hunting, thinning) provided by the authors of the surveys. We considered three levels of disturbance: high (i.e. highly/chronically disturbed forests, typically disturbed less than 50 years before the survey); medium (lightly/sporadically disturbed forests, and/or disturbed 50–80 years ago); and low (forests left undisturbed for at least 80 years). This classification is qualitative, with substantial variation in forest structure and diversity expected within classes. However, more objective and detailed information on disturbance histories, main disturbance types and their intensities was lacking (see Supplementary Methods). Thus, these coarse classes are the best information available to allow analysis across the Atlantic Forest.

We assigned each survey a biogeographical region^69^. To avoid an excessive subdivision of regions, we reassigned Atlantic Forest surveys mapped as Cerrado, Caatinga, Campo Rupestre enclaves, Atlantic Coast Restingas and Southern Atlantic Mangroves to the closest region (Supplementary Fig. 1). We included the Uruguayan biogeographical region, representing the forests in the transition to the pampas region of South Brazil as part of the Atlantic Forest^21,58^. The proportion of each region in our sample was similar to their contribution to the remaining Atlantic Forest area with few exceptions (Serra do Mar: 29% in the dataset and 21% of the remaining area, Araucaria: 24 and 13%, Alto Paraná: 21 and 31%, Bahia Inland: 8 and 11%, Bahia Coastal: 6 and 7%, Uruguay: 5 and 3%, Northeast: 5 and 4%, Atlantic Dry: 2 and 10%).

Categories of land conservation were obtained from the original study and/or from maps of conservation units, as follows: protected areas (i.e. strict protection and sustainable use conservation units); other public and protected lands (e.g. research centres, university campuses, botanical gardens, military and indigenous lands); private land regulated by government laws, a conservation unit known in Brazil as Environmental Protection Areas (or APA for Portuguese: “Área de Proteção Ambiental”); and private lands outside conservation units (e.g. farms). We could not assign the land-use category for 5% of surveys. For surveys conducted inside protected areas, we also obtained the size and type of the protected area (e.g. strict protection, sustainable use).

We extracted the Human Influence Index (HII)^42^ based on the coordinates of each survey. The HII ranges from 0 to 64 (minimum and maximum human influence, respectively) and it accounts for human population density, land use (e.g. urban areas, agriculture) and ease of access (i.e. proximity to roads, railways, navigable rivers, and coastlines). The survey distance from main cities (>100,000 inhabitants) was significantly correlated with the HII (Pearson’s *r* = −0.38, *p* < 0.0001). Because results using this distance instead of the HII were qualitatively the same, we present only the results using the HII.

### Data analysis

We conducted separate analyses for each response variable, because not all surveys had data on forest biomass, species richness and CWM simultaneously. We also removed 50 surveys from the analyses of species properties, because in these surveys less than 80% of the sampled individuals had information on species properties. Thus, analyses were conducted using 1676 surveys for biomass (92% of surveys), 1790 for species richness (98%), and 1213‒1214 (68%) depending on the species property considered (Supplementary Data 1). Response and explanatory variables were transformed if necessary and candidate explanatory variables were preselected based on their colinearity and on their impact on model performance (see Supplementary Methods).

We described forest biomass, species richness, and CWMs of species properties using linear mixed-effects regression models. To select which explanatory variables should be included in these models^70,71^, we first selected the covariables composing the random structure of the models, which had survey methodology and biogeographical regions as candidate random effects. These two categorical variables divide the observations into groups, to account for correlated observations in the data^70,71^ (e.g. species richness is higher and more similar for some biogeographical regions when compared to other regions). Survey methodology refers to the sampling method, arrangement and dbh inclusion criteria, which were combined to create a methodological categorical variable (e.g. contiguous plots dbh ≥5 cm, systematic plots dbh ≥10 cm, etc). Exploratory data analyses suggested an interaction of the effects of sampling effort and the methodological variable on forest biomass and species richness. Indeed, the addition of a random term composed by the log-transformed sampling effort nested within the methodological categories significantly improved the model fit for both variables. To avoid the artificial inflation of model explanation^71^, we also included log-transformed sampling effort as a fixed effect in the final models of biomass and richness. The same was not true for the random structure of the models of species properties, which had fewer observations and were more prone to overfitting issues (i.e. model singularity).

We then selected the fixed effects to compose the optimum regression model. We kept to a minimum the interactions between fixed effects to avoid problems with the interpretation of individual coefficients, including only interactions with a clear biological meaning (e.g. temperature × rainfall seasonality). The optimum regression models had the following general structure: *y* ~ *Environment* + *Human* + *Effort* + (*Effort* | *Method*) + (1 | *Region*). The term *Environment* includes the climate, topography and soil variables (and their interactions). *Human* includes landscape metrics, fragment size, and disturbance level. *Effort* is the total survey effort (not included in species property models). As described in the previous paragraph, *Method* are the methodological categories (nine levels) and *Region* are the Atlantic Forest biogeographical regions (eight levels, Supplementary Fig. 1). Therefore, models had environmental, human, methodological and biogeographical (historical) components. The number of individuals sampled had, as expected, a strong influence on species richness. During explanatory analyses, we also found a positive relationship between biomass and tree density (individuals ha^−1^). So, we included the number of individuals and tree density as fixed effects to model richness and biomass data, respectively.

The optimum regression models contained only fixed effects that improved overall model fit. To obtain this optimum model, we used a model selection procedure based on the Akaike information criterion (AIC) of the candidate models to select the best structure of fixed and random effects. Differences in AIC values greater than log(8) between candidate models were regarded as an indicator of differences in model fit^72^. For the final regression model obtained for forest biomass, species richness and species properties, we tested the significance of the full model based on the comparison of a “null” model containing only random effects and sampling effort, using Chi-squared statistics (Supplementary Fig. 8). We also obtained the conditional (pseudo)*R*^2^, which may be seen as the variance explained by the full model (fixed + random effects^73^). All statistical analysis was performed in R^74^ using packages lme4^75^, piecewiseSEM^76^, MuMIn^77^, and r2glmm^78^. Models describing the CWM of species properties had lower explanatory powers than forest biomass and tree species richness (Supplementary Table 2), particularly for carbon-related traits. Compared with biomass and richness, species composition is more dependent on site history, random dispersal events, and species interactions (negative or positive) and thus less predictable^79^.

### Evaluation of human impacts

We used the optimum models to quantify human-related impacts on species richness, species properties and forest biomass. We first obtained the model predictions in a scenario without major human-induced impacts, i.e. model predictions for human-related variables reset at values corresponding to large (>300,000 ha), low-disturbance patches in landscapes with 100% of (core) forest cover and maximum patch aggregation. We assumed current conditions for all other covariables in the models (i.e. climate, soil conditions, sampling method, and biogeographical region) and the deforestation caused by indigenous populations to be negligible. Then, we calculated the standardized difference between observed values and predicted values in the human-free scenario:

$$\left( {{\mathrm{Observed}}-{\mathrm{Predicted}}} \right)/{\mathrm{Predicted}}.$$

The difference between observed and predicted values was standardized to make them comparable across our response variables. We used this standardized difference as an index of loss related to human-induced impacts (Fig. 1). Negative indices mean that observed values of biomass, species richness or community-weighted means (CWM) of species properties are smaller than the predictions in the human-free scenario at the same forest fragment.

This modelling approach used to quantify human-induced impacts was designed to deal with the lack of measures before and after human impacts. Large-scale deforestation and degradation of the Atlantic Forest began in the late 19^th^ century and intensified between the 1940s until the 1970s^80^. However, nearly all Atlantic Forest surveys available were conducted from the 1980s onwards^23^. Thus, it is impossible to obtain measurements of undisturbed Atlantic Forests, particularly at the scale of our study (~1.4 million km^2^). Even remote sensing data is only available since the 1970s^80^, when the Atlantic Forest was already highly modified. So, we assumed Atlantic Forests previous to human impacts to be large, fully connected and undisturbed. This assumption has limitations but it is a valid yet conservative representation of past forest conditions.

We compared the predictions for the scenario without major human-induced impacts against intervals obtained from 5000 samples of the estimated model coefficients (see Supplementary Methods). The precision of these predictions was not strongly sensitive to variations in the parameter estimates (Supplementary Table 4). In addition, predictions of carbon stocks for the human-free scenario ranged between 43‒162 Mg C ha^−1^ for individual surveys, but they were typically between 88‒110 Mg C ha^−1^ (mean of 99 Mg C ha^−1^), which is consistent with previous studies in the Atlantic Forest^80,81^. Finally, because we predict shifts in CWM of species properties and not in the taxonomic composition itself, our approach did not provide any indication of which species increased or declined in abundance due to human impacts.

The distribution of this standardized index of loss was symmetric for all variables (i.e. well approximated by a normal distribution—Fig. 1). The only exception was species richness that presented a left-skewed distribution and was better described by a Weibull distribution. We used the fit of these two distributions to estimate 95% confidence intervals around the mean index of loss, used here to assess the difference between the mean losses across our response variables. We then inspected the relationship among indices of loss by plotting one against the other (Fig. 2, Supplementary Figs. 9‒10) and testing the strength of their relationship using linear regression models. We also inspected the correlation among the pairs of indices of loss for the six species properties (Supplementary Fig. 11). We averaged the indices of loss for the six species properties to generate a mean index of loss, using the conditional *R*^2^ of the mixed-effects models of each property as weights (Supplementary Table 2). Next, we explored the relationships between all indices of loss using the two first axes of a principal component analysis, produced using the scaled indices of loss for species richness, properties and biomass (Fig. 3). Lastly, we plotted the indices of loss over the Atlantic Forest map to reveal possible hotspots of human impacts.

### Influence of protected areas and human pressures

We evaluated if the category of land use (i.e. conservation units, private lands, etc.) and the Human Influence Index (HII)^42^ affect on the indices of loss for forest biomass, species richness and trait composition. We performed this evaluation using univariate models (Fig. 4). This model is equivalent to a standard Analysis of Variance (ANOVA), so we computed the *F*-statistics of the model and the Tukey Honest Significant Differences between the indices of loss for each pair of land-use categories. This test was performed using a 95% confidence level. Moreover, because the indices of biomass and richness loss were significantly correlated (Pearson’s *r* = 0.22), we also tested if the use of multivariate regression models would improve our interpretation of the effects of land use and HII. A similar procedure was used for the indices of trait loss, which were often correlated as well (Supplementary Fig. 11). These models accounted for spatial autocorrelation between response variables and their parameters were estimated using Markov chain Monte Carlo (MCMC) methods^82^. The number of MCMC iterations, the thinning interval and the burn-in were 10^6^, 10^4^, and 10^3^. Priors were defined as the diagonal matrices for the covariances (V) and 1.002 for the degree of belief parameter (nu)^82^. The results from the bivariate models were qualitatively the same as those obtained using univariate models (Fig. 6).

We also evaluated the effect of the size and type of the protected area on the indices of loss (Fig. 5). More specifically, we tested the shape and strength of the relationship between the indices of loss and the log-transformed size of the protected area by comparing the fit of linear, quadratic and piecewise regression models. Piecewise regression was used to detect the existence of a critical size of protected areas that would minimize biomass, diversity, and trait losses. As above, the fit of models was compared based on AIC values and only the model with the best fit to data is reported (Fig. 5). However, we found evidence of a critical size of protected areas only for extinction level and mean trait loss. Piecewise regressions were fitted using the contributed R package segmented^83^.

Finally, we compared the differences in losses between conservation units classified as strict protection, on the one hand, and sustainable use of natural resources, on the other. We found differences neither in biomass and richness losses, nor for most of the traits. The two traits that presented evidence of differences were maximum height (smaller losses in lands under strict protection: adjusted *R*^2^ = 1.2%; *F*[d.o.f. = 363] = 4.67; *p* value = 0.031) and endemism level (smaller losses in lands under sustainable use of resources: adjusted *R*^2^ = 1.3%; *F*[d.o.f. = 363] = 4.87; *p* value = 0.028), but the strength of evidence was small.

### Reference values for undisturbed Atlantic Forests

We estimated the mean values of tree species richness (species ha^−1^), species properties (CWM) and forest basal area (m^2^ ha^−1^) for low-disturbance surveys, taken here as references to the species diversity, community composition and biomass storage of undisturbed Atlantic Forests (Supplementary Table 5 and 6). Here, we assumed modern, low-disturbance fragments as proxies of past forest conditions (i.e. forests with low human impacts). We acknowledge that past forests may have had more biodiversity and biomass than modern-day low-disturbance forests, but these fragments represent the best information currently available for the Atlantic Forest, given that measurements prior to human impacts are unavailable. Thus, we took a conservative approach; if modern-day fragments underestimate past conditions, absolute losses would be even greater than reported here.

Reference values are expected to vary among regions, so we estimated them separately for each biogeographical region of the Atlantic Forest. These reference values were also estimated using a different subset of low-disturbance surveys for each forest descriptor. For forest biomass, we included surveys of a total sampling effort of ≥0.2 ha. We used the equation provided in the Supplementary Material to convert basal area into mean above-ground biomass (Mg ha^−1^) and we assumed 47% of carbon concentration in the dry mass to convert above-ground biomass into above-ground carbon (Mg ha^−1^). To estimate species richness, we used only surveys of ~1 ha. For the CWM of species functional traits and conservation value, averages were obtained using only surveys with 500 or more individuals sampled.

Because the dbh inclusion criterion influences the forest description^84^, the reference values and consequently the average proportion of loss were calculated separately for each dbh inclusion criterion. We retrieved fewer surveys using dbh ≥3.0–3.2 cm (Table 1), and even fewer surveys of low-disturbance forests with total sampling effort of ≥0.2 ha or ~1 ha (Supplementary Table 5). Therefore, we conducted no projections of human-induced impacts based on forests surveyed using dbh ≥3.0–3.2 cm. For species traits, ecological groups and conservation value, dbh ≥5.0 was the most frequent cut-off criterion. Surveys using dbh ≥5.0 cm represent better the composition of the lower tree layer of the forest, which contains the individuals that will compose the future canopy of the forest. Considering the long lifecycle of tree species, we assume that trees between dbh 5.0 and 10.0 cm should reflect better the changes in forest composition. So, the reference values and average losses of species properties are reported only for dbh ≥5.0 cm (Supplementary Fig. 12, Supplementary Table 5).

### Absolute losses and projections across the Atlantic Forest

To express in absolute terms the losses due to human-related impacts, we calculated the absolute loss for each survey (i.e. Predicted–Observed), which was then averaged for each region. Because our sample is biased towards large forest fragments (Supplementary Fig. 2), these averages were weighted by the probability of having a fragment of the same size in each biogeographical region. These probabilities were obtained from log-normal distributions fitted to the 2016 size distribution of the remaining Atlantic Forest fragments^58^. We then calculated the proportion that these weighted average losses represent in respect to the region-specific reference values (Table 2, Supplementary Fig. 12).

To obtain the forest area that would match the carbon losses caused by postdeforestation human impacts (i.e. equivalent forest loss), the proportion of carbon loss was multiplied by the remaining Atlantic Forest area per biogeographical region^58^. Due to missing information on human-related variables for all Atlantic Forest fragments (e.g. within forest disturbance level), we assumed the same carbon loss for all remaining fragments within each biogeographical region. Next, we obtained the total amount of carbon loss (i.e. the equivalent carbon loss), which was computed based on the equivalent forest loss and the reference values of carbon storage per region. The mean carbon storage and proportional losses were averaged across the biogeographical regions, using their total area weights. In contrast, values of equivalent forest and carbon loss were summed across regions. Thus, our estimate of carbon loss and its projection across the Atlantic Forest takes into account the bias towards larger fragments in our sample and the regional differences in Atlantic Forest carbon storage potential (Table 3).

We compared the equivalent forest loss to Atlantic Forest deforestation between 1985 and 2017^58^. In the Brazilian Atlantic Forest, deforestation was 1.93 million ha for the entire interval of 1985 and 2017^58^. For Paraguay, we found estimates for 1989‒2000 and 2003‒2013^85,86^, so we estimated a 1.96 million ha forest loss for 1985‒2017 from the reported annual deforestation rates. For Argentina, we had estimates for 1998‒2014^57^ and used the same procedure to obtain an estimate of 0.1 million ha for 1985‒2017. It should be noted that overall deforestation in Brazil was larger than in other countries, but it occurred more intensely before 1985. Therefore, we estimated a total forest loss of 4.18 million ha between 1985‒2017. Finally, we calculated how much money the equivalent carbon loss would represent if it had been traded as carbon credits in international markets (Table 3), assuming US$5 per Mg C paid for projects of forestry and land use^87^.

Although the average proportion of species loss and changes in species properties were obtained, the projection of these proportions for the entire Atlantic Forest is more complex. Observed species richness is nonlinearly related to the sampling area^84^, so it cannot be directly extrapolated from smaller sample sizes to the entire Atlantic Forest area. Moreover, the loss of species from a local community does not necessarily mean that these species are locally or regionally extinct. Thus, the equivalent forest area lost due to losses in species richness cannot be calculated. Also, there is no market for “biodiversity credits”, so pricing species losses is very difficult. Trait composition is contingent on community species composition, which is highly variable across the Atlantic Forest hotspot. This also hinders the possibility of projecting and valuing trait composition losses across the entire study area.

### Simulating strategies of forest restoration

We used the optimum regression models fitted to biodiversity and biomass data to simulated two simplified scenarios of forest fragment restoration. The ‘fragment restoration’ scenario assumes no increase in landscape forest cover and aims at restoring forest disturbance levels to 50% of the lower level observed for the Atlantic Forest. This target is realistic, because restoring and maintaining disturbance levels below this target, although possible, would be too expensive. The ‘landscape restoration’ scenario mimics restoration activities around existing fragments, increasing fragment size and thus landscape integrity (i.e. greater forest cover) and connectivity (i.e. smaller fragment distance). This scenario implicitly assumes that restoration activities should focus on the restoration around existing forest fragments, which would increase the ‘area’ of restoration and thus maximize the success of the restoration project while minimizing its costs^46^. The ‘landscape restoration’ aimed at a minimum of 20% of landscape forest cover, a target that relates to the legal requirements for the Atlantic Forest fixed by the Brazilian Forest Code^88^.

We compared the two scenarios by generating model predictions for the 289 thousand Atlantic Forest fragments remaining in 2016^58^. For each fragment, we compared the ‘current situation’ predictions to the predictions under each restoration scenario, by varying fragment disturbance level and patch/landscape metrics, respectively. Current situation predictions used fragment-specific coordinates to extract spatialized information (e.g. climate, topography), the fragment sizes provided by ref. ^58^ and regional averages for information not available for all fragments (e.g. forest disturbance level, soil type). We obtained these regional averages from the 1819 surveys included in our dataset for each biogeographical region of the Atlantic Forest^69^. All predictions were generated for the same sampling effort (i.e. one hectare), sampling design (i.e. systematic plots dbh ≥5 cm) and using the average number of trees per hectare for each region (Supplementary Table 6).

To generate the predictions of the two restoration scenarios, we used the same covariable values used to predict the ‘current situation’ of biomass, species richness and CWMs with exception to (i) the fragment disturbance level in the ‘fragment restoration’ scenario; and (ii) the patch/landscape metrics in the ‘landscape restoration’ scenario. Because values of fragment disturbance level and landscape metrics were region specific, the changes in disturbance level and landscape metrics between the current and restoration predictions also were region specific. For instance, the decrease in disturbance level and the increase in patch/landscape metrics were smaller for fragments in the Serra do Mar region (lower fragmentation) than in the Alto Paraná region (highest fragmentation, Supplementary Table 6). While generating the predictions for the ‘landscape restoration’ scenario around forest fragments ≥5000 ha, we assumed no increase in fragment size and only half of the forest cover increase.

For the ‘landscape restoration’ scenario, we derived from 100 iterations of 4 × 4 km simulated landscapes the increase in forest cover to reach the 20% target and the corresponding changes in landscape metrics between the ‘current’ and ‘restored’ landscapes. We constructed the simulated landscapes using the averages of patch density and size distribution of forest patches for each biogeographical region. We obtained the distribution of patch size for each simulation from region-specific log-normal distributions fitted to the 2016 Atlantic Forest fragments. For each iteration, we calculated the landscape metrics for the current landscape, and if the landscape forest cover was smaller than 20%, we obtained the amount of forest cover necessary to reach the 20% target. This ‘to-be-restored’ forest area was distributed among the forest patches proportionally to their original sizes, simulating restoration efforts around the existing fragments. We recalculated the landscape metrics for this ‘restored landscape’ and then calculate the changes in these metrics between the ‘current’ and ‘restored’ landscapes. For each biogeographical region, we average these changes for 100 iterations, generated using contributed R package landscapeR^89^. We obtained landscape metrics using the R package SDMTools^90^.

We compared the gains of each restoration scenario for forest biomass, species richness, and CWMs of species properties proportional to the current situation scenario (Fig. 7). We also compared the costs and efficiency of the two scenarios in terms of carbon sequestration (Supplementary Table 6). We assumed an average value of US$750 per hectare for within-fragment restoration, which includes activities to reduce edge effects, control of invasive species, and enrichment plantings^91,92^. We assumed an average of US$1000 per hectare for land opportunity cost (10 years period for cattle-ranching activities)^93^ and an average of US$1500 per hectare for the restoration costs on degraded lands (from seedling plantation to assisted natural regeneration), which varies between US$350 and US$3000 per hectare for the Atlantic Forest^46,93–95^. However, we assumed different restoration costs for each biogeographical region (i.e. less disturbed and fragmented landscapes should cost less to be restored—Supplementary Table 6). As before, we monetarized the expected carbon gains in terms of carbon credits in international markets (US$5 per Mg C paid for projects of forestry and land use^87^). For the ‘landscape restoration’ scenario, we separated carbon gains into those expected inside the remaining fragments and in the restored areas per se (Supplementary Table 6). To calculate the potential of carbon recovery in the restored areas, we used the references of carbon density and the average carbon loss for each biogeographical region (Supplementary Fig. 12), assuming that the drivers of carbon loss in fragments would be similar in restored areas.

### Tree species for the Atlantic Forest restoration

Aiming to assist the selection of species that could maximize restoration efforts (e.g. enriching forest fragments), we provide a list of frequent Atlantic Forest species with higher-than-average potential for carbon storage (i.e. high wood density and maximum adult height), ecological interactions (i.e. large seed mass and late-successional species), and biodiversity conservation (i.e. threat and endemic status). To produce this list, we first selected the species within the 100 most frequent in each of the eight biogeographical regions used in this study (Supplementary Fig. 1). We then ranked species regarding their wood density, maximum adult height, seed mass, ecological group, IUCN threat category, and endemism level. Once again, the three last species categories were treated as ordinal categorical data. We standardized each rank and then average them to obtain a mean rank for each species. In the final list, we included only species with a mean rank higher than the average for all species (frequent or not frequent). We also present the average rank for each group of species properties (i.e. carbon storage potential, ecological interactions, and conservation status) and the frequency of each species in each biogeographical region, as percentages (Supplementary Data 2). Therefore, this list represents a compromise between species that are easier to find for seedling production and have the greatest potential to improve ecosystem provision, taxonomic and functional diversity and/or conservation value of the Atlantic Forest. We removed *Dicksonia sellowiana* from the list because the seedling production from spores for this threatened tree-fern is incipient. It should be stressed that this list does not take into account other species properties that are important for their development in restoration sites, such as drought tolerance or associations with root-symbiotic microbes.

### Reporting summary

Further information on research design is available in the Nature Research Reporting Summary linked to this article.

## Supplementary information

Supplementary Information

Description of Additional Supplementary Files

Supplementary Data 1

Supplementary Data 2

Supplementary Data 3

Reporting Summary

## Data Availability

Survey, species abundances, and trait data used in this study were extracted from the Neotropical Tree Communities database (TreeCo, version 4.0) and are available upon request at http://labtrop.ib.usp.br/doku.php?id=projetos:treeco:start. The list of surveys extracted from the TreeCo database is provided in Supplementary Data 1, together with the corresponding metadata. The sources of survey and species abundance data are referenced in Supplementary Data 1 and sources of species properties data referenced in the Methods or in the Supplementary Data 3. The soil profile database used in this study was accessed at: www.esalq.usp.br/gerd. Other relevant data are available from the corresponding author upon reasonable request. Source data are provided with this paper.

## Source data

Source Data

## Code availability

Codes used to conduct the analysis are available from the corresponding author upon request.
