# Supplementary material for: The erosion of biodiversity and biomass in the Atlantic Forest biodiversity hotspot
Source: Nat Commun. 2020 Dec 11;11:6347. doi: 10.1038/s41467-020-20217-w (PMC7733445; doi:10.1038/s41467-020-20217-w)
Supplement: Supplementary file 1 — Supplementary Information [file 41467_2020_20217_MOESM1_ESM.pdf]

SUPPLEMENTARY INFORMATION

The erosion of biodiversity and biomass in the Atlantic Forest biodiversity hotspot

Renato A. F. de Lima[1,2]*, Alexandre A. Oliveira[1], Gregory R. Pitta[1], André L. de Gasper[3], Alexander C. Vibrans[4], Jérôme Chave[5], Hans ter Steege[2,6] & Paulo I. Prado[1]

[1] Departamento de Ecologia, Instituto de Biociências, Universidade de São Paulo. Rua do Matão, trav. 14, 321, 05508-090, São Paulo, Brazil.

[2] Naturalis Biodiversity Center, Darwinweg 2, 2333 CR Leiden, The Netherlands.

[3] Departamento de Ciências Naturais, Universidade Regional de Blumenau. Rua Antônio da Veiga, 140, 89030-903, Blumenau, Brazil.

[4] Departamento de Engenharia Florestal, Universidade Regional de Blumenau. Rua São Paulo, 3250, 89030-000, Blumenau, Brazil.

[5] Laboratoire Evolution et Diversité Biologique, UMR 5174 CNRS, Université Paul Sabatier, IRD. 118, route de Narbonne, 31062, Toulouse, France.

[6] Systems Ecology, Vrije Universiteit, De Boelelaan 1087, Amsterdam, 1081 HV, Netherlands

*e-mail: raflima@usp.br

This PDF file includes:

1. Supplementary Methods 2
2. Supplementary Tables and Figures 12

1. Supplementary Methods

Forest surveys

We compared the environmental conditions of the 1,819 forest surveys in our sample with the same conditions available for all Atlantic Forest fragments mapped in 2016 by ref. 1. Our sample was representative of the range of climate variation within the Atlantic Forest domain, with a slight sampling bias towards forest fragments in colder and wetter climates, which is partly due to the exclusion of dry deciduous forests surveys (Supplementary Fig. 1, panels A-C). Our sample, however, was strongly biased toward larger forest fragments (Supplementary Fig. 1, panel D), a bias which was taken into consideration before the projection of our results to the entire Atlantic Forest area.

Survey methods

Surveys in the Atlantic Forest typically include tall shrubs, treelets, trees, palms, and tree ferns and they exclude lianas and hemi-epiphytes. Forest descriptors obtained for each survey were tree density, total basal area, and observed species richness. These descriptors were cross-checked for 70% of the surveys for which abundance and biomass per species were available. If descriptors were missing, or their estimation was in doubt, we recomputed them based on phytosociological tables (that provided total tree abundance and richness) or on tree diameter distributions (biomass), as reported in the original studies. We assumed that randomly and systematically distributed sample units have equivalent methodological effects on the estimation of forest biomass and richness[2].

Climate, Topography and Soil information

Based on the verified survey coordinates we extracted the mean annual air temperature, relative humidity, and rainfall, as well as monthly temperature and rainfall at ~100 m resolution[3,4], which were used to compute temperature and rainfall seasonality[5]. We also obtained the climatic water deficit, environmental stress factor (both at ~4.5 km resolution)[6] and the 19 Bioclimatic variables (BIO1, BIO2, …, BIO19) from WorldClim version 2.0 (~1 km resolution)[7]. For 23 surveys from Paraguay and Argentina, sites that are not covered by refs. 3 and 4, temperature and rainfall were obtained from ref. 7. We also extracted mean values of percentage cloud cover (%) and frost day frequency (days, both at ~50 km resolution)[8]. We gathered elevation data from SRTM (~30 m resolution)[9] and used it to calculate slope declivity (range: 0–90 degrees) and slope aspect (1–360 degrees). We combined slope declivity and aspect with latitude to calculate the potential direct incident radiation (MJ cm^{-2} y^{-1})[10].

Based on the same verified coordinates, we obtained missing soil classes for 37% of the surveys from studies conducted at the same sites or from state- or locality-level soil maps, if there was no disagreement across state and national soil maps[11]. For forest types over specific soil classes (*i.e.*, white-sand and swamp forests), missing soil types were

assigned irrespective of possible source conflicts. For each survey, we assigned a soil type classification at the finest taxonomic resolution possible, following the Brazilian classification[12]. To obtain soil properties from soil classes, we used a database with physical and chemical soil properties for ~6000 soil profiles[13]. For each soil type in the database, we averaged the sum of bases ($cmol_c$/kg), cation retention in clay ($cmol_c$/kg) and organic matter content (%) across soil profiles. Moreover, profiles were ranked from 0 (very restrictive) to 4 (very suitable for plant growth) based on soil drainage, depth, chemical fertility, and aluminium toxicity[14]. The sum of these ranks was used as a soil quality measure varying from 0 (very unfit) to 16 (very suitable). Thus, because soil classes in our surveys had the same classification of the soil profile database[12], soil classes were associated with mean values of soil quality and fertility obtained from the soil profile database.

Landscape metrics

We used forest cover maps for the years of 2000 and 2010 (30 m resolution, https://earthenginepartners.appspot.com)[15] to extract 4×4 km landscapes centred on the verified coordinates of each survey. The 2000 map was used for studies between 1995 and 2005 (24% of the surveys) and the 2010 map for studies after 2005 (76% of the surveys). We assumed a scale of effect[16] of 2 km, given that the extent of processes affecting tree populations, such as species dispersal and forest edge effects, generally do not exceed 1–3 km[17–22].

We used a threshold of 70% of canopy closure to classify the 30×30 m pixels of the vegetation maps into forest and non-forest. The 70% threshold was selected based on the evidence of a gap between the 95% confidence intervals of the canopy closure found for open-canopy savannas and Atlantic Forest formations (R.A.F. Lima, unpublished data). Classified maps were used to calculate the proportion of forest cover and core forest cover, the median edge-to-edge distance between fragments and different landscape aggregation indices (*e.g.*, effective mesh size and division, aggregation and splitting indices)[23,24]. Regarding the landscape metrics chosen, the proportion of forest cover and the proportion of core forest cover of the landscape are related to landscape integrity, while the median distance between fragments and landscape aggregation indices are more related to landscape connectivity. The proportion of core forest cover in each classified 4×4 km window was derived using a 90 m threshold (3 times the vegetation map resolution) to delimit fragment edges and cores. The 90 m threshold was chosen because most of the forest edge-effects are concentrated in distances up to ~100 m[17,25–27].

Because of problems related to the distinction between natural and planted forests in the maps provided by ref. 15[28], we used a Tree Plantation map[29] to delineate forest plantations. To minimize differences in landscape metrics between the year of the map and that of the forest survey, we only considered surveys conducted after the decline and

levelling off in the Atlantic Forest deforestation rates[1]. This decline occurred after 1990 for Rio de Janeiro and Rio Grande do Sul states. For the states of Bahia, Goiás, and Mato Grosso do Sul deforestation rates remained high until 2000[1]. For the other countries and Brazilian states, we set 1995 as the cut-off year. Surveys conducted prior to the cut-off year but inside large (>1000 ha) conservation units or university campuses were kept in the dataset. We excluded surveys in fragments that were suppressed afterwards. Landscape metrics were extracted from maps using R[30] and the contributed packages raster[31], rgdal[32], rgeos[33], sp[34], maptools[35], fasterize[36], cleangeo[37] and SDMTools[24].

Forest fragment size

We assigned a forest fragment to each survey and obtained their size based on information from the original publications. Fragment size was cross-validated using other sources of information. We used vegetation maps with 30 m resolution[19], so forest fragments should be detectable down to a resolution of ca. 0.1 ha. Less than 1% of our surveys were in fragments <0.9 ha (10 times the resolution of the maps). Therefore, cross-validation, as well as obtaining missing fragment sizes, was performed with good confidence and accuracy. The smallest fragment was 0.2 ha (Supplementary Table 1), while the few large fragments (>50,000 ha) were concentrated in the Serra do Mar and *Araucaria* eco-regions of São Paulo, Paraná, and Santa Catarina states. Fragment size is a good proxy for the habitat availability for species establishment and proportion of the fragment core area susceptible to landscape/edge effects[38]. We could not obtain an accurate (mean) distance between the sampling unit and forest edges, because this information was generally missing from the original publication. Moreover, about 60% of the surveys are aggregates of multiple plots scattered across the fragment, but the coordinates of each plot were not provided. For older surveys, in particular, we had no precise coordinates within the sampled fragment, and the centre of the fragment was then taken as the most likely coordinate (18% of the surveys). Finally, typographical errors in reporting the geographical coordinates or conversion errors also caused inconsistencies that could not be solved below the fragment scale.

Forest disturbance level

We classified the surveys into three levels of forest disturbance: high, medium and low. We assumed the following scores to each level, which can be interpreted as the time in years since the last disturbance: high= 40, medium= 70 and low=100. This ordered classification has support in the legal classification of the Atlantic Forest[39] and it implicitly assumes that highly disturbed forests can redevelop into medium and low disturbance forests through natural regeneration or restoration. It also assumes that low-disturbance forests can regress into medium or high disturbance levels depending on the type and intensity of disturbances[40–42]. Natural events can alter the structure and dynamics of forest fragments[17,43], but we assumed that these events were rare[44] and that

the major drivers of forest disturbance are related to direct (*e.g.*, clear-cutting, logging, and fire) and indirect human activities (*e.g.*, invasive exotic species). If there were doubts about the classification of the forest disturbance level based on the information provided by the original author of the publication (about 10% of the cases), the density of typical pioneer Neotropical genera was used to assign the surveys to one of the three forest disturbance levels. We discarded a total of 136 surveys for which a forest disturbance level could not be unambiguously assigned. We were unable to refine this classification any further due to a lack of more objective and detailed information in the original publications. For most surveys, the only information provided was a simple mention of the forest successional stage (*e.g.*, initial, intermediary or advanced).

Processing and selection of variables

Some of the response variables had left-skewed distributions, and they were transformed accordingly to meet the normality of the regression residuals. We used logarithmic and power transformations, using a Box-Cox procedure to choose between both transformations and to select the best exponent in the case of power transformations. We eventually transformed biomass, richness, and seed mass. We log-transformed some of the dependent variables, namely tree abundance, fragment size, slope declivity, soil fertility, which also had left-skewed distributions. We power-transformed frost frequency, air relative humidity, and the climatic water deficit. Before analysis, we standardized all numerical explanatory variables [(observed − mean)/standard deviation], so that all variables had a similar range of variation, making their estimated effects comparable.

All categorical variables, namely ecological groups, extinction and endemism levels, and forest disturbance level, were treated as ordinal categorical data (also known as rank-ordered data) since they represent ordered and directional changes in the forest. For these variables, we compared the impact of different scoring methods: naïve (described above for each variable), ridit, normal and log-normal scores[45,46]. We compared the performance of models fitted using these different scoring methods based on the assumptions of normality of the studentized residuals (Shapiro-Wilk test), homoscedasticity (Breusch-Pagan test) and the number of outliers (Bonferroni test). For disturbance level, the only explanatory categorical variable we had, we also compared the difference in the Akaike information criterion (AIC) of the models fitted including the different scoring methods. The use of the ridit scores resulted in models that had marginally or significantly better performance than other scoring methods. Therefore, ridit scores were used throughout the analysis for all categorical variables. Ridit scores were obtained using the contributed R package ridittools[47].

The selection of candidate explanatory variables to be included in the regression analysis was based on their performance in previous similar studies and on the absence of strong correlation (Pearson $r > 0.75$) or co-linearity with other candidate explanatory

variables. We included mean annual temperature and rainfall, regarded here as the two basic climate variables, and different measures of their seasonality. We also included the climatic water deficit, environmental stress factor and the 19 bioclimatic variables. The measure of temperature seasonality with the smallest correlation and no co-linearity with annual temperature was mean diurnal range (BIO2), which presented a strong correlation with temperature annual range (BIO7 - Supplementary Fig. 2). The climatic water deficit was always chosen as the best rainfall seasonality variable and it presented a strong correlation with all other rainfall seasonality measures available, including the Precipitation of Driest Quarter (BIO17), the number of dry months (rainfall below 60 mm) and the rainfall seasonality index[5] (Supplementary Fig. 3). With few exceptions, the correlations among mean soil properties tended to be smaller than for other groups of environmental variables (Supplementary Fig. 4; see Methods section for details on soil properties).

Although we estimated several landscape metrics, the metrics with the lowest co-linearity with forest cover were landscape shape index and median fragment distance (Supplementary Fig. 5), which provide simple measures of patch aggregation in the landscape[23]. Values of the landscape shape index close to one represent high aggregation, while higher values representing disaggregation of patches. Similarly, low values of median fragment distance represent a higher aggregation of patches. Altitude, latitude, and distance from the ocean (our proxy of site continentality) explained almost all variation in annual temperature (adjusted R^2= 92.3%; F[d.o.f. =1817] = 5472), so they were not considered as candidate variables during analysis (Supplementary Fig. 6). The regression models containing all the pre-selected candidate natural and human-related variables were tested for co-linearity and all condition indices were below 4 – considered to be quite low[48]. There was some co-linearity between temperature and climatic water deficit and between forest cover and fragment size, but the correlation between these variables in the final models was acceptable (Pearson's $r \leq 0.75$).

Assessing the precision of model predictions

To assess the precision of our model predictions under the human-free scenario, we generated 5,000 simulations by sampling values of the coefficients estimated for the fixed and random effects of the model. These values were sampled from a Gaussian distribution with the estimated mean and standard errors of the coefficients. For each run, we re-calculated the model predictions but using draws of model coefficients. We then re-calculated two metrics presented in the main text, namely the standardized index of loss and the proportion of surveys with negative standardized indices of loss. This analysis was performed separately for each variable (*i.e.*, forest biomass, species richness, and species properties) using R[30] and the contributed package merTools[49], using the function 'predictInterval'.

We found for all variables that the bootstrapped prediction intervals included the observed standardized indices of loss and these intervals generally did not include zero (Supplementary Table 3). The estimates of the bootstrap standard deviation were significantly higher than the observed ones for all variables (results not shown). Bias in the variance estimation is common for this type of simulation[50] and is a probable explanation for the tendency of simulated indices to be slightly more conservative than the observed ones.

For the CWM of species extinction level, the prediction interval for the index of loss included zero, meaning that the predictions for this variable were the least precise. But, the proportion of surveys with negative proportions was within the simulated intervals, meaning that the lower precision of the predictions for the index of loss of this trait is related to extreme positive values of the index. Further inspection of the index of loss for the extinction level revealed that these extreme values were commonly related to surveys with a high relative abundance of the vulnerable *Euterpe edulis* (24 to 51%) and of the endangered *Tabebuia cassinoides* (57 to 81%), two species that are red-listed due to overexploitation. For these sites, the models generated predictions 79 to 89% lower than the observed ones, generating such extreme positive indices. But those cases were rare (~15 surveys). A similar situation was found for maximum height, but in this case, the inclusion of zero within the prediction interval is probably more related to a lack of evidence for human-related impacts on this specific trait than a lack of certainty around the observed means. The high density of some species also caused some extreme values for seed mass (*e.g.*, *Araucaria angustifolia* which has a mean seed mass of 6.3 g), however, such cases had a lower impact on the predictions for this trait.

Basal area as a measure of forest biomass

Forest above ground biomass (AGB; live oven-dry matter of trees, in Mg ha^{-1}) is a key determinant of ecosystem integrity. However, it is more difficult to retrieve estimates of AGB than basal area (BA; summed cross-section area of the tree trunks, in m^2 ha^{-1}) from the literature. Estimates of AGB[6] in forest plots are expected to be closely related to BA because both depend on tree diameter. Indeed, a strong statistical relationship between BA and AGB has been found in Mexican forests at stand level and the Atlantic Forest at species level[51]. But the strength of such a relationship at stand level has never been tested for the Atlantic Forest.

We compiled 503 surveys for which both AGB and BA were available from two Brazilian state forest inventories[52–55]. AGB estimates represent different Atlantic Forest formations, using allometric equations specific to each forest type, based on tree diameter and height (Supplementary Table 6). We fitted simple linear regression models to the log-transformed values of AGB and BA for each set of data separately. These models were compared against non-linear models (*i.e.*, power and exponential functions) to assess the assumption of linearity in this relationship. We also tested the impact of the survey

sampling area in the relationship between the two variables by adding it as weights in the regression model and inspecting if there was an improvement in model fit.

We found a strong, linear and positive relationship of the log-transformed values of AGB and BA for each of the main Atlantic Forest types. Basal area explained 89–97% of the variation in AGB (Supplementary Fig. 9). For Seasonal forests (Supplementary Fig. 9, panel C), a power-function fitted values slightly better (results not shown) due to a better fit of very extreme values of BA (below 8 and above 40 $m^2\ ha^{-1}$ of BA). But the linear function was a very good description of the relationship for all forest types.

We also fitted linear regressions for forest types altogether. The linear mixed-effects regression model assuming different random intercepts and slopes for each forest type (conditional R^2= 96.5%) yielded a much better fit than the model considering only BA as a predictor of AGB (adjusted-R^2= 88.2%). So, the general expression averaging parameter estimates across forest types is:

$$AGB = \exp[1.129 + 1.178 \ln(BA)]$$

The 95% confidence interval estimates of the regression parameters were 0.598–1.688 for the intercept and 1.031–1.327 for the slope parameter. We validated this equation with another set of 53 unpublished Atlantic Forest surveys using the global allometric equations provided by ref. 56, and found an excellent match, demonstrating that AGB can be confidently inferred from BA. We converted aboveground biomass (Mg ha^{-1}) into carbon storage (Mg C ha^{-1}) by assuming 47% of carbon concentration[57].

Supplementary References

1. Fundação SOS Mata Atlântica & Instituto Nacional de Pesquisas Espaciais (INPE). *Atlas dos remanescentes florestais da Mata Atlântica: período 2016-2017*. Fundação SOS Mata Atlântica (2018).
2. Cochran, W. G. *Sampling techniques*. John Wiley & Sons, New York (1977).
3. Alvares, C. A., Stape, J. L., Sentelhas, P. C. & Gonçalves, J. L. de M. Modeling monthly mean air temperature for Brazil. *Theor. Appl. Climatol.* **113**, 407–427 (2013).
4. Alvares, C. A., de Mattos, E. M., Sentelhas, P. C., Miranda, A. C. & Stape, J. L. Modeling temporal and spatial variability of leaf wetness duration in Brazil. *Theor. Appl. Climatol.* **120**, 455–467 (2015).
5. Walsh, R. P. D. & Lawler, D. M. Rainfall seasonality: description, spatial patterns and change through time. *Weather* **36**, 201–208 (1981).
6. Chave, J. *et al.* Improved allometric models to estimate the aboveground biomass of tropical trees. *Glob. Chang. Biol.* **20**, 3177–3190 (2014).
7. Fick, S. E. & Hijmans, R. J. WorldClim 2: new 1-km spatial resolution climate surfaces for global land areas. *Int. J. Climatol.* **37**, 4302–4315 (2017).
8. Harris, I., Jones, P. D., Osborn, T. J. & Lister, D. H. Updated high-resolution grids of monthly climatic observations - the CRU TS3.10 Dataset. *Int. J. Climatol.* **34**, 623–642 (2014).
9. Farr, T. G. *et al.* The Shuttle Radar Topography Mission. *Rev. Geophys.* **45**, RG2004 (2007).

10. McCune, B. & Keon, D. Equations for potential annual direct incident radiation and heat load. *J. Veg. Sci.* **13**, 603–606 (2002).
11. Santos, H. G. dos *et al. O novo mapa de solos do Brasil: legenda atualizada*. Embrapa Solos, Rio de Janeiro (2011).
12. Santos, H. G. *et al. Sistema brasileiro de classificação de Solos*. Embrapa, Brasília, 4th ed. (2014).
13. Benedetti, M. M., Curi, N., Sparovek, G., Carvalho Filho, A. de & Silva, S. H. G. Updated Brazilian's Georeferenced Soil Database – An Improvement for International Scientific Information Exchanging. in *Principles, Application and Assessment in Soil Science* (ed. Gungor, B. E. O.) InTech, Rijeka, Croatia. pp. 309–332 (2011).
14. Pereira, L. C. & Lombarde Neto, F. *Avaliação da aptidão agrícola das terras: proposta metodológica*. Embrapa Meio Ambiente. Documentos, 43 (2004).
15. Hansen, M. C. *et al.* High-resolution global maps of 21st-century forest cover change. *Science* **342**, 850–853 (2013).
16. Jackson, H. B. & Fahrig, L. What size is a biologically relevant landscape? *Landsc. Ecol.* **27**, 929–941 (2012).
17. Laurance, W. F. et al. The fate of Amazonian forest fragments: A 32-year investigation. Biol. Conserv. 144, 56–67 (2011).
18. Holbrook, K. K. M. & Smith, T. T. B. Seed dispersal and movement patterns in two species of Ceratogymna hornbills in a West African tropical lowland forest. *Oecologia* **125**, 249–257 (2000).
19. Nathan, R., Horn, H. S., Chave, J. & Levin, S. A. Mechanistic models for tree seed dispersal by wind in dense forests and open landscapes. in *Seed dispersal and frugivory: ecology, evolution and conservation* (eds. Levey, D. J., Silva, W. R. & Galetti, M.) CABI International, pp. 69–82 (2002).
20. Thomson, F. J., Moles, A. T., Auld, T. D. & Kingsford, R. T. Seed dispersal distance is more strongly correlated with plant height than with seed mass. *J. Ecol.* **99**, 1299–1307 (2011).
21. Clark *et al.* Seed dispersal near and Far: patterns across temperate and tropical forests. *Ecology* **80**, 1475–1494 (1999).
22. Clark, C. J., Poulsen, J. R., Bolker, B. M., Connor, E. F. & Parker, V. T. Comparative seed shadows of bird-, monkey-, and wind-dispersed trees. *Ecology* **86**, 2684–2694 (2005).
23. McGarigal, K., Cushman, S. & Ene, E. FRAGSTATS v4: Spatial Pattern Analysis Program for Categorical and Continuous Maps. University of Massachusetts, Amherst (2012).
24. VanDerWal, J., Falconi, L., Januchowski, S., Shoo, L. & Collin Storlie. SDMTools: Species Distribution Modelling Tools: Tools for processing data associated with species distribution modelling exercises. R package version 1.1-221 (2014).
25. Paula, M. D., Costa, C. P. A. & Tabarelli, M. Carbon storage in a fragmented landscape of Atlantic Forest: the role played by edge-affected habitats and emergent trees. *Trop. Conserv. Sci.* **4**, 349–358 (2011).
26. Broadbent, E. N. *et al.* Forest fragmentation and edge effects from deforestation and selective logging in the Brazilian Amazon. *Biol. Conserv.* **141**, 1745–1757 (2008).

27. Chaplin-Kramer, R. *et al.* Degradation in carbon stocks near tropical forest edges. *Nat. Commun.* **6**, 10158 (2015).
28. Tropek, R. *et al.* Comment on 'High-resolution global maps of 21st-century forest cover change'. *Science* **344**, 981–981 (2013).
29. Petersen, R. *et al. Mapping Tree Plantations with Multispectral Imagery: Preliminary Results for Seven Tropical Countries*. Washington, DC. (2016), available at www.wri.org/publication/mapping-treeplantations.
30. R Core Team. R: A language and environment for statistical computing. R Foundation for Statistical Computing, Vienna (2018).
31. Hijmans, R. raster: Geographic data analysis and modeling. R package version 2.5-8 (2016).
32. Bivand, R., Keitt, T. & Rowlingson, B. rgdal: Bindings for the 'Geospatial' Data Abstraction Library. R Package version 1.3-6 (2018).
33. Bivand, R. & Rundel, C. rgeos: Interface to Geometry Engine - Open Source ('GEOS'). R package version 0.4.2 (2018).
34. Pebesma, E. J. & Bivand, R. S. Classes and methods for spatial data in R. R News **5**, 9–13 (2005).
35. Bivand, R. & Lewin-Koh, N. maptools: Tools for handling spatial objects. R Package version 0.9-4 (2018).
36. Ross, N. fasterize: Fast polygon to raster conversion. R package version 1.0 (2018).
37. Blondel, E. cleangeo: Cleaning geometries from spatial objects. R package version 0.2-2 (2017).
38. Pütz, S. *et al.* Long-term carbon loss in fragmented Neotropical forests. *Nat. Commun.* **5**, 5037 (2014).
39. Brasil. *Resolução Conama nº 10. Diário Oficial da União* **209**(1): 16497-16498. (1993).
40. Brown, S. & Lugo, A. E. Tropical Secondary Forests. *J. Trop. Ecol.* **6**, 1–32 (1990).
41. Malhi, Y., Gardner, T. A., Goldsmith, G. R., Silman, M. R. & Zelazowski, P. Tropical Forests in the Anthropocene. *Annu. Rev. Environ. Resour.* **39**, 125–159 (2014).
42. Joly, C. A., Metzger, J. P. & Tabarelli, M. Experiences from the Brazilian Atlantic Forest: ecological findings and conservation initiatives. *New Phytol.* **204**, 459–473 (2014).
43. Lima, R. A. F., Rother, D. C., Muler, A. E., Lepsch, I. F. & Rodrigues, R. R. Bamboo overabundance alters forest structure and dynamics in the Atlantic Forest hotspot. *Biol. Conserv.* **147**, 32–39 (2012).
44. Pezza, A. B. & Simmonds, I. The first South Atlantic hurricane: Unprecedented blocking, low shear and climate change. *Geophys. Res. Lett.* **32**, L15712 (2005).
45. Golden, L. L. & Brockett, P. L. The effect of alternative scoring methods on the analysis of rank order categorical data. *J. Math. Sociol.* **12**, 383–414 (1987).
46. Chen, H.-C. & Wang, N.-S. The Assignment of Scores Procedure for Ordinal Categorical Data. *Sci. World J.* **2014**, 1–7 (2014).
47. Bohlman, E. ridittools: Useful functions for ridit analysis. R package version 0.1 (2018).
48. Belsley, D. A. A Guide to using the collinearity diagnostics. *Comput. Sci. Econ. Manag.* **4**, 33–50 (1991).

49. Knowles, J. E. & Frederick, C. merTools: Tools for analyzing mixed effect regression models. R package version 0.4.1 (2018).
50. Kitagawa, G. & Konishi, S. Bias and variance reduction techniques for bootstrap information criteria. *Ann. Inst. Stat. Math.* **62**, 209–234 (2010).
51. Bello, C. *et al.* Defaunation affects carbon storage in tropical forests. *Sci. Adv.* **1**, e1501105 (2015).
52. Scolforo, J. R., Mello, J. M., Oliveira, A. D., Pereira, R. M. & Guedes, I. C. L. Volumetria, peso de matéria seca e carbono para o domínio atlântico em Minas Gerais. in *Inventário Florestal de Minas Gerais: Floresta Estacional Semidecidual e Ombrófila - Florística, Estrutura, Similaridade, Distribuição Diamétrica e de Altura, Volumetria, Tendências de Crescimento e Manejo Florestal* (eds. Scolforo, J. R., Mello, J. M. & Silva, C. P. C.) Universidade Federal de Lavras, pp. 461–630 (2008).
53. Vibrans, A. C. *et al.* Amostragem dos remanescentes da Floresta Estacional Decidual em Santa Catarina. in *Inventário Florístico Florestal de Santa Catarina: Floresta Ombrófila Decidual (vol. II)* (eds. Vibrans, A. C., Sevegnani, L., Gasper, A. L. & Lingner, D. V.) Edifurb, pp. 33–77 (2012).
54. Vibrans, A. C. *et al.* Amostragem dos remanescentes florestais da Floresta Ombrófila Mista em Santa Catarina. in *Inventário Florístico Florestal de Santa Catarina: Floresta Ombrófila Mista (vol. III)* (eds. Vibrans, A. C., Sevegnani, L., Gasper, A. L. & Lingner, D. V.) Edifurb, pp. 33–93 (2013).
55. Vibrans, A. C. *et al.* Amostragem dos remanescentes florestais da Floresta Ombrófila Densa em Santa Catarina. in *Inventário Florístico Florestal de Santa Catarina: Floresta Ombrófila Densa (vol. IV)* (eds. Vibrans, A. C., Sevegnani, L., Gasper, A. L. & Lingner, D. V.) Edifurb, pp. 33–95 (2013).
56. Chave, J. *et al.* Tree allometry and improved estimation of carbon stocks and balance in tropical forests. *Oecologia* **145**, 87–99 (2005).
57. Thomas, S. C. & Martin, A. R. Carbon content of tree tissues: A synthesis. *Forests* **3**, 332–352 (2012).

2. Supplementary Tables and Figures

Supplementary Table 1. Summary of the environmental and human-related conditions of the forest fragments studied.

Mean, standard deviation (s.d.), minimum (Min.), maximum (Max.), first and third quartiles, and the coefficient of variation (%) of t continuous descriptors of the surveyed sites (*n*= 1819). Temperature, rainfall, climatic water deficit, mean frost, and potential direct incident radiation are annual means, while soil quality and mean shape index are dimensionless. For climatic water deficit, mean frost frequency, slope declivity, fragment size and core forest cover, all with skewed distributions, values are actually medians and the coefficient of variation was calculated for the transformed variables, as used in the data analysis.

Variable	Mean ± s.d.	Min.-Max.	1st - 3rd quartiles	Coefficient of variation
Annual temperature (^oC)	19.3 ± 2.8	11.3−25.7	17.2−21.3	14.5
Diurnal range (^oC)	10.4 ± 1.65	5.7−14.5	9.5−11.7	15.8
Frost frequency (days)	0.020 ± 0.18	0−1.032	0−0.126	71.9
Annual rainfall (mm)	1637 ± 301	501−3062	1406−1811	18.4
Climatic water deficit (mm)	-136 ± 173	-1091−0	-256−0	95.9
Relative humidity (%)	79.4 ± 3.3	63.8−84.1	77.1−81.0	34.5
Cloud cover (%)	69.2 ± 5.4	47.3−78.9	65.5−73.8	7.8
Direct radiation (MJ cm^{-2} yr^{-1})	0.97 ± 0.12	0.44−1.17	0.91−1.05	12.2
Slope declivity (^{o})	10.0 ± 8.9	0−47	4−18	35.4
Soil quality	6.99 ± 2.19	1.9−14.5	6−8	31.7
Cation retention ($cmol_c$ kg^{-1})	14.6 ± 14.2	2.9−81.0	9.6−22.6	20.4
Fragment size (ha)	155 ± 42473	0.2−303,000	29−1784	53.5
Forest cover (%)	47.6 ± 29.5	0.04−100	23.1−71.7	62.0
Core forest cover (%)	13.9 ± 30.2	0−100	3.87−46.6	43.1
Mean shape index	1.56 ± 0.29	1−4.41	1.42−1.63	18.8
Med. fragment distance (m)	1889 ± 736	0−3047	1364−2121	45.2

Supplementary Table 2. Description of the linear mixed-effects regression models with the best fit to forest biomass, species richness, and species properties data.

Each model is the result of a model selection procedure of different random and fixed effects structures. Candidate fixed effects included different environmental and human explanatory variables (and their interactions), while random effects included the survey methodology and the Atlantic Forest biogeographical regions. For each model, we present the transformation applied to each variable, the number of observation (*n*), marginal/conditional R^2, full model Chi-squared statistics (χ^2) and number of model parameters (Par.), along with the selected fixed effects and interactions (separated by a vertical slash). Full variables names are given in Supplementary Table 1 (CWD= Climatic water deficit). All models have *p*-value < 2.2e-16, except for Adult height (*p*-value = 2.54e-13).

**Variable**	**Transf.**	***n***	**R^2 (%)**	**χ^2**	**Par.**	**Fixed effects**	**Interactions**
Forest biomass	log	1676	38.5/52.7	362.9	16	Temperature|Slope|Soil fertility|For. disturbance|Core for. cover|Tree density|Effort	Temperature:Slope|For. disturbance:Tree density|For. disturbance:Core forest cover
Species richness	log	1790	58.2/70.7	390.2	20	Temperature|Diurnal range|Slope|Env. stress|Soil fertility|Organic matter|For. disturbance|Fragment size|Forest cover|Shape index|log(N)	Env. stress:Soil fertility
Wood density	log	1213	18.9/25.8	126.6	15	Temp.seasonality|Slope|Cloud cover|Soil quality|Radiation|For. disturbance|Forest cover|Fragment size|Frag. distance|Effort	Radiation:For. disturbance
Adult height	–	1213	9.4/27.0	82.2	14	Temperature|Diurnal range|CWD|Cloud cover|Soil fertility|Slope|Fragment size|Forest cover|Effort	Fragment size:Forest cover
Seed mass	log	1214	28.3/35.2	203.2	14	Temperature|Isothermality|Radiation|CWD|Humidity|Soil quality|For. disturbance|Core for. cover|Effort	Humidity:Soil quality
Ecological group	Ridit	1214	23.5/35.7	211.2	15	Frost|Diurnal range|Humidity|Soil fertility|For. disturbance|Core for. cover|Tree density|Effort	Frost:Humidity|Humidity:Soil fertility|For.disturbance:Core for. cover
Extinction level	Ridit	1214	26.2/41.6	186.3	13	Frost|Diurnal range|CWD|Cloud cover|Organic matter|For. disturbance|Core for. cover|Effort	Frost:CWD
Endemism level	Ridit	1213	37.1/43.6	290.9	16	Temperature|Slope|Humidity|Cloud cover|Soil quality|For. disturbance|Fragment size|Shape index|Effort	Temperature:Humidity|Humidity:Soil quality|For. disturbance:Fragment size

Supplementary Table 3. Summary statistics for the fixed effects of the linear mixed-effects regression models.

For each of the variables describe here, we present the parameter estimates and standard errors associated with each fixed effect in the model. We also present the results of the Analysis of Deviance, which includes the type II Wald Chi-square test (χ^2) and its *p*-value. Full variables names and units are given in Supplementary Table 1 (CWD= Climatic water deficit).

**Variable**	**Fixed effects**	**Estimate**	**Std. error**	**χ^2**	***p*-value**
Forest biomass	Temperature	-0.043	0.012	8.57	0.0034
	Slope	0.019	0.008	10.49	0.0012
	Soil fertility	-0.010	0.008	1.48	0.2238
	For. disturbance	0.137	0.008	273.18	0.0000
	Core for. cover	0.033	0.010	13.81	0.0002
	Tree density	0.193	0.014	224.80	0.0000
	Effort	-0.007	0.024	0.09	0.7658
	Temperature:Slope	0.044	0.008	27.01	0.0000
	For. disturbance:Core For. cover	0.025	0.008	10.73	0.0011
	For. disturbance:Tree density	-0.039	0.008	23.10	0.0000
Species richness	Temperature	0.109	0.034	10.24	0.0014
	Diurnal range	0.045	0.014	10.60	0.0011
	Slope	0.079	0.010	70.09	0.0000
	Env. stress	-0.062	0.014	19.79	0.0000
	Soil fertility	-0.086	0.009	74.89	0.0000
	Organic matter	-0.068	0.009	53.58	0.0000
	For. disturbance	0.049	0.009	29.37	0.0000
	Fragment size	0.026	0.013	3.90	0.0483
	Forest cover	0.044	0.015	8.29	0.0040
	Shape index	-0.035	0.009	16.75	0.0000
	log(N)	0.382	0.030	162.39	0.0000
	Env. stress:Soil fertility	0.032	0.010	10.91	0.0010
Wood density	Temp. Seasonality	-0.015	0.004	15.65	0.0001
	Slope	-0.005	0.003	3.90	0.0483
	Cloud cover	-0.020	0.003	47.23	0.0000
	Soil quality	0.011	0.003	16.43	0.0001
	Radiation	0.001	0.003	0.47	0.4942
	For. disturbance	0.004	0.002	2.96	0.0853
	Forest cover	-0.009	0.004	5.52	0.0188
	Fragment size	0.008	0.003	5.58	0.0182
	Frag. distance	-0.006	0.003	5.69	0.0171
	Effort	0.008	0.003	11.32	0.0008

	Radiation:For. disturbance	-0.007	0.002	8.52	0.0035
Adult height	Temperature	0.564	0.117	23.10	0.0000
	Diurnal range	0.401	0.115	12.23	0.0005
	CWD	0.951	0.153	38.85	0.0000
	Cloud cover	0.177	0.095	3.49	0.0617
	Soil fertility	-0.342	0.077	19.66	0.0000
	Slope	0.141	0.078	3.30	0.0693
	Fragment size	0.148	0.117	0.02	0.8746
	Forest cover	-0.020	0.126	0.01	0.9035
	Effort	0.222	0.082	7.31	0.0069
	Fragment size:Forest cover	-0.240	0.082	8.56	0.0034
Seed mass	Temperature	0.305	0.027	132.27	0.0000
	Isothermality	0.218	0.034	41.91	0.0000
	Radiation	0.036	0.019	3.70	0.0545
	CWD	0.183	0.041	20.22	0.0000
	Humidity	0.140	0.033	20.47	0.0000
	Soil quality	0.059	0.021	0.64	0.4233
	For. disturbance	0.091	0.018	24.51	0.0000
	Core for. cover	0.029	0.022	1.77	0.1836
	Effort	0.100	0.019	27.33	0.0000
	Humidity:Soil quality	0.124	0.020	40.67	0.0000
Ecological group	Frost	-0.001	0.012	0.05	0.8201
	Diurnal range	0.015	0.014	1.09	0.2959
	Humidity	0.033	0.017	0.40	0.5253
	Soil fertility	-0.028	0.009	4.61	0.0319
	For. disturbance	0.081	0.008	94.25	0.0000
	Core for. cover	0.063	0.010	43.16	0.0000
	Tree density	0.060	0.011	27.70	0.0000
	Effort	0.030	0.009	12.21	0.0005
	Frost:Humidity	0.035	0.011	10.57	0.0011
	Humidity:Soil fertility	-0.038	0.008	20.63	0.0000
	For. disturbance:Core for. cover	0.028	0.008	12.86	0.0003
Extinction level	Frost	0.022	0.008	7.36	0.0067
	Diurnal range	0.017	0.008	4.59	0.0322
	CWD	0.058	0.011	22.24	0.0000
	Cloud cover	0.022	0.006	12.65	0.0004
	Organic matter	0.034	0.005	43.72	0.0000
	For. disturbance	0.015	0.005	9.11	0.0025
	Core for. cover	0.022	0.006	12.38	0.0004
	Effort	0.026	0.005	25.25	0.0000
	Frost:CWD	0.019	0.007	7.60	0.0058

Supplementary Table 4. Precision of the model predictions in the human-free scenario.

For each variable included in our study we present the observed, the bootstrap estimates and 95% confidence intervals (inside brackets) of the average standardized index of loss and the frequency of Atlantic Forest surveys presenting losses due to human-related impacts (*i.e.*, negative standardized index of loss). The bootstrap means and intervals of the predictions were generated using 5000 samples of the model coefficients taken from normal distributions around the parameters estimated by the models fitted to the data.

**Variable**	**Standardized Index of loss**		**Frequency (%)**	
	Observed	Simulated [CI 95%]	Observed	Simulated [CI 95%]
Forest biomass	-0.089	-0.082 [-0.115; -0.047]	83.5	74.9 [65.3; 83.4]
Species richness	-0.072	-0.065 [-0.103; -0.022]	82.7	71.0 [57.7; 82.3]
Wood density	-0.066	-0.099 [-0.163; -0.042]	59.9	61.3 [52.7; 69.5]
Adult height	0.037	0.050 [-0.003; 0.106]	38.0	40.5 [28.3; 53.9]
Seed mass	-0.101	-0.079 [-0.126; -0.028]	75.8	68.0 [59.1; 76.3]
Ecological group	-0.182	-0.152 [-0.213; -0.085]	84.9	74.6 [65.7; 82.3]
Extinction level	-0.098	-0.022 [-0.172; 0.248]	66.9	59.5 [48.4; 69.9]
Endemism level	-0.267	-0.251 [-0.321; -0.075]	86.9	79.5 [71.1; 86.6]

Supplementary Table 5. Mean reference values of forest biomass, tree species richness and species properties for the Atlantic Forest.

Average estimates were obtained using only surveys conducted in low-disturbance forest fragments. For biomass, we considered only surveys with total sampling area >0.2 ha. For species richness, we considered only surveys of about one hectare of total effort. References for the community-weighted means for species properties were obtained only for surveys using dbh ≥5 cm and with a minimum sample size of 500 individuals. Values of average carbon were estimated based on the relationship between above ground biomass and basal area for Atlantic Forest sites (see 'Supplementary Methods') and assuming 47% of carbon content in dry biomass. Mean references and confidence intervals were estimated separately for each biogeographical region, but here we present the weighted average of these estimates across these regions, using the region areas as weights.

**Forest descriptor**	**Dbh cutoff (cm)**	**Number of surveys**	**Mean reference**	**Confidence interval (95%)**
Basal area (m^2 ha^{-1})	≥5.0	239	33.6	29.3−37.9
	≥10.0	82	30.9	22.8−39.1
Carbon storage (Mg ha^{-1})	≥5.0	239	93.0	79.3−107.2
	≥10.0	82	84.3	59.5−111.3
Species richness (ha^{-1})	≥5.0	121	104	84−130
	≥10.0	37	75	51−115
Species properties				
Wood density (g cm^{-3})	≥5.0	326	0.632	0.617–0.645
Max. adult height (m)	≥5.0	326	22.5	21.7–23.3
Seed mass (g)	≥5.0	326	0.172	0.132–0.198
Ecological group	≥5.0	326	0.912	0.848–0.975
Extinction level	≥5.0	326	0.214	0.158–0.306
Endemism level	≥5.0	326	0.646	0.575–0.712

Supplementary Table 6. Reference values used to simulate the two restoration scenarios, 'fragment restoration' (Scenario 1) and 'landscape restoration' (Scenario 2), and the description of the gains and costs considered to calculate their outcomes.

For each biogeographical region of the Atlantic Forests, we provide the average values used to predicted the differences between the 'current' and the two restoration scenarios. For Scenario 1, there is no increase in forest cover (FC). For Scenario 2, the increase in FC represents the amount of forest cover that should be restored to reach 20% of average landscape forest cover. Costs of restoration for the Scenarios 1 and 2 vary according to the average disturbance level of the fragments and the average forest cover, respectively.

	**Alto Paraná**	**Araucaria forests**	**Atlantic Dry**	**Bahia Coast**	**Bahia Interior**	**Northeast forests**	**Serra do Mar**	**Uruguay forests**
Tree density (dbh≥ 5 cm ha^{-1})	1400	1718	891	1533	1505	1263.8	1663.8	1681.1
Ref. carbon storage (Mg C ha^{-1})	80.1	115.6	45.2	89.1	67.6	77.4	96.4	90
Fragment disturbance level[a]	-0.19	-0.06	0.16	0.16	-0.14	-0.4	0.22	0.11
Average forest cover (FC) (%)	8.6	17.8	23.6	14.4	11.2	17.0	41.6	16.5
Average forest size (ha)	52	67	217	51	43	61	163	68
Mean patch density (16 km^{-2})	5.7	6.8	4.8	5.5	7.5	7.1	5.2	5.3
Med. fragment distance (km)	1.79	1.78	2.00	1.47	2.00	2.05	1.23	1.73
Remaining FC (million ha)	3.46	3.84	2.81	1.63	2.77	1.02	4.69	0.93
FC increase (Scenario 2; million ha)	1.80	1.54	1.43	1.88	1.75	1.34	1.17	1.86
Carbon Gain Scenario 1 (Mg ha^{-1})	5.1	5.5	2.6	3.5	4.2	6.5	3.3	3.7
Carbon Gain Scenario 2 (Mg ha^{-1})	59.7	95.1	33.6	63.5	48.7	48.0	74.1	69.6
Costs Scenario 1 (US$ ha^{-1})[b]	876	783	618	617	841	1034	573	658
Costs Scenario 2 (US$ ha^{-1})[c]	2654	2516	2430	2568	2615	2529	2154	2535
Yield Scen. 1 (Mg C ha^{-1} per million US$)[d]	5,838	7,048	4,269	5,631	4,935	6,234	5,752	5,615
Yield Scen. 2 (Mg C ha^{-1} per million US$)[d]	22,508	37,797	13,812	24,735	18,627	18,966	34,409	27,454

[a] Average disturbance level of fragments are scaled around zero and vary from high (negative values) to low (positive values).
[b] Costs for scenario 1 ('fragment restoration') vary around 750 US$ ha^{-1} according to the average disturbance level of biogeographical regions.
[c] Costs for scenario 2 ('landscape restoration') vary around 2500 US$ ha^{-1} according to the average forest cover of biogeographical regions.
[d] Yield is calculated by the amount of carbon increase in each scenario (Mg C ha^{-1}) divided by the total restoration costs (million US$).

Supplementary Table 7. Studies used to evaluate the relationship between above ground biomass and basal area in the Atlantic Forest.

For each study containing surveys with estimates of above ground biomass and basal area simultaneously, we present the Atlantic Forest formation studied, the number of surveys used in the analysis, their combined sampled area and dbh inclusion criteria.

Source	Forest formation	Number of surveys	Total effort (ha)	Dbh inclusion criteria (cm)
Ref. 53	Seasonal forests	78	28.7	≥10
Ref. 54	*Araucaria* forests	151	59.1	≥10
Ref. 55	Rainforests	201	72.1	≥10
Ref. 52	Seasonal and Rainforests	73	109.6	≥5

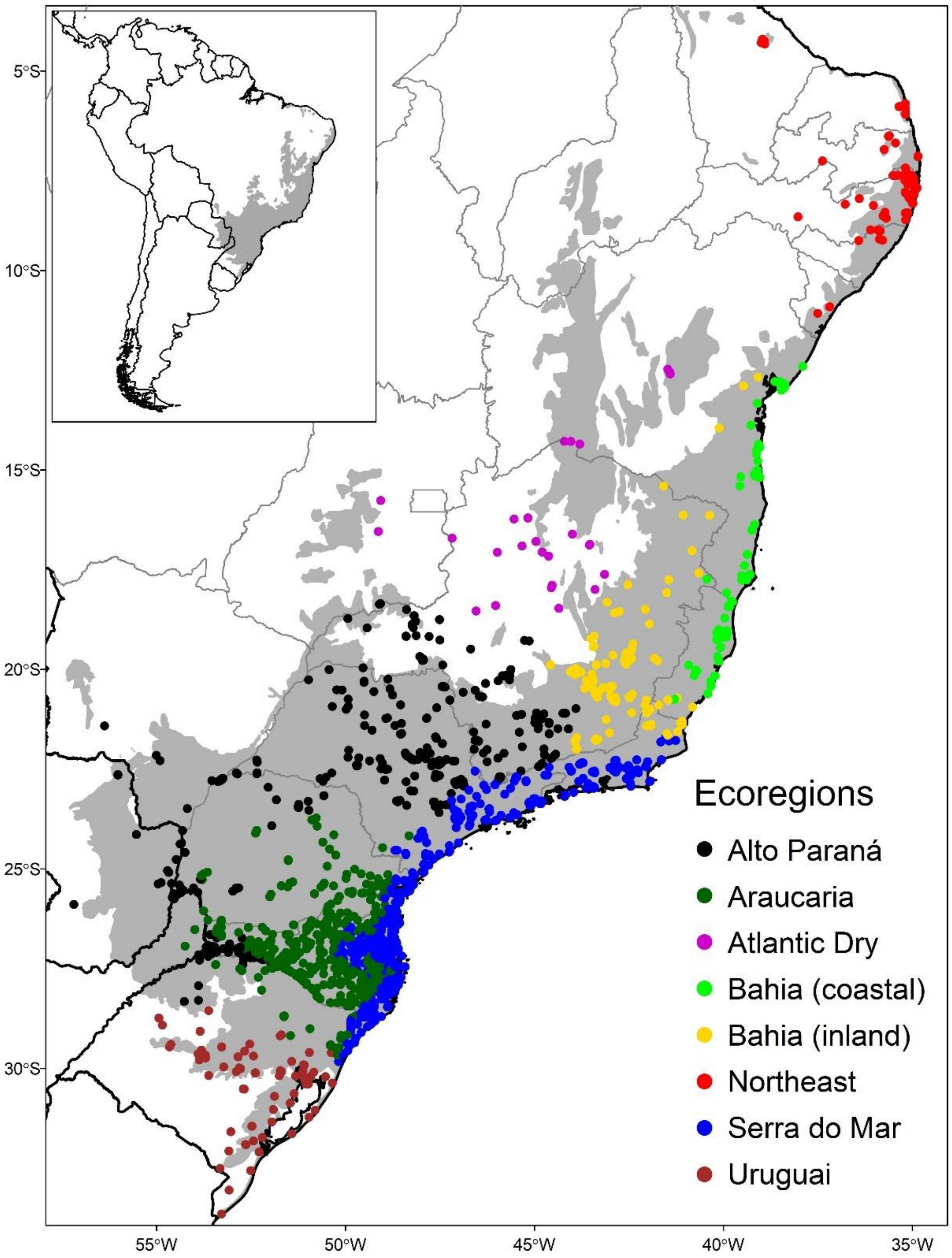

Supplementary Figure 1. The Atlantic Forest and the distribution of the tree community surveys used in this study.

The shaded grey corresponds to the Atlantic Forest limits and each circle represents one forest survey. Colours of circles correspond to the eight eco-regions used as a biogeographical control during data analyses. We considered surveys of forests classified by the original authors as Atlantic Forests, even if they were outside the Atlantic Forest limits.

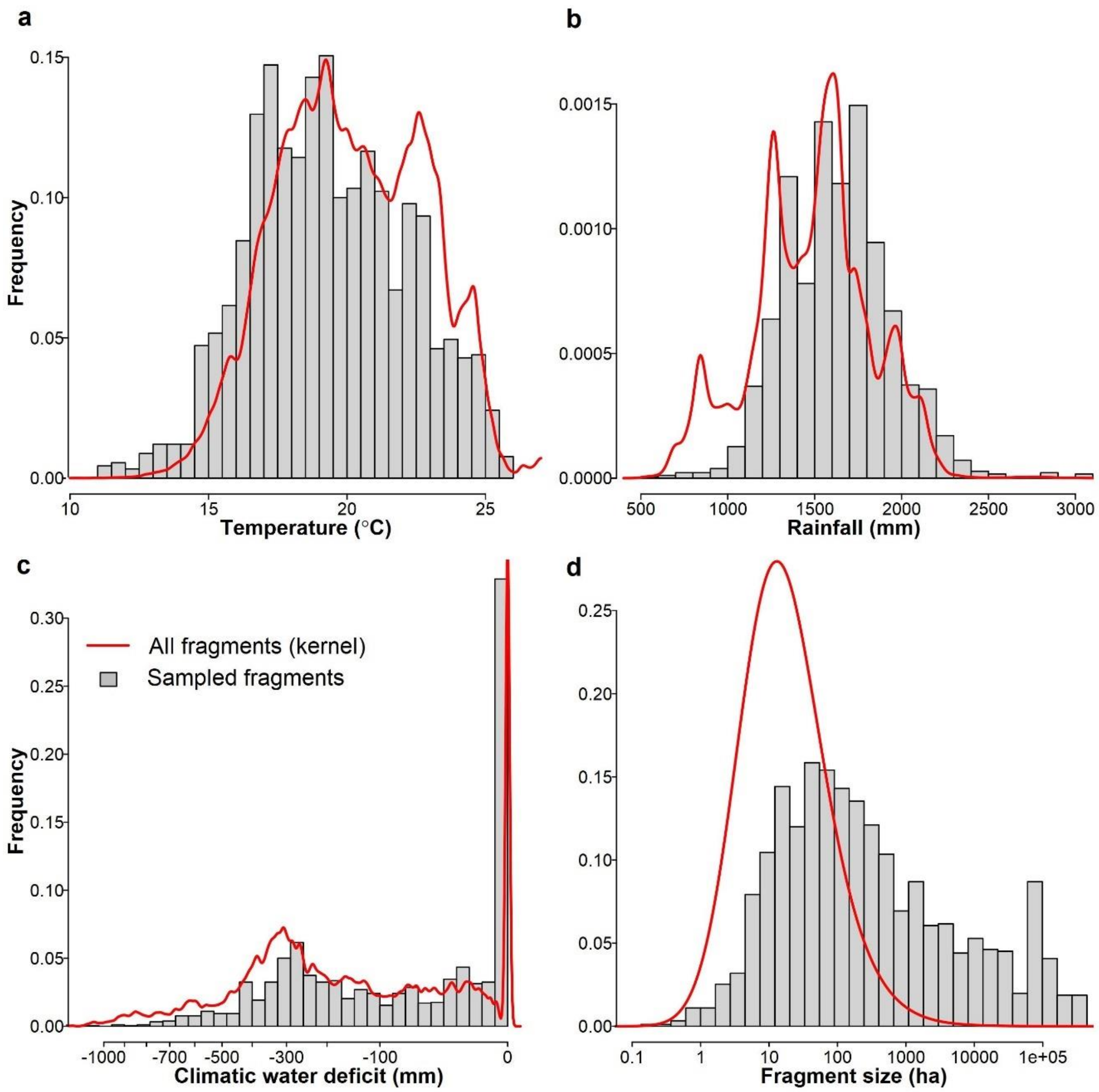

Supplementary Figure 2. Comparison of climate and fragment size between all fragments in the Atlantic Forest and the fragments included in our sample.

Panels represent the distribution of conditions in the surveys included in the analysis (grey bars; *n*=1,819 surveys) compared with forest fragments across the Atlantic Forest (red line; *n*=250,000) for (**a**) the distribution of mean annual air temperature; (**b**) mean annual rainfall; (**c**) climatic water deficit; and (**d**) forest fragment size for all Atlantic Forest fragments (red thick lines; *n*= ~250,000). Note that the *x*-axes for panels C and D are transformed.

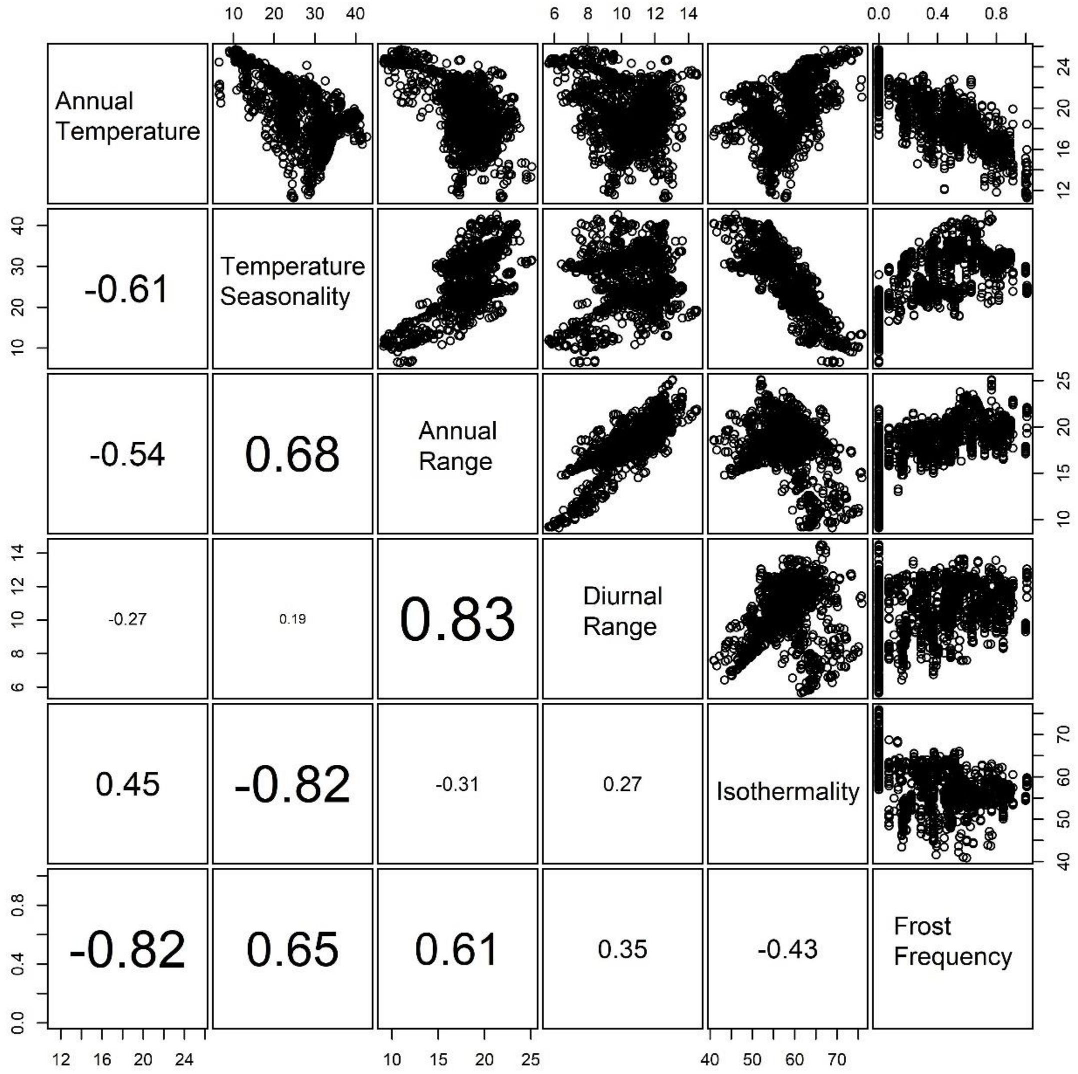

Supplementary Figure 3. Correlations between pairs of candidate explanatory variables related to air temperature and its seasonality.

The six temperature-related candidate explanatory variables (legends in the diagonal) that were considered during the construction of the linear mixed-effect models (see 'Methods' for definition and sources of each variable). Above the diagonal, the scatter plots between each pair of variables (each point represents a forest survey) are presented. The value of Pearson's correlation index for the corresponding pair of variables is given below the diagonal. The size of the font of the correlation index is proportional to the strength of the correlation.

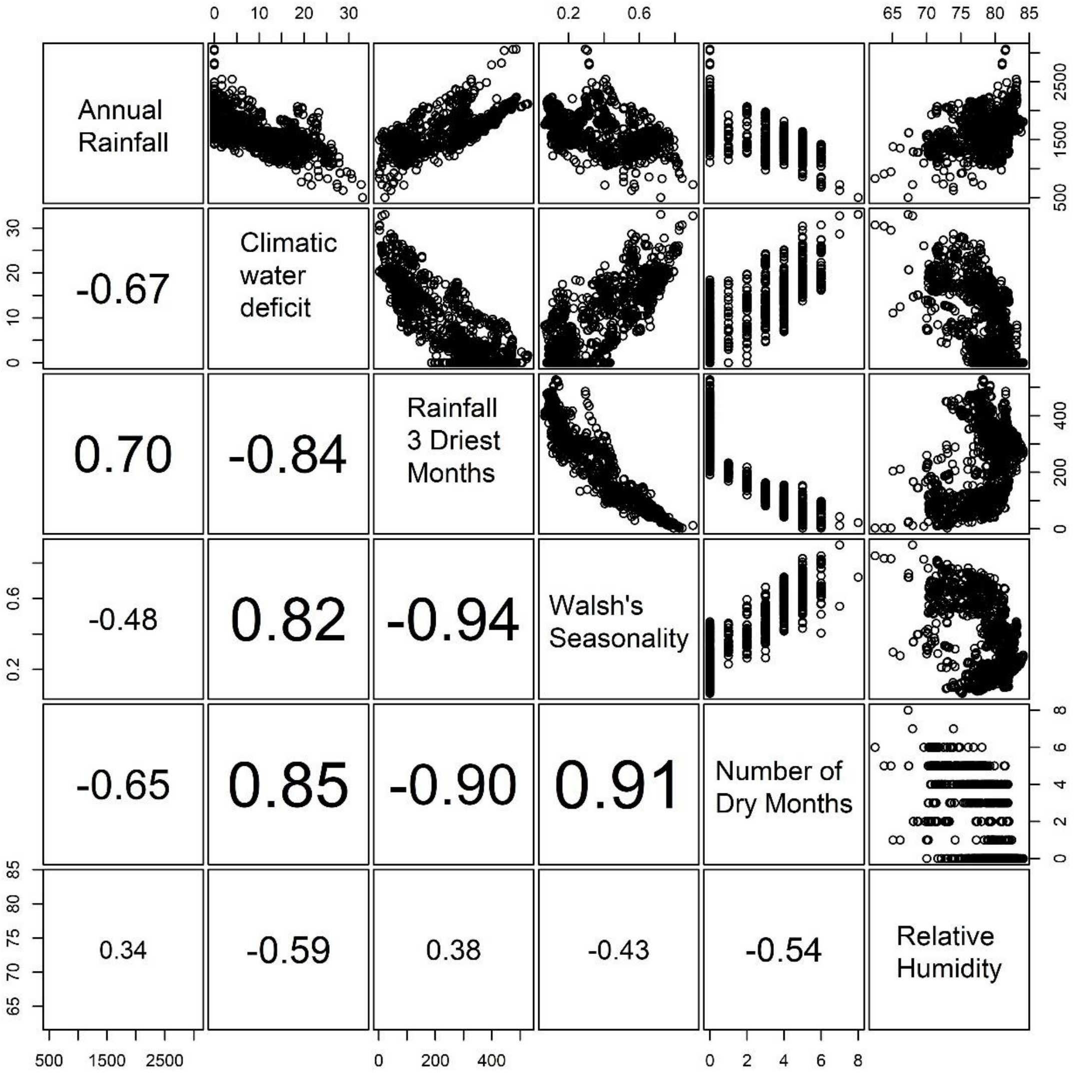

Supplementary Figure 4. Correlations between pairs of candidate explanatory variables related to rainfall and its variation in time.

The six rainfall-related candidate explanatory variables (legends in the diagonal) that were considered during the construction of the linear mixed-effect models (see 'Methods' for definition and sources of each variable). Above the diagonal, the scatter plots between each pair of variables (each point represents a forest survey) are presented. The value of Pearson's correlation index for the corresponding pair of variables is given below the diagonal. The size of the font of the correlation index is proportional to the strength of the correlation.

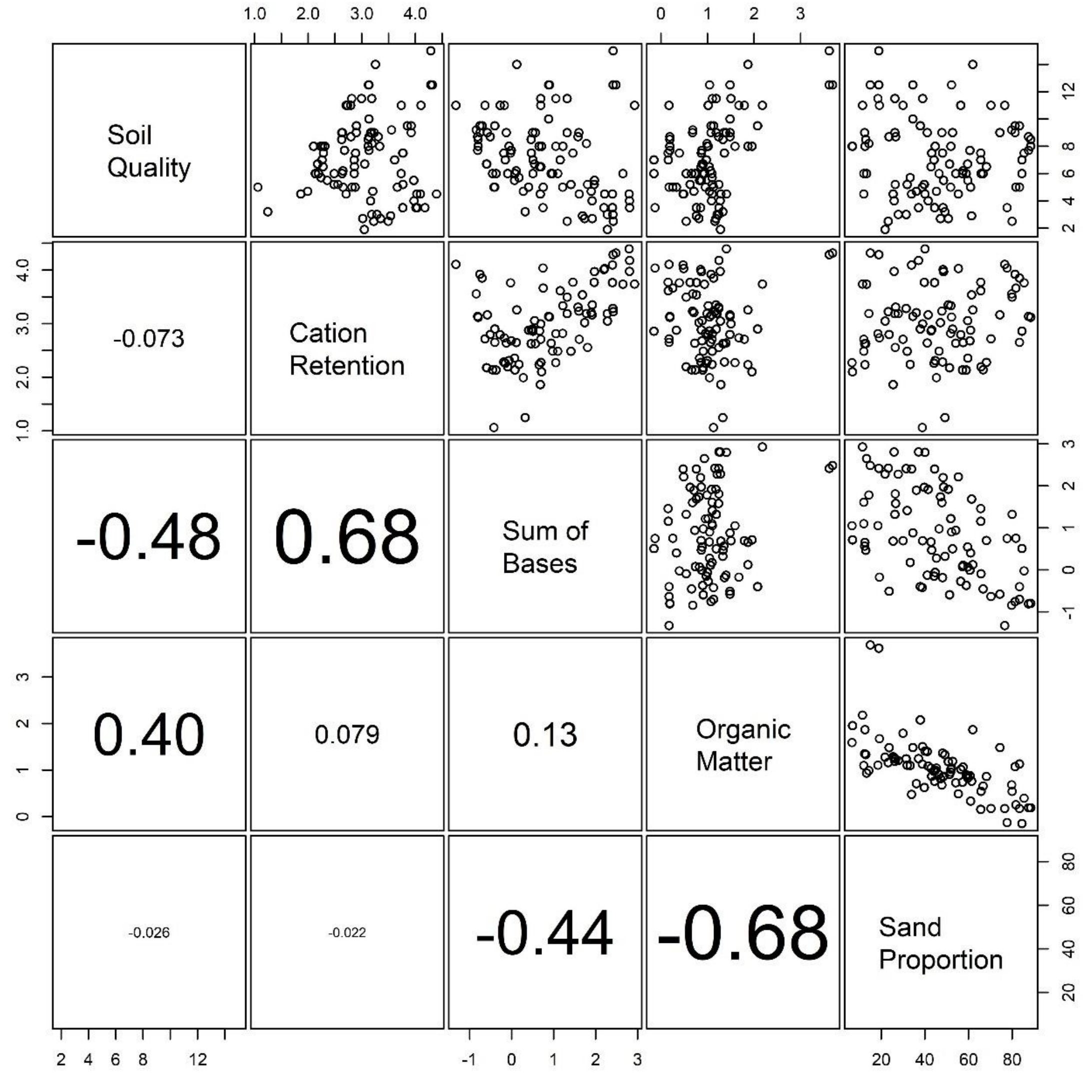

Supplementary Figure 5. Correlations between pairs of candidate explanatory variables related to soil properties.

The five soil-related candidate explanatory variables (legends in the diagonal) that were considered during the construction of the linear mixed-effect models (see ‘Methods’ for definition and sources of each variable). Above the diagonal, the scatter plots between each pair of variables (each point represents a forest survey) are presented. The value of Pearson's correlation index for the corresponding pair of variables is given below the diagonal. The size of the font of the correlation index is proportional to the strength of the correlation.

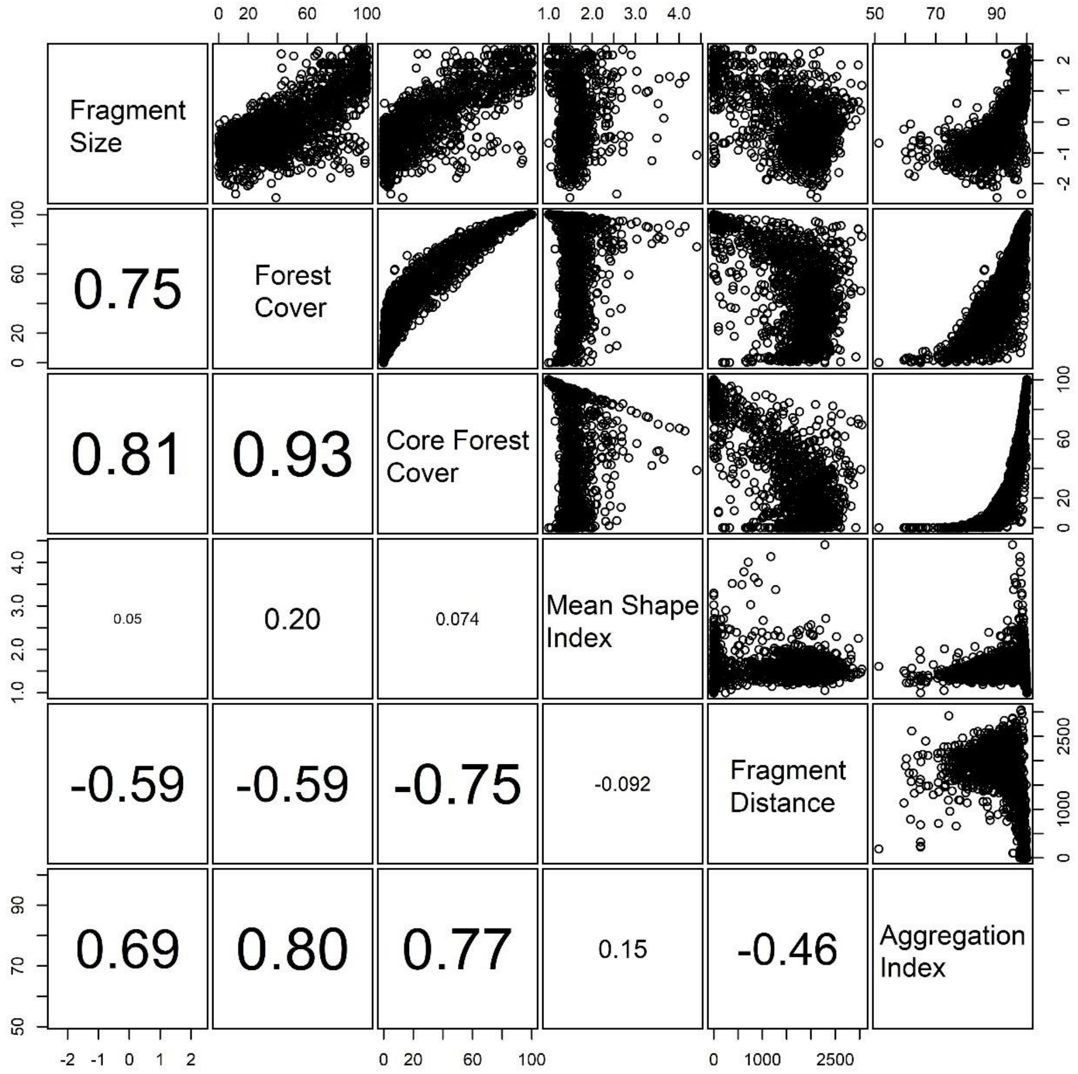

Supplementary Figure 6. Correlations between pairs of candidate explanatory variables related to fragment and landscape metrics.

The six patch and landscape metrics used as candidate explanatory variables (legends in the diagonal) during the construction of the linear mixed-effect models (see ‘Methods’ for definition and sources of each variable). Above the diagonal, the scatter plots between each pair of variables (each point represents a survey) are presented. The value of Pearson's correlation index for the corresponding pair of variables is given below the diagonal. The size of the font of the correlation index is proportional to the strength of the correlation.

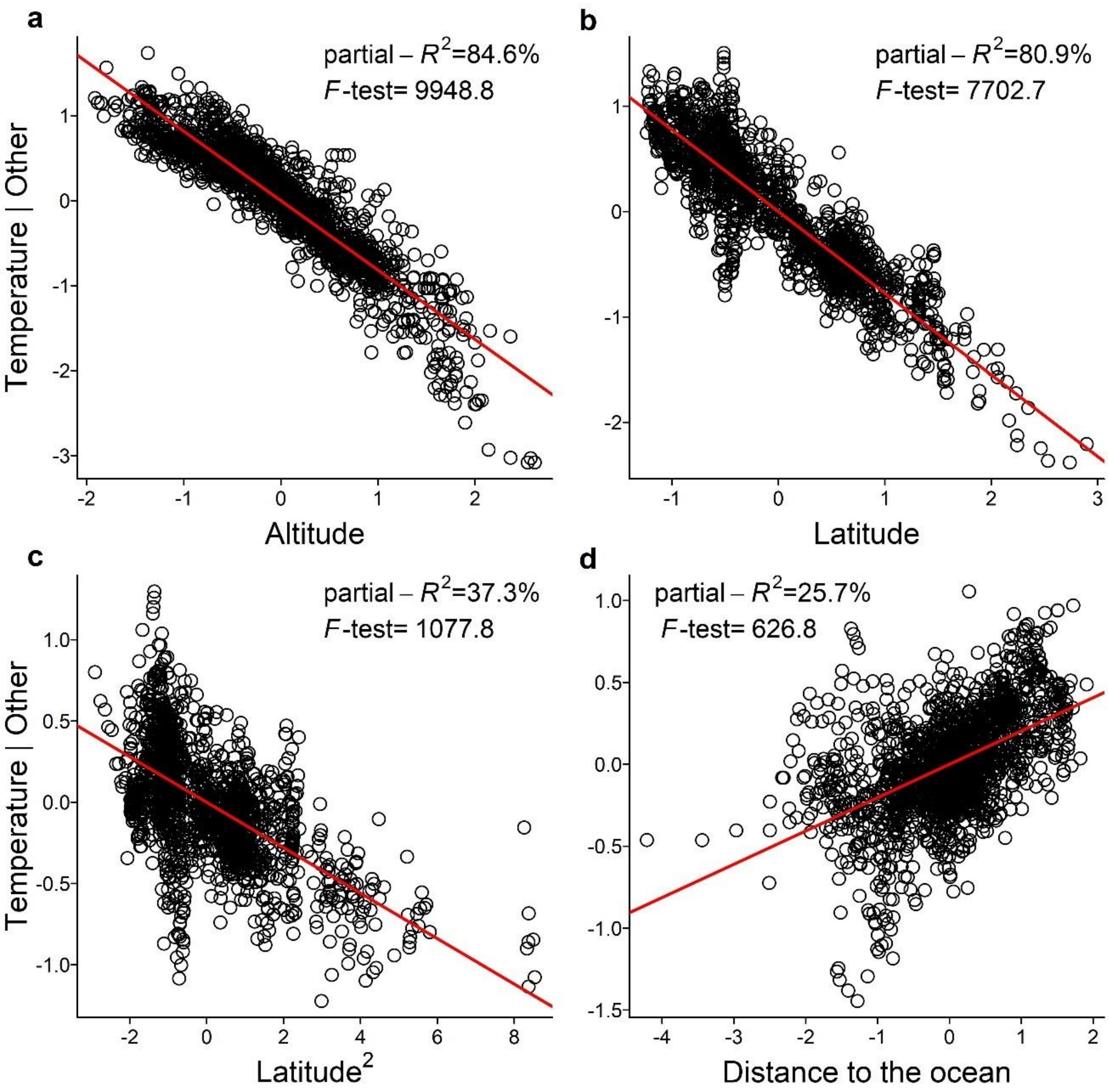

Supplementary Figure 7. Correlation between temperature and other temperature-related variables for the studied surveys.

Partial-regression plots presenting the results of the multiple linear regression model (*n*= 1820 surveys) relating mean air temperature to (**a**) the altitude above sea level; (**b**) latitude South; (**c**) the quadratic term for latitude; and (**d**) distance to the ocean. For each variable, the partial-R^2 and the F-test evaluating the effect of each variable is also given. For both axes, values are the residuals of each variable given the presence of all other variables in the model. The addition of the quadratic term for latitude significantly improved model fit with respect to the model containing only linear terms (ΔAIC= 855). The statistics for the full multiple regression model were: adjusted R^2= 92.3%; F-test: 5462; degrees of freedom: 1815; p-value: < 2.2e-16.

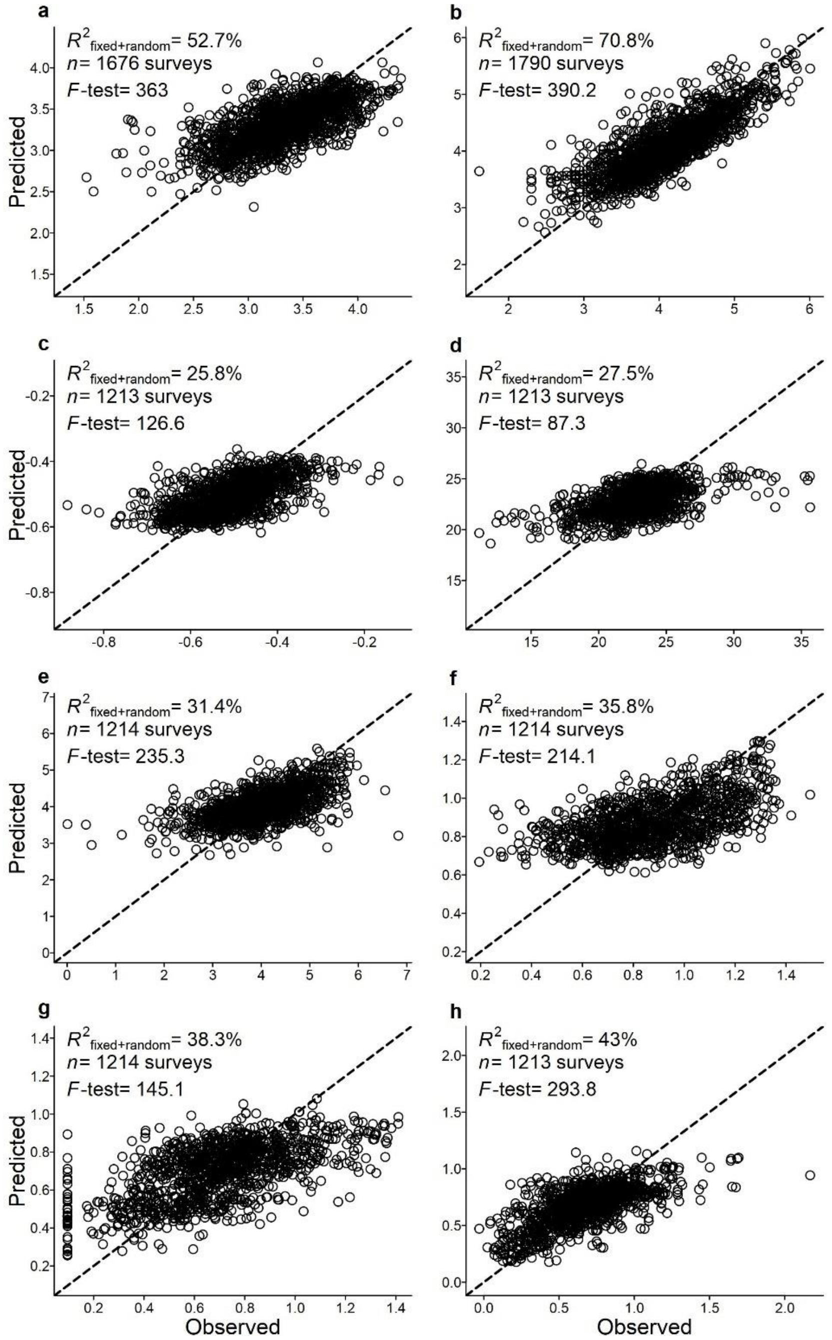

Supplementary Figure 8. Relationship between the observed and predicted values of the models fitted to forest biomass, species richness and species properties data. Each circle represents one survey regarding (**a**) forest biomass; (**b**) tree richness; (**c**) wood density; (**d**) maximum height; (**e**) seed mass; (**f**) ecological groups; (**g**) extinction threat; and (**h**) endemism level. The summary of the linear mixed-effects regression models is given in the top of each panel, where $R^2_{\text{fixed+random}}$ is the variation explained by the full model, *i.e*. the conditional R^2. Degrees of freedom: panel a= 1660, b= 1770, c= 1198, d–f= 1199, g= 1201, h= 1197. The dashed line represents the 1:1 ratio between observed and predicted values. Note that some of the axes were log- or power-transformed. All models have p-value< 2.2e-16, except Adult height (p-value= 2.5e-13).

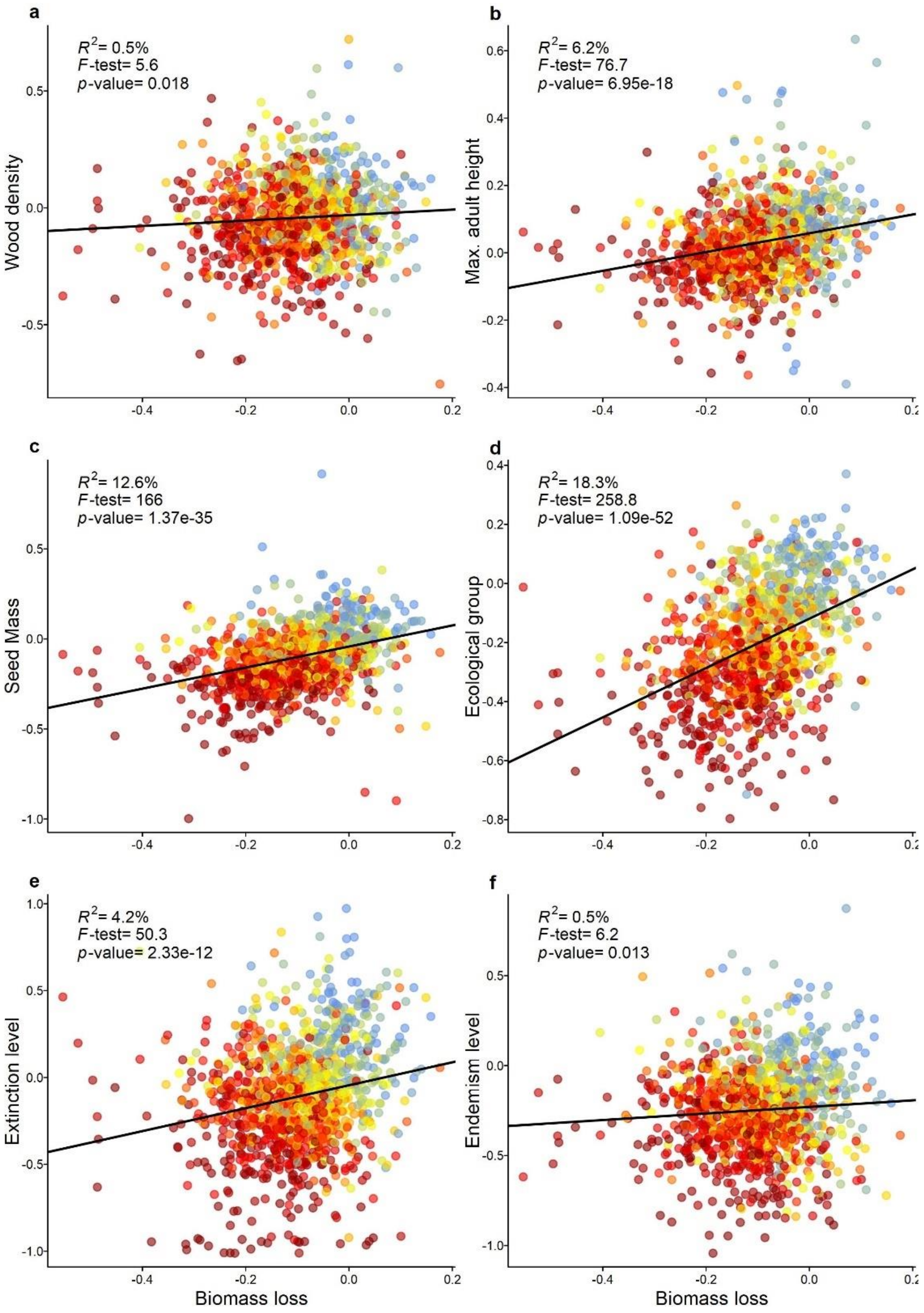

Supplementary Figure 9. The relationship between indices of loss of forest biomass and of the community-weighted mean of each species property.

Each point is a survey for which the loss of (**a**) wood density; (**b**) maximum height; (**c**) seed mass; (**d**) ecological groups; (**e**) threat of extinction; and (**f**) endemism level is available (n= 1153 surveys for all panels). The one-sided F-tests and the variation explained by the linear regression models (R^2) are given in the top of each panel (all models have 1152 degrees of freedom). The standardized index of loss is dimensionless and is highlighted by different colours ranging from dark red (high losses) to blue (gains).

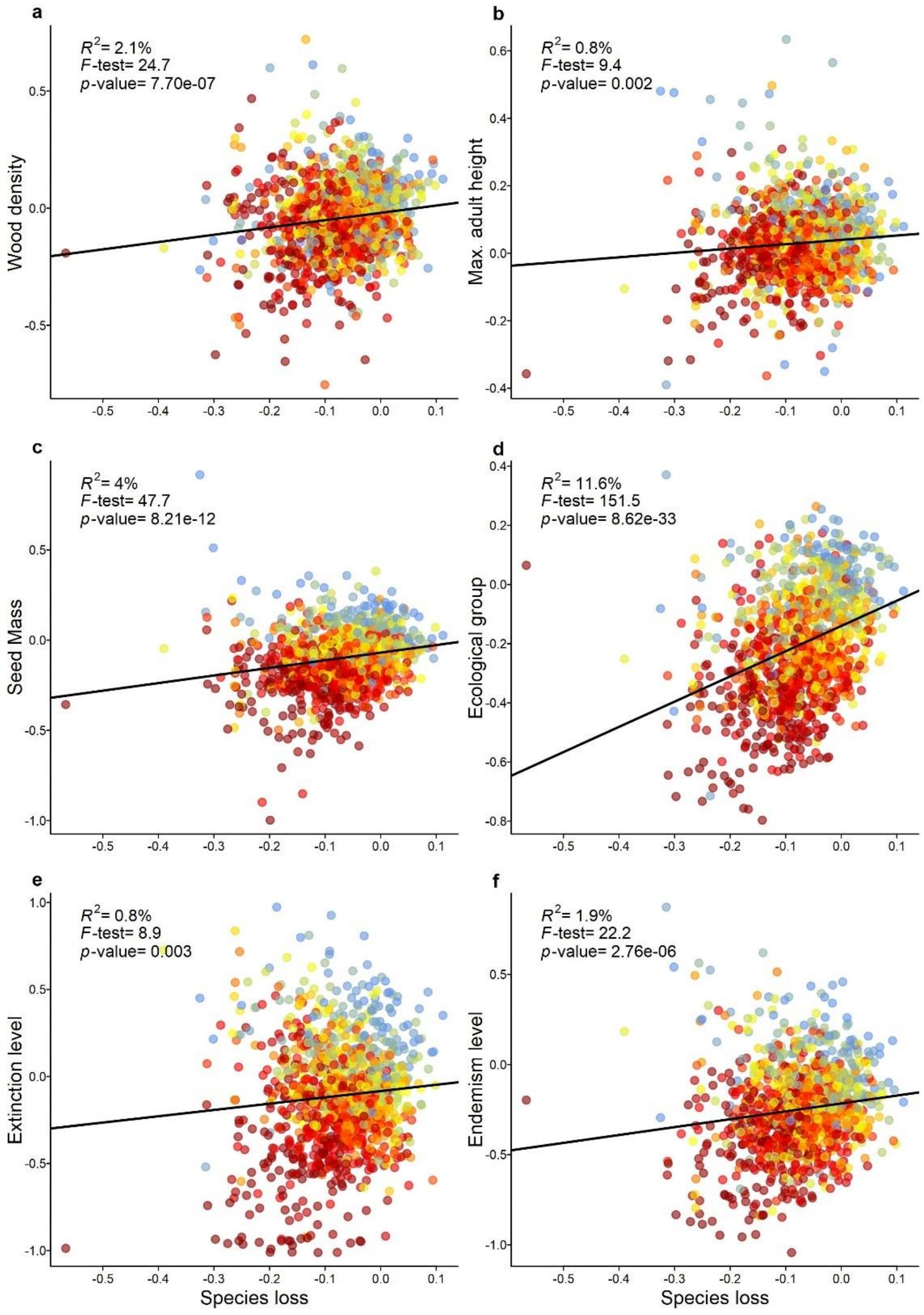

Supplementary Figure 10. The relationship between indices of loss of species richness and of the community-weighted mean of each species property. Each point is a survey for which the loss of (**a**) wood density; (**b**) maximum height; (**c**) seed mass; (**d**) ecological groups, (**e**) threat of extinction; and (**f**) endemism level is available (n= 1153 surveys for all panels). The one-sided F-tests and the variation explained by the linear regression models (R^2) are given in the top of each panel (all models have 1152 degrees of freedom). The standardized index of loss is dimensionless and is highlighted by different colours ranging from dark red (high losses) to blue (gains).

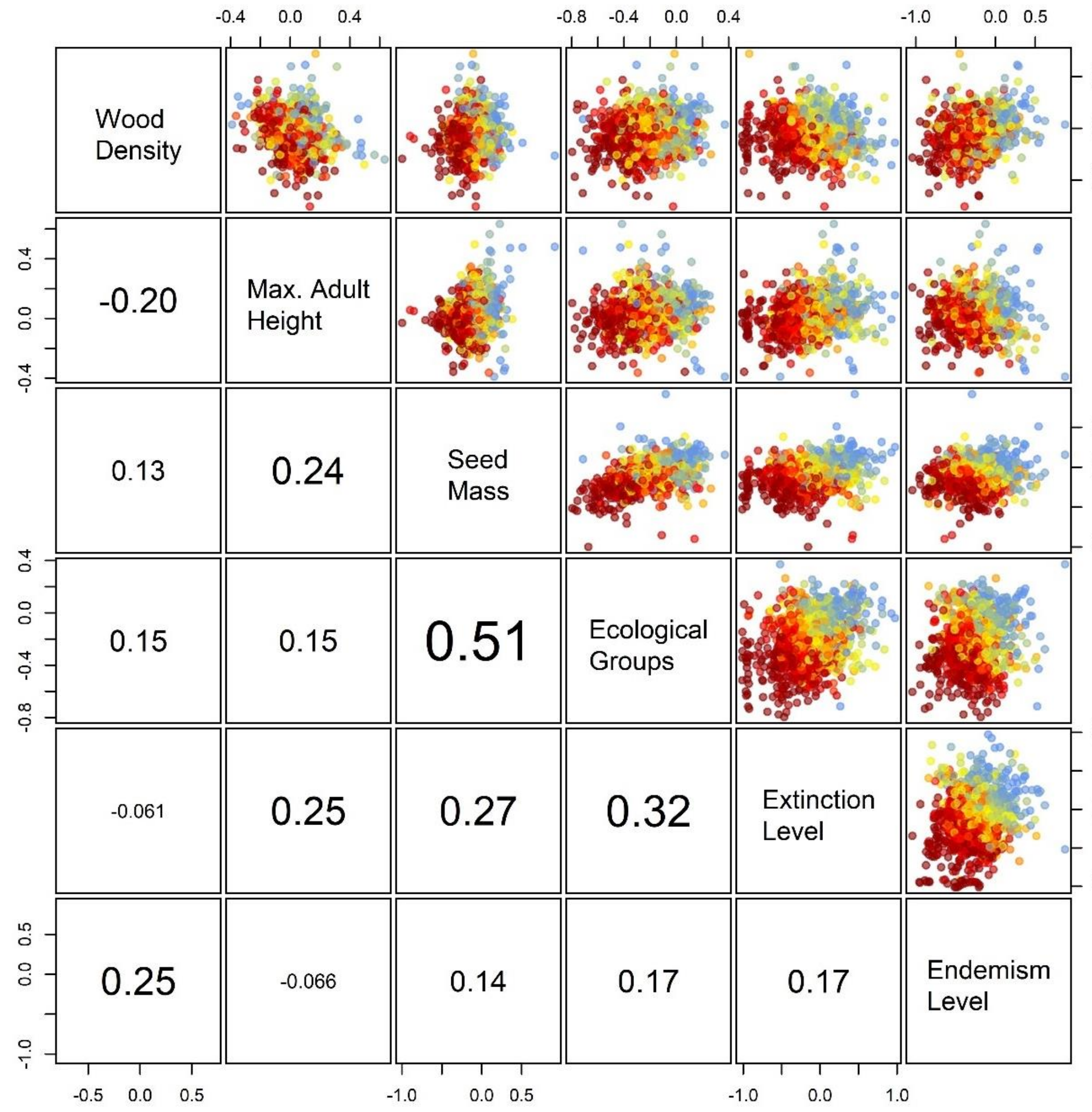

Supplementary Figure 11. The relationship among the standardized indices of loss for multiple species properties of the Atlantic Forest hotspot.

Above the diagonal, we present the scatterplots relating each pair of indices of loss for the six properties evaluated in this study (panels in the diagonal), in which every point represents a survey (*n*= 1213 surveys). Below the diagonal, we present the value of the Pearson's correlation index for the corresponding pair of properties. The size of the font of the correlation is proportional to the strength of the correlation between indices of loss. The standardized index of loss is dimensionless and is highlighted by different colours ranging from dark red (high losses) to blue (gains).

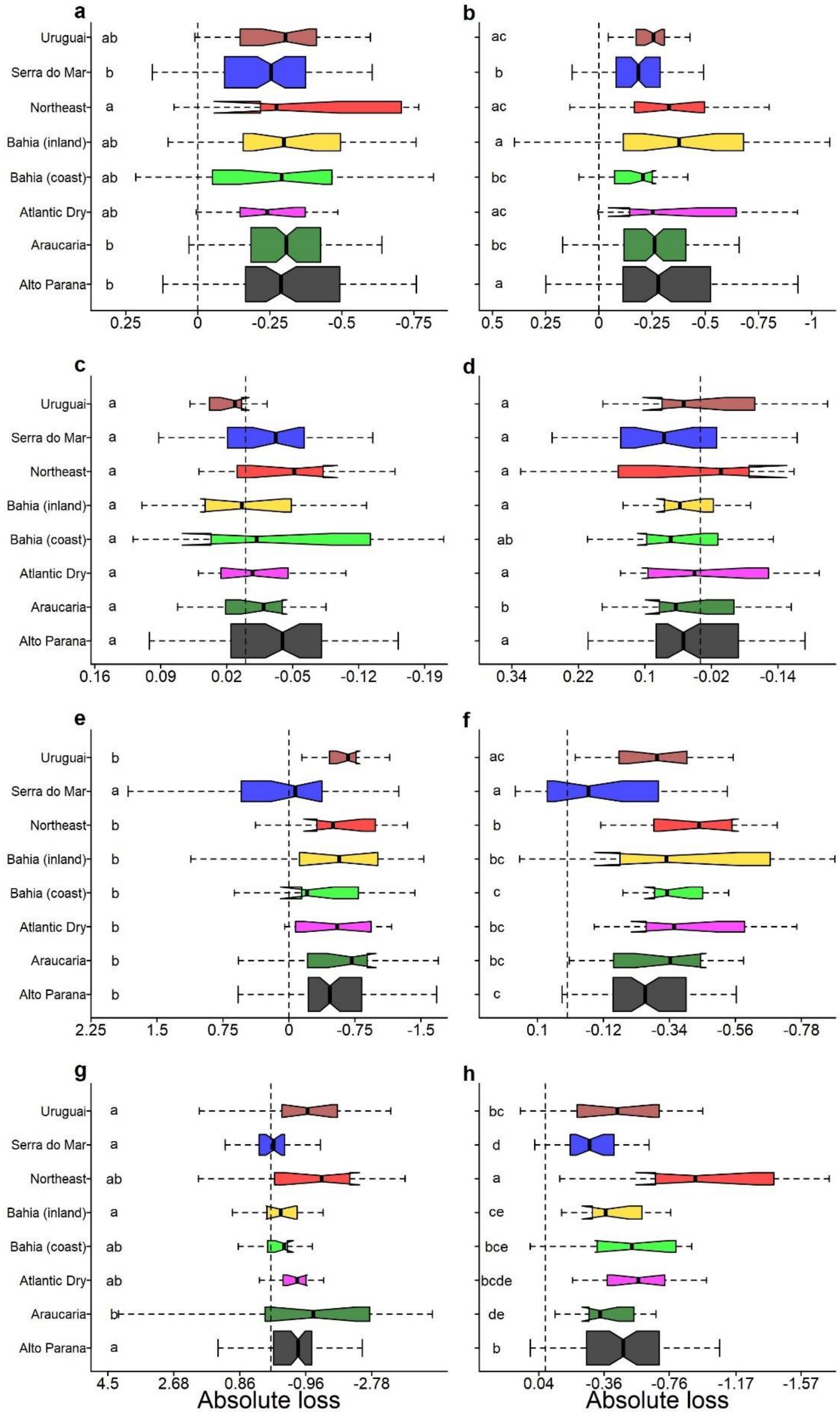

Supplementary Figure 12. The percentage of absolute loss of biomass, species richness and species properties by biogeographical region of the Atlantic Forest.

Weighted box-and-whisker plots of (**a**) forest biomass; (**b**) species richness; (**c**) wood density; (**d**) maximum height; (**e**) seed mass; (**f**) ecological groups; (**g**) threat of extinction; and (**h**) endemism level. These plots summarize the distribution of losses for each region (*i.e*. vertical bold line, median; box limits, upper and lower quartiles; whiskers, 5 and 95% quantiles). Outliers of the distributions are not presented for clarity. Dashed lines separate gains from losses due to human-related impacts. The result of the Tukey's honest significance test (one-sided, with an adjustment for group sample sizes) for difference among group means (lowercase letters) is also presented. Colours represent the different Atlantic Forest regions as in Fig. S1. The number of surveys dbh$\geq$4.8-5.0, the one-sided F-test statistics and the p-value of the panels are: panel a: n= 962, F= 3.9, p= 0.0003; b: n= 1039, F= 15.3, p< 2.2e-16; c: n= 671, F= 0.69, p= 0.678; d: n= 671, F= 3.2, p= 0.0022; e: n= 671, F=9.5, p= 2.52e-11; f: n= 671, F= 7.7, p= 5.9e-09; g: n= 671, F= 3.2, p= 0.0028; h: n= 671, F=12.1, p= 1.31e-14.

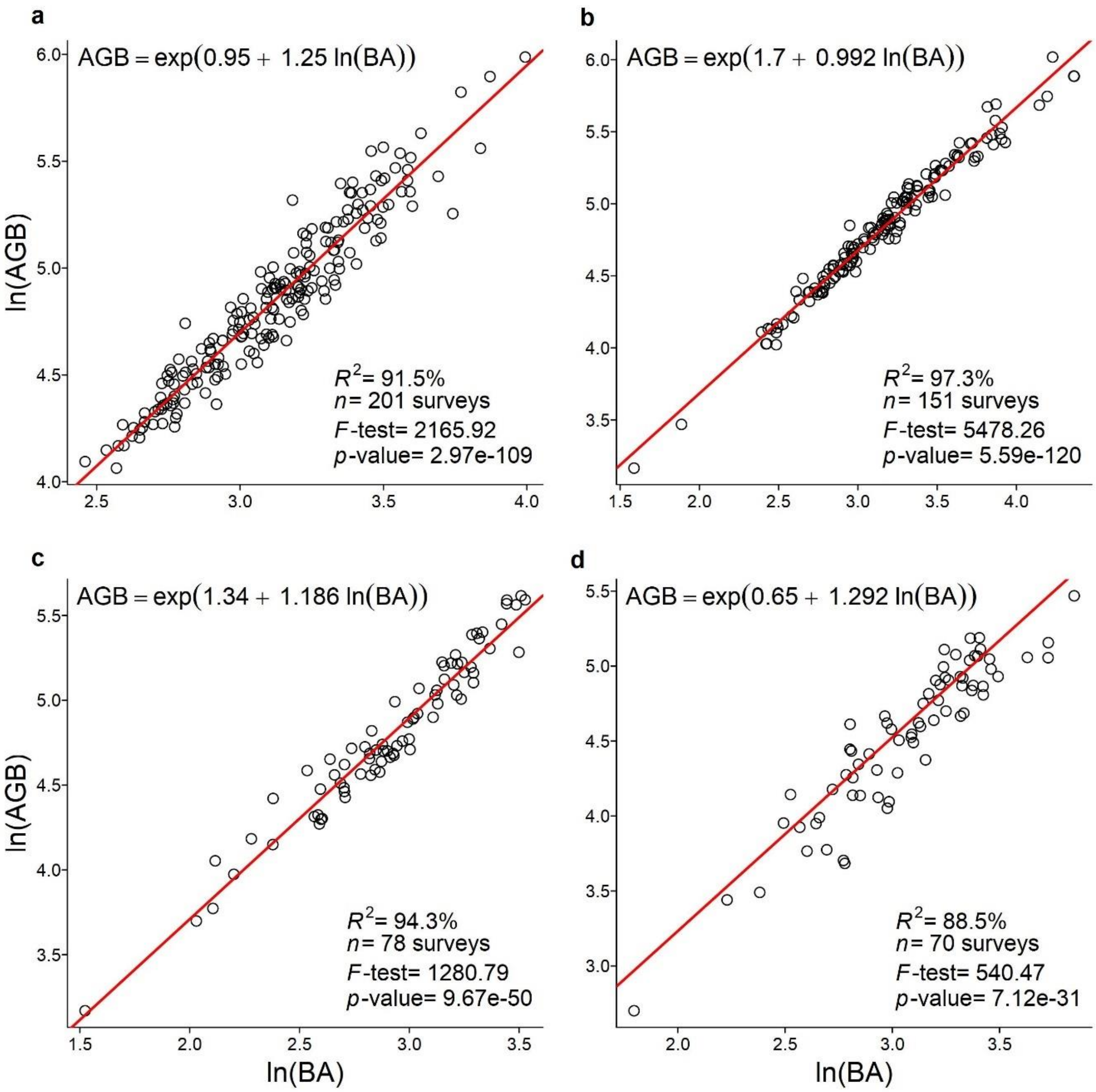

Supplementary Figure 13. Correlation between tree basal area and above ground biomass in different Atlantic Forest formations.

Mean prediction of the linear regression model relating basal area (in m^2 ha^{-1}) to above-ground biomass (in Mg ha^{-1}) for (**a**) seasonal forests; (**b**) *Araucaria* forests; (**c**) rain forests of Santa Catarina state; and (**d**) for seasonal plus rain forests of Minas Gerais state. The statistical model is given in the top of each panel and the model statistics are provided below each panel. For panel d, the legend corresponds to the result of the weighted regression model, which contained the total sampling effort (in ha) of the forest surveys as weights.
