# Supplementary material for: The erosion of biodiversity and biomass in the Atlantic Forest biodiversity hotspot
Source: Nat Commun. 2020 Dec 11;11:6347. doi: 10.1038/s41467-020-20217-w (PMC7733445; doi:10.1038/s41467-020-20217-w)
Supplement: Supplementary file 2 — Description of Additional Supplementary Files [file 41467_2020_20217_MOESM2_ESM.pdf]

Description of Additional Supplementary Files

Supplementary Data 1. The list of surveys extracted from the TreeCo database used in the analysis, together with their corresponding metadata.

Supplementary Data 2. The list of species to support the Atlantic Forest restoration and their average ranks of carbon storage potential, ecological interactions, and conservation status, as well as their frequency in each biogeographical region of the Atlantic Forest.

Supplementary Data 3. The full list of sources used for the compilation of species trait information and their corresponding TreeCo reference ID.
