# Supplementary material for: The erosion of biodiversity and biomass in the Atlantic Forest biodiversity hotspot
Source: Nat Commun. 2020 Dec 11;11:6347. doi: 10.1038/s41467-020-20217-w (PMC7733445; doi:10.1038/s41467-020-20217-w)
Supplement: Supplementary file 5 — Supplementary Data 3 [file 41467_2020_20217_MOESM5_ESM.pdf]

The erosion of biodiversity and biomass in the Atlantic Forest biodiversity hotspot

Renato A. F. de Lima[1,2]*, Alexandre A. Oliveira[1], Gregory R. Pitta[1], André L. de Gasper[3], Alexander C. Vibrans[4], Jérôme Chave[5], Hans ter Steege[2,6] & Paulo I. Prado[1]

[1] Departamento de Ecologia, Instituto de Biociências, Universidade de São Paulo. Rua do Matão, trav. 14, 321, 05508-090, São Paulo, Brazil.

[2] Naturalis Biodiversity Center, Darwinweg 2, 2333 CR Leiden, The Netherlands.

[3] Departamento de Ciências Naturais, Universidade Regional de Blumenau. Rua Antônio da Veiga, 140, 89030-903, Blumenau, Brazil.

[4] Departamento de Engenharia Florestal, Universidade Regional de Blumenau. Rua São Paulo, 3250, 89030-000, Blumenau, Brazil.

[5] Laboratoire Evolution et Diversité Biologique, UMR 5174 CNRS, Université Paul Sabatier, IRD. 118, route de Narbonne, 31062, Toulouse, France.

[6] Systems Ecology, Vrije Universiteit, De Boelelaan 1087, Amsterdam, 1081 HV, Netherlands

*e-mail: raflima@usp.br

This PDF file includes:

Supplementary Data 3 2

Supplementary Data 3. The full list of sources used for the compilation of species trait information and their corresponding TreeCo reference ID.

ABREU, K.M.P.; SILVA, G.F. & SILVA, A.G. 2013. Análise fitossociológica da Floresta Nacional de Pacotuba, Cachoeiro de Itapemirim, ES – Brasil. Cerne 19(1): 157-168. [TreeCo refID: 7]

ALMEIDA, S.D.; PROENÇA, C.E.; SANO, S.M. & RIBEIRO, J.F. 1998. Cerrado: espécies vegetais úteis. Embrapa-CPAC, Planaltina. 464p. [TreeCo refID: 10121]

ALMEIDA, V.C. 1996. Composição florística e estrutura do estrato arbóreo de uma floresta situada na Zona da Mata Mineira, município de Lima Duarte, MG. Dissertação (Mestrado). Universidade Federal do Rio de Janeiro, Rio de Janeiro. 89p. [TreeCo refID: 136]

AMARO, M.A. 2010. Quantificação do estoque de volume, biomassa e carbono em uma Floresta Estacional Semidecidual Montana em Viçosa, MG. Tese (doutorado). Universidade Federal de Viçosa, Viçosa. 168p. [TreeCo refID: 10003]

AMORIM, A.M.; CARVALHO, G.M. & MATOS, F. 2009. Relatório Técnico dos Diagnósticos Temáticos do Grupo FLORA - PARNA Descobrimento, Bahia, Brasil. Fundação Biodiversitas/SAVE Brasil/IESB/CEPLAC/UFMG. 81 p. [TreeCo refID: 2747]

AMORIM, A.M.; JARDIM, J.G. & FIASCHI, P. 2007. Relatório Técnico dos Diagnósticos Temáticos do Grupo FLORA - Ampliação e Estabelecimentos de novas RPPNs no sul da Bahia. Instituto de Estudos Sócio Ambientais do Sul da Bahia (IESB). [TreeCo refID: 2750]

AMORIM, I.L.; SAMPAIO, E.V.S.B. & ARAÚJO, E.L. 2005. Flora e estrutura da vegetação arbustivo-arbórea de uma área de caatinga do Seridó, RN, Brasil. Acta Botanica Brasilica 19(3): 615-623. [TreeCo refID: 2176]

ANTONIO, F. & GIULIETTI, A. 2014. A tribo Pisonieae Meisner (Nyctaginaceae) no Brasil. Boletim De Botânica 32(2): 145-268. [TreeCo refID: 10189]

ARCHANJO, K.M.P.A.; SILVA, G.F.; CHICHORRO, J.F. & SOARES, C.P.B. 2012. Estrutura do componente arbóreo da Reserva Particular do Patrimônio Natural Cafundó, Cachoeiro de Itapemirim, Espírito Santo, Brasil. Floresta 42 (1): 145-160. [TreeCo refID: 6]

ARRUDA, D.M.; BRANDÃO, D.O.; COSTA, F.V.; TOLENTINO, G.S.; BRASIL, R.D.; D´ÂNGELO NETO, S. & NUNES, Y.R.F. 2011. Structural aspects and floristic similarity among tropical dry forest fragments with different management histories in northern Minas Gerais, Brazil. Revista Árvore 35(1): 131-142. [TreeCo refID: 62]

ASSIS, L.C.S. 2009. Sistematica e Filosofia: filogenia do complexo Ocotea e revisao do grupo Ocotea indecora (Lauraceae). Tese (doutorado). Universidade de São Paulo, São Paulo. 238 p. [TreeCo refID: 10004]

BARBÉRIO, M. 2013. Maturação de sementes de Andira fraxinifolia Benth. (Fabaceae) em uma área de restinga. Dissertação (Mestrado). Instituto de Botânica, São Paulo. 53p. [TreeCo refID: 10409]

BELLO, C.; GALETTI, M.; MONTAN, D.; PIZO, M.A.; MARIGUELA, T.C.; CULOT, L.; BUFALO F.S.; LABECCA, F.M.; PEDROSA, F.R.V.; CONSTANTINI, R.; EMER, C.; SILVA, W.R.; SILVA, F.R.; OVASKAINEN, O. & JORDANO, P. 2017. Atlantic frugivory: a plant–frugivore interaction data set for the Atlantic Forest. Ecology 98(6): 1729. [TreeCo refID: 10418]

BENVENUTI-FERREIRA, G.; COELHO, G.C. 2009. Floristics and structure of the tree component in a Seasonal Forest remnant, Chiapetta, Rio Grande do Sul State, Brazil. Revista Brasileira de Biociências 7(4): 344-353. [TreeCo refID: 754]

BERG, C. 2001. Moreae, Artocarpeae, and Dorstenia (Moraceae), with Introductions to the Family and Ficus and with Additions and Corrections to Flora Neotropica Monograph 7. Flora Neotropica 83: iii-346. [TreeCo refID: 10138]

BERG, C.; ROSSELLI, P.F. & DAVIDSON, D. 2005. Cecropia. Flora Neotropica 94: 1-230. [TreeCo refID: 10139]

BERNACCI, L.C.; FRANCO, G.A.D.C. & ÁRBOCZ, G.F. 2006. O efeito da fragmentação florestal na composição e riqueza de árvores na região da Reserva Morro Grande (Planalto de Ibiúna, SP). Revista do Instituto Florestal 18: 121-166. [TreeCo refID: 2690]

BIANCHINI, E.B.; POPOLO, R.S.; DIAS, M.C. & PIMENTA, J.A. 2003. Diversidade e estrutura de espécies arbóreas em uma área alagável do município de Londrina, sul do Brasil. Acta Botanica Brasilica 17(3): 405-419. [TreeCo refID: 449]

BIGIO, N.C. & SECCO, R.S. 2012. As espécies de Pera (Euphorbiaceae s.s) na Amazônia brasileira. Rodriguésia 63(1): 163-207. [TreeCo refID: 10157]

BORGHI, W.A.; MARTINS, S.S.; DEL QUIQUI, E.M. & NANNI, M.R. 2004. Caracterização e avaliação da mata ciliar à montante da Hidrelétrica de Rosana, na Estação Ecológica do Caiuá, Diamante do Norte, PR. Cadernos de Biodiversidade 4(2): 9-18. [TreeCo refID: 454]

BOSA, D.M. 2011. Composição florística e estrutural de comunidade arbórea de floresta ombrófila densa montana no município de Morro Grande, Santa Catarina. Dissertação (Mestrado). Universidade do Extremo Sul Catarinense, Criciúma. 89p. [TreeCo refID: 833]

BOTREL, R.T.; OLIVEIRA-FILHO, A.T.; RODRIGUES, L.A. & CURI, N. 2002. Composição florística e estrutura da comunidade arbórea de um fragmento de floresta estacional semidecidual em Ingaí, MG, e a influência de variáveis ambientais na distribuição das espécies. Revista Brasileira de Botânica 25(2): 195-213. [TreeCo refID: 66]

BRANDÃO, C.F.L.S.; MARANGON, L.C.; FERREIRA, R.L.C. & LINS-E-SILVA, A.C.B. 2009. Phytosociological structure and successional classification of arboreus component in a fragment of a dense ombrophylous forest, Igarassu – Pernambuco. Revista Brasileira de Ciências Agrárias 4(1): 55-61. [TreeCo refID: 1515]

BROTTO, M.L.; CERVI, A.C. & SANTOS, E.P. 2013. O gênero Ocotea (Lauraceae) no estado do Paraná, Brasil. Rodriguésia 64(3): 495-525. [TreeCo refID: 10008]

BROWN, S. 1997. Appendix 1 - List of wood densities for tree species from tropical America, Africa, and Asia. In: Estimating Biomass and Biomass Change of Tropical Forests: a Primer.

UN FAO Forestry Paper, 134. <http://www.fao.org/docrep/w4095e/w4095e00.htm#Contents > Acessado em: 23 fev 2016. [TreeCo refID: 10009]

BUDKE, J.C. 2007. Pulsos de inundação, padrões de diversidade e distribuição de espécies arbóreas em uma floresta ribeirinha no sul do Brasil, Tese (Doutorado). Universidade Federal do Rio Grande do Sul, Porto Alegre, RS. 195p. [TreeCo refID: 663]

BUDKE, J.C.; JARENKOW, J. A. & OLIVEIRA-FILHO, A.T. 2008. Tree community features of two stands of riverine under different flooding regimes in Southern Brazil. Flora 203(2): 162-174. [TreeCo refID: 664]

CADDAH, M.K. 2013. Estudos taxônomicos e filogenéticos em Miconia sect. Discolor (Melastomataceae, Miconieae). Tese (Doutorado). Universidade Estadual de Campinas, Campinas. 261 p. [TreeCo refID: 10186]

CALIARI, C.P. 2013. Estudos em Myrtaceae do Estado de São Paulo: Myrcia seção Gomidesia. Dissertação (Mestrado). Escola Superior de Agricultura "Luiz de Queiroz", Piracicaba. 129p. [TreeCo refID: 10010]

CAMPOE, O.C. 2008. Efeito de práticas silviculturais sobre a produtividade líquida de madeira, o índice de área foliar e a eficiência do uso da luz em de restauração da Mata Atlântica. Dissertação (Mestrado). Escola Superior de Agricultura "Luiz de Queiroz", Piracicaba. 120p. [TreeCo refID: 10011]

CAMPOS, J.B. & SOUZA, M.C. 2003. Potential for natural Forest regeneration from seed bank in an Upper Paraná River Floodplain, Brazil. Brazilian Archives of Biology and Technology 46(4): 625-639. [TreeCo refID: 2686]

CAMPOS, J.B.; ROMAGNOLO, M.B. & SOUZA, M.C. 2000. Structure, composition and spatial distribution of tree species in a remnant of the semideciduous seasonal alluvial forest of the upper Paraná River floodplain. Brazilian Archives of Biology and Technology 43(2): 185-194. [TreeCo refID: 509]

CANALE, G.R.; SUSCKE, P.; ROCHA-SANTOS, L.; SÃO BERNARDO, C.S.; KIERULFF, M.C.M. & CHIVERS, D.J. 2016. Seed Dispersal of Threatened Tree Species by a Critically Endangered Primate in a Brazilian Hotspot. Folia Primatologica 87(3): 123-140. [TreeCo refID: 10012]

CAPRETZ, R.L.; BRITEZ, R.M.; BEBBER, D.P.; REGINATO, M. & ZWIENER, V.P. (Unpublished data). Floristic and structural patterns along a successional chronosequence in the Atlantic Forest of Southern Brazil. UFPR. Curitiba. [TreeCo refID: 2714]

CARAUTA, J.P.P. & DIAZ, B.E. 2002. Figueiras no Brasil. Editora UFRJ, Rio de Janeiro. 212p. [TreeCo refID: 10013]

CARMELLO-GUERREIRO, S.M. & PAOLI, A.A.S. 2005. Anatomy of the pericarp and seed-coat of Lithraea molleoides (Vell.) Engl. (Anacardiaceae) with taxonomic notes. Brazilian Archives of Biology and Technology, 48(4): 599-610. [TreeCo refID: 10014]

CARUZO, M.B.R. 2010. Sistematica de Croton sect. Cleodora (Euphorbiaceae s.s.). Tese (Doutorado). Universidade de Sao Paulo, São Paulo. 273p. [TreeCo refID: 10015]

CARVALHO-OKANO, R.M. 1992. Estudos taxonômicos do gênero Maytenus Mol. emend. Mol. (Celastraceae) do Brasil extra-amazônico. Tese (Doutorado). Universidade Estadual de Campinas, Campinas. 261p. [TreeCo refID: 10184]

CARVALHO, A.F. 2013. Caracterização da madeira e do carvão vegetal produzido a partir de cinco espécies florestais utilizadas na região de Biguaçu, SC. Dissertação (Mestrado). Universidade do Estado de Santa Catarina, Lages. 142p. [TreeCo refID: 10016]

CARVALHO, A.M. & AMORIM, A.M. 1996. Composição Florística e Estrutura da Mata da Esperança no município de Ilhéus, BA. In: XLVII Congresso Nacional de Botânica. [TreeCo refID: 2749]

CARVALHO, D.A.; OLIVEIRA-FILHO, A.T. & VILELA, E.A. 1999. Floristics and phytosociology of the arboreal-shrubby vegetattion of a deciduous riparian forest of the low Paranaiba (Santa Vitoria, Minas Gerais). Revista Arvore 23(3): 311-320. [TreeCo refID: 151]

CARVALHO, D.A.; OLIVEIRA-FILHO, A.T.; VAN DEN BERG, E.; FONTES, M.A.L.; VILELA, E.A.; MARQUES, J.J.S.G.S. M. & CARVALHO, W.A.C. 2005. Variações florísticas e estruturais do componente arbóreo de uma floresta ombrófila alto-montana às margens do Rio Grande, Bocaina de Minas, MG, Brasil. Acta Botanica Brasilica 19 (1): 91-109. [TreeCo refID: 69]

CARVALHO, D.A.; OLIVEIRA-FILHO, A.T.; VILELA, E.A. & CURI, N. 2000. Florística e estrutura da vegetação arbórea de um fragmento de floresta semidecidual às margens do reservatório da Usina Hidrelétrica Dona Rita (Itambé do Mato Dentro, MG). Acta Botanica Brasilica 14(1): 37-55. [TreeCo refID: 68]

CARVALHO, D.A.; OLIVEIRA-FILHO, A.T.; VILELA, E.A.; CURI, N.; VAN DEN BERG, E.; FONTES M.A.L. & BOTEZELLI, L. 2005. Distribuição de espécies arbóreo-arbustivas ao longo de um gradiente de solos e topografia em um trecho de floresta ripária do rio São Francisco em Três Marias, MG, Brasil. Revista Brasileira de Botânica 28(2): 329-345. [TreeCo refID: 150]

CARVALHO, E.S.; SANTOS, J.P.A. SILVA, R.R.; MIGUEZ, L.S.; SOUZA, M.O. & MENDONÇA, A.V.R. 2018. Biometria de frutos e sementes de Lecythis lurida (Miers) S.A. Mori. Anais do VII Congresso Florestal Latino-americano, Vitória. https://even3.blob.core.windows.net/anais/95401.pdf [TreeCo refID: 10416]

CARVALHO, G.M. 2011. Influência de processos estocásticos sobre a estruturação de comunidades em Floresta de Tabuleiros Bahia, Brasil. Dissertação (Mestrado), Universidade Estadual de Santa Cruz, Ilhéus. 61p. [TreeCo refID: 2702]

CARVALHO, L.D'A.F. 1988. Revisão Taxonômica das Especies de Solanum das Seções Cernuum e Lepidotum-Solanaceae. Doctoral Thesis, Universidade Estadual de Campinas, Campinas. 228p. [TreeCo refID: 10167]

CARVALHO, P.E.R. 2004. Pau-Marfim – Balfourodendron riedelianum. Embrapa Florestas. Colombo, PR. Circular Técnica n° 93. <http://www.cnpf.embrapa.br/publica/circtec/edicoes/circtec93.pdf > [TreeCo refID: 10017]

CARVALHO, W.A.C. 2002. Variações da composição e estrutura do compartimento arbóreo da vegetação de oito fragmentos de floresta semidecídua do vale do Alto Rio Grande, MG. Dissertação (Mestrado). Universidade Federal de Lavras, Lavras. 168p. [TreeCo refID: 154]

CARVALHO, W.A.C. 2010. Diversidade do estrato arbóreo-arbustivo de sete comunidades de floresta ombrófila altomontanas da APA Fernão Dias, MG, Brasil. Tese (Doutorado). Universidade Federal de Minas Gerais, Belo Horizonte. 72p. [TreeCo refID: 2556]

CATHARINO, E.L.M.; BERNACCI, L.C.; FRANCO, G.A.D.C. & DURIGAN, G.; METZGER, J.P. 2006. Aspectos da composição e diversidade do componente arbóreo das florestas da Reserva Florestal do Morro Grande, Cotia, SP. Biota Neotropica 6(2): ISSN 1806-129X. [TreeCo refID: 965]

CERQUEIRA, R.M. 2005. Florística e estrutura de um fragmento de floresta estacional semidecídua montana no município de Itatiba, SP. Dissertação (Mestrado). Universidade de Campinas, Campinas. 106p. [TreeCo refID: 2810]

CÉSAR, O. & LEITÃO–FILHO, H.F. 1990b. Estudo fitossociológico de mata mesófila semidecídua na Fazenda Barreiro Rico, município de Anhembi, SP. Revista Brasileira de Biologia 50: 443-452. [TreeCo refID: 970]

CHAGAS, A.P. 2014. Ingeae Benth. (Leguminosae-Mimosoideae) no Espírito Santo, Brasil. Dissertação (Mestrado). Universidade Federal de Viçosa, Viçosa. 109p. [TreeCo refID: 10181]

CHAVE, J.; COOMES, D.A.; JANSEN, S.; LEWIS, S.L.; SWENSON, N.G. & ZANNE, A.E. 2009. Towards a worldwide wood economics spectrum. Ecology Letters 12(4): 351-366. (ZANNE, A.E. et al. 2009. Global Wood Density Database. Dryad Digital Repository. https://doi.org/10.5061/dryad.234) [TreeCo refID: 10254]

CHIMELO, J.P.; MAINIERI, C.; HAHUZ, M.A.R.; PESSOA, A.L. 1976.Madeiras do município de Aripuanã, Estado de Mato Grosso. I - Caracterização anatômica e aplicações. Acta Amazonica, 6(4): 95-106 apud PAULA, J.E. & ALVES, J.L.H. 2007. 897 Madeiras nativas do Brasil: anatomia, dendrologia, dendrometria, produção, uso. Editora Cinco Continentes, Porto Alegre. 438p. [TreeCo refID: 10124]

CINTRA, T.C. 2009. Avaliações energéticas de espécies florestais nativas plantadas na região do Médio Paranapanema, SP. Dissertação (Mestrado). Escola Superior de Agricultura "Luiz de Queiroz", Piracicaba. 84p. [TreeCo refID: 10018]

COELHO, R.L.G. 2008. Estudos Taxonómicos em Matayba Aubl. sect. Matayba (Sapindaceae). Dissertação (Mestrado). Universidade Estadual de Campinas, Campinas. 172p. [TreeCo refID: 10019]

COELHO, R.L.G. 2014. CoeEstudos sistemáticos das espécies neotropicais de Allophylus L. (Sapindaceae). Tese (Doutorado). Universidade Estadual de Campinas, Campinas. 479p. [TreeCo refID: 10403]

COELHO, S.; CARDOSO-LEITE, E. & CASTELLO, A.C.D. 2016. Composição florística e caracterização sucessional como subsídio para conservação e manejo do PNMCBio, Sorocaba – SP. Ciência Florestal 26(1): 331-344. [TreeCo refID: 3824]

COLONETTI, S.; CITADINI-ZANETTE, V.; MARTINS, R.; SANTOS, R.D.; ROCHA, E.; & JARENKOW, J.A. 2009. Floristic composition and phytosociological structure in a submontane ombrophilous dense forest at São Bento river dam, Siderópolis, Santa Catarina State. Acta Scientiarum-Biological Sciences 31(4): 397-405. [TreeCo refID: 813]

COSMO, N.L.; NOGUEIRA, A.C.; LIMA, J.G. & KUNIYOSHI, Y.S. 2010. Morfologia de fruto, semente e plântula de Sebastiania commersoniana, Euphorbiaceae. Floresta 40(2): 419-428. [TreeCo refID: 10411]

COSTA-FILHO, S.V.S. 2016. Estrutura, composição e diversidade florística dos estratos verticais de um fragmento de floresta ombrófila mista em Campo do Tenente, Paraná. Monografia (Graduação). Universidade Federal do Paraná, Curitiba. 13 p. [TreeCo refID: 3834]

COSTA, J.C.A. 2004. Fixação de carbono e produção de biomassa pela cupiúva (Tapirira guianensis Aubl.), em um fragmento manejado de mata atlântica, município de Goiana-PE. Dissertação (Mestrado). Universidade Federal Rural do Pernambuco, Recife. 109p. [TreeCo refID: 10020]

COSTA, T.G.; BIANCHI, M.L.; PROTÁSIO, T.P.; TRUGILHO, P.F. & PEREIRA, A.J. 2014. Qualidade da madeira de cinco espécies de ocorrência no cerrado para produção de carvão vegetal. CERNE 20(1): 37-46. [TreeCo refID: 10021]

COWAN, R. 1967. Swartzia (Leguminosae, Caesalpinioideae Swartzieae). Flora Neotropica 1: 1-228. [TreeCo refID: 10435]

CUNHA, M.P.S.C.; PONTES, C.L.F.; CRUZ, I. A.; CABRAL, M.T F.D.; CUNHA NETO, Z.B. & BARBOSA, A.P.R. 1989. Estudo químico de 55 espécies lenhosas para geração de energia em caldeiras. In: 3° encontro Brasileiro em madeiras e em estruturas de madeira: Anais, São Carlos: 2: 93-121. [TreeCo refID: 10022]

DALANESI, P.E.; OLIVEIRA-FILHO, A.T. & FONTES, M.A.L. 2004. Flora e estrutura do componente arbóreo da floresta do Parque Ecológico Quedas do Rio Bonito, Lavras – MG, e correlações entre a distribuição das espécies e variáveis ambientais. Acta Botanica Brasilica 18(4): 737-757. [TreeCo refID: 70]

DALY, D.C. 1990. The genus Tetragastris and the forests of Eastern Brazil: studies in Neotropical Burseraceae III. Kew Bulletin 45(1): 179-194. [TreeCo refID: 10023]

DALY, D.C. 1999. Notes on Trattinnickia, including a Synopsis in Eastern Brazil's Atlantic Forest Complex. Studies in Neotropical Burseraceae IX. Kew Bulletin 54(1): 129-137. [TreeCo refID: 10024]

DAMÁSIO, R.A.P.; PEREIRA, B.L.C.; OLIVEIRA, A.C.; CARDOSO, M.T.; VITAL, B.R. & CARVALHO, A.M.L.M. 2013. Caracterização anatômica e qualidade do carvão vegetal da madeira de pau-jacaré (Piptadenia gonoacantha). Pesquisa Florestal Brasileira 33(75): 261-267. [TreeCo refID: 10025]

DAVIDE, A.C.; TONETTI, O.A.O. & SILVA, E.A.A.D. 2011. Improvement to the physical quality and imbibition pattern in seeds of candeia (Eremanthus incanus (Less.) Less.). CERNE 17(3): 321-326. [TreeCo refID: 10026]

DIAS-NETO, O.C.; SCHIAVINI, I.; LOPES, S.F.; VALE, V.S.; GUSSON, A.E. & OLIVEIRA, A. P. 2009. Estrutura fitossociológica e grupos ecológicos em fragmento de floresta estacional semidecidual, Uberaba, Minas Gerais, Brasil. Rodriguésia 60(4): 1087-1100. [TreeCo refID: 160]

DIAS, M.C. 1988. Estudos taxonomicos do genero Xylopia L. (Annonaceae) no Brasil extra-amazonico. Dissertação (Mestrado). Universidade Estadual de Campinas, Campinas. 183p. [TreeCo refID: 10170]

DUARTE, M.C. 2010. Análise filogenética de Eriotheca Schott & Endl. e gêneros afins (Bombacoideae, Malvaceae) e estudo taxonômico de Eriotheca no Brasil. Tese (Doutorado). Instituto de Botânica da Secretaria de Estado do Meio Ambiente, São Paulo. 190p. [TreeCo refID: 10129]

DUARTE, T.G. 2007. Florística, fitossociologia e relações solo-vegetação em Floresta Estacional Decidual em Barão de Melgaço, Pantanal de Mato Grosso. Tese (Doutorado). Univeridade Federal de Viçosa, Viçosa. 162p. [TreeCo refID: 1959]

DURIGAN, G.; BAITELLO, J.B.; FRANCO, G.A.D.C. & SIQUEIRA, M.F. 2004. Plantas do Cerrado Paulista: imagens de uma paisagem ameaçada. Ed. Páginas e Letras, São Paulo. 475p. [TreeCo refID: 10158]

ÉDER-SILVA, E. & ARAÚJO, D.R. 2014. Physiological quality, morphometric aspects and chromosome number of the species Talisia esculenta Radlk. Revista Verde de Agroecologia e Desenvolvimento Sustentável 9(3):275-82. [TreeCo refID: 10413]

ESPÍRITO-SANTO, F.D.B.; OLIVEIRA-FILHO, A.T.; MACHADO, E.L.M.; SOUZA, J.S.; FONTES, M.A.L. & MARQUES, J.J.G.S.M. 2002. Variáveis ambientais e a distribuição de espécies arbóreas em um remanescente de floresta estacional semidecídua montana no campus da Universidade Federal de Lavras, MG. Acta Botanica Brasilica 16(3): 331-356. [TreeCo refID: 161]

FAGUNDES, L.M.; CARVALHO, D.A.; VAN DEN BERG, E.; MARQUES, J.J.G.S.M. & MACHADO, E.L.M. 2007. Florística e estrutura do estrato arbóreo de dois fragmentos de florestas decíduas às margens do rio Grande, em Alpinópolis e Passos, MG. Acta Botanica Brasilica 21(1): 65-78. [TreeCo refID: 108]

FARAH, F.T. 2009. Vinte anos de dinâmica em um hectare de Floresta Estacional Semidecidual. Tese (Doutorado). Universidade Estadual de Campinas, Campinas. 130p. [TreeCo refID: 986]

FARIA JUNIOR, J.E.Q. 2010. O gênero Eugenia L. (Myrtaceae) nos estados de Goiás e Tocantins, Brasil. Dissertação (Mestrado). Universidade de Brasília, Brasília. 266p. [TreeCo refID: 10031]

FARIA JUNIOR, J.E.Q. 2014. Revisão Taxonômica e Filogenia de Eugenia sect. Pilothecium (Kiaersk) D.Legrand (Myrtaceae). Tese (Doutorado). Universidade de Brasília, Brasília. 215p. [TreeCo refID: 10032]

FERNANDES, I. 1997. Taxonomia e fitogeografia de Cyatheaceae e Dicksoniaceae nas Regiões Sul e Sudeste do Brasil. Tese (Doutorado). Universidade de São Paulo, São Paulo. 435p. [TreeCo refID: 10033]

FERREIRA JUNIOR, M. & VIEIRA, A.O.S. 2015. Tree and shrub Rubiaceae Juss. family species along Tibagi river basin, Paraná State, Brazil. Hoehnea 42(2): 289-336. [TreeCo refID: 10034]

FERREIRA, P.I.; GOMES, J.P.; BATISTA, F.; BERNARDI, A.P.; COSTA, N.C.F.D.; BORTOLUZZI, R.L.D.C. & MANTOVANI, A. 2013. Potential species for recovery of permanent preservation areas in the highlands of Santa Catarina state, Brazil. Floresta e Ambiente 20(2): 173-182. [TreeCo refID: 814]

FIASCHI, P. & PIRANI, J.R. 2007. Estudo taxonômico do gênero Schefflera J.R. Forst. & G. Forst (Araliaceae) na região sudeste do Brasil. Boletim de Botânica da Universidade de São Paulo 25: 95-142. [TreeCo refID: 10163]

FIGLIOLA, M.B. & PINA-RODRIGUES, F.C.M. 1995. Manejo de sementes de espécies arbóreas. IF. Serie Registros 15: 1-59. [TreeCo refID: 10035]

FIGUEIREDO FILHO, A.; ORELLANA, E.; NASCIMENTO, F.; DIAS, A.N. & INOUE, M.T. 2011. Produção de sementes de Araucaria angustifolia em plantio e em floresta natural no Centro-sul do Estado do Paraná. Floresta 41(1): 155-162. [TreeCo refID: 10405]

FONSECA, C.R. & CARVALHO, F.A. 2012. Aspectos florísticos e fitossociológicos da comunidade arbórea de um fragmento urbano de floresta Atlântica (Juiz de Fora, MG, Brasil). Bioscience Journal 28(5): 820-832. [TreeCo refID: 116]

FONTES, M.A.L. 2008. Dinâmica de comunidades arbóreas de florestas alti-montanhas de Minas Gerais. Tese (Doutorado). Universidade Federal de Minas Gerais, Belo Horizonte. 85p. [TreeCo refID: 3597]

FORERO, E. 1983. Connaraceae. Flora Neotropica 36: 1-207. [TreeCo refID: 10135]

FORGIARINI, C.; SOUZA, A.F.; LONGHI, S.J. & OLIVEIRA, J.M. 2014. In the lack of extreme pioneers: trait relationships and ecological strategies of 66 subtropical tree species. Journal of Plant Ecology 8(4): 359-367. [TreeCo refID: 10037]

FRANÇA, G.S. & STEHMANN, J.R. 2004. Composição florística e estrutura de componente arbóreo de uma floresta altimontana no município de Camanducaia, Minas Gerais, Brasil. Revista Brasileira de Botânica 27 (1): 19-30. [TreeCo refID: 71]

FRANÇA, M.B. 2002. Modelagem de biomassa florestal através do padrão espectral no Sudoeste da Amazônia. Dissertação (Mestrado). Instituto Nacional de Pesquisas da Amazônia/Fundaçãoo Universidade Federal do Amazonas, Manaus, Amazonas. 106p. [TreeCo refID: 10038]

FREITAS, M.F. & KINOSHITA, L.S. 2015. Myrsine (Myrsinoideae-Primulaceae) no sudeste e sul do Brasil. Rodriguésia 66(1): 167-189. [TreeCo refID: 10187]

FURLAN, A. & GIULIETTI, A.N. 2014. A tribo Pisonieae Meisner (Nyctaginaceae) no Brasil. Boletim de Botânica 32(2): 145-268. [TreeCo refID: 10039]

GALETTI, M.; PIZO, M.A. & MORELLATO, L.P.C. 2011. Diversity of functional traits of fleshy fruits in a species-rich Atlantic rain forest. Biota Neotropica 11(1): 181-193. [TreeCo refID: 10040]

GANDOLFI, S. 2000. História Natural de uma floresta estacional semidecidual no município de Campinas (São Paulo, Brasil). Tese (Doutorado). Universidade de Campinas, Campinas. 520p. [TreeCo refID: 2631]

GENTRY, A. 1992. Bignoniaceae: Part II (Tribe Tecomeae). Flora Neotropica 25(2): 1-370. [TreeCo refID: 10436]

GERMANO FILHO, P. 1999. Estudos Taxonômicos do gênero Bathysa C.Presl (Rubiaceae, Rondeletieae), no Brasil.Rodriguésia 50(76/77): 49-75. [TreeCo refID: 10171]

GIBBS, P.E. & SEMIR, J. 2003. A taxonomic revision of the genus Ceiba Mill. (Bombacaceae). Anales Jardín Botánico de Madrid 60: 259-300. [TreeCo refID: 10172]

GOLDENBERG, R. 2004. O gênero Miconia (Melastomataceae) no Estado do Paraná, Brasil. Acta bot. Bras. 18(4): 927-947. [TreeCo refID: 10401]

GOMES, E.P.C.; FISCH, S.T.V. & MANTOVANI, W. 2005. Estrutura e composição do componente arbóreo na Reserva Ecológica do Trabiju, Pindamonhangaba, SP, Brasil. Acta Botanica Brasilica 19(3): 451-464. [TreeCo refID: 997]

GONÇALVES, C.A., LELIS, R.C.C. & ABREU, H.S. 2010. Caracterização físico-química da madeira de sabiá (Mimosa caesalpiniaefolia Benth.).Revista Caatinga 23(1): 54-62. [TreeCo refID: 10041]

GOODMAN, R.C.; PHILLIPS, O.L.; CASTILLO TORRES, D. del.; FREITAS, L.; TAPIA CORTESE, S.; MONTEAGUDO, A. & BAKER, T.R. 2013. Amazon palm biomass and allometry. Forest Ecology and Management 310: 994 - 1004. [TreeCo refID: 10093]

GREEN, P.S. 1994. A Revision of Chionanthus (Oleaceae) in S. America and the Description of Priogymnanthus, gen. nov. Kew Bulletin 49(2): 261-286. [TreeCo refID: 10042]

GROMBONE-GUARATINI, M.T.; GOMES, E.P.C.; TAMASHIRO, J.Y & RODRIGUES, R.R. 2008. Composição florística da Reserva Municipal de Santa Genebra, Campinas, SP. Revista Brasileira de Botânica 31(2): 323-337. [TreeCo refID: 999]

GUIMARÃES, P.J.F. 1997. Estudos taxonômicos de Tibouchina sect. Pleroma (D. Don) Cogn. (Melastomastaceae). Tese (Doutorado). Universidade Estadual de Campinas, São Paulo. 191p. [TreeCo refID: 10043]

GUSSON, A.E.; LOPES, S.F.L.; DIAS NETO, O.C.; VALE, V.S.; OLIVEIRA, A.P. & SCHIAVINI, I. 2009. Características químicas do solo e estrutura de um fragmento de floresta estacional semidecidual em Ipiaçu, Minas Gerais, Brasil. Rodriguésia 60(2): 403-414. [TreeCo refID: 178]

HAIDAR, R.F. 2008. Fitossociologia, diversidade e sua relação com variáveis ambientais em florestas estacionais do Bioma Cerrado no Planalto Central e Nordeste do Brasil. Dissertação (Mestrado). Universidade de Brasília, Brasília, DF, Brasil. 255p. [TreeCo refID: 1256]

HAIDAR, R.F.; FELFILI, J.M.; PINTO, J.R.R. & FAGG, C.W. 2005. Fitossociologia da vegetação arbórea em fragmentos de floresta estacional, no Parque Ecológico Altamiro de Moura Pacheco, GO. Boletim do Herbário Ezechias Paulo Heringer 15: 19-46. [TreeCo refID: 2658]

HAMILTON, C.W. 1989. A revision of mesoamerican Psychotria subgenus Psychotria (Rubiaceae), part I: introduction and species 1-16. Annals of the Missouri Botanical Garden 76(1): 67-111. [TreeCo refID: 10044]

HAYDEN, S.M. & HAYDEN, W.J. 1996. A revision of Discocarpus (Euphorbiaceae). Annals of the Missouri Botanical Garden 83(2): 153-167. [TreeCo refID: 10045]

HERTZOG A.; PELLEGRINI, M.O.O. & SANTOS-SILVA F. 2016. Winteraceae do Rio Grande do Sul, Brasil. Rodriguésia 67(1): 251–260. [TreeCo refID: 10192]

IMAÑA-ENCINAS, J.; PAULA, J.E. & CONCEIÇÃO, C.A. 2012. Florística, volume e biomassa lenhosa de um fragmento de Mata Atlântica no municipio de Santa Maria de Jetibá, Espirito Santo. Floresta 42(3): 565 - 576. [TreeCo refID: 10046]

Inventário Florestal de Minas Gerais (Unpublished data). Literature compilation of species traits provided by the researchers from Minas Gerais state inventory. Universidade Federal de Lavras, Lavras. [TreeCo refID: 10427]

Inventário Florestal de Minas Gerais (Unpublished data). Primary data of maximum tree height and maximum diameter at breast height from the Minas Gerais state inventory. Universidade Federal de Lavras, Lavras. [TreeCo refID: 10426]

ISACKSSON, J.G.L. 2015. Árvores, propágulos e plântulas de duas Licania spp. (Chrysobalanaceae) nativas da floresta de várzea estuarina, Amapá. Monografia (Graduação). Universidade do Estado do Amapá, Macapá. 72p. [TreeCo refID: 10415]

ISACKSSON, J.G.L. 2018. Morfologia de frutos, sementes e plântulas de espécies neotropicais de Chrysobalanaceae como suporte filogenético. Dissertação (Mestrado). Instituto Nacional de Pesquisas da Amazônia, Manaus. 145p. [TreeCo refID: 10412]

IZA, O.B. 2002. Parâmetros de autoecologia de uma comunidade arbórea de Floresta Ombrófila Densa, no Parque Botânico do Morro Baú, Ilhota, SC. Dissertação (Mestrado). Universidade Federal de Santa Catarina, Florianópolis. 92p. [TreeCo refID: 842]

JORDANO, P. 1995. Angiosperm fleshy fruits and seed dispersers: a comparative analysis of adaptation and constraints in plant-animal interactions. American Naturalist 145(2): 163-191. (JORDANO, P. 2013. FRUBASE dataset. Version 3.0. Dryad Digital Repository. https://doi.org/10.5061/dryad.9tb73/1) [TreeCo refID: 10314]

KAASTRA, R.C. 1982. Pilocarpinae (Rutaceae). Flora Neotropica 33: 1-197. [TreeCo refID: 10437]

KILCA, R.V.; SOARES, J.C.W.; MEDEIROS, E.M. & JARENKOW, J.A. 2012. Cambios florísticos y estructurales entre dos comunidades arbóreas de un bosque ripario bajo condiciones ambientales contrastantes en la Pampa sur brasileña. Iheringia série Botânica 67(2):165-175. [TreeCo refID: 1152]

KLEIN, R.M. 1990. Espécies raras ou ameaçadas de Extinção do Estado de Santa Catarina. Vol. 2. IBGE, Rio de Janeiro. 287 p. [TreeCo refID: 10049]

KNAPP, S. 2002. Solanum Section Geminata (Solanaceae). Flora Neotropica 84: 1-404. [TreeCo refID: 10154]

KUBITZKI, K. & RENNER, S. 1982. Lauraceae I (Aniba and Aiouea). Flora Neotropica 31: 1-124. [TreeCo refID: 10134]

KURTZ, B.C. & ARAUJO, D.S.D. 2000. Composição florística e estrutura do componente arbóreo de um trecho de Mata Atlântica na Estação Ecológica Estadual do Paraíso, Cachoeiras de Macacu, Rio de Janeiro, Brasil. Rodriguesia 51(78/115): 69-112. [TreeCo refID: 531]

LANDRUM, L. 1981. A Monograph of the Genus Myrceugenia (Myrtaceae). Flora Neotropica 29: 1-135. [TreeCo refID: 10141]

LANDRUM, L. 1986. Campomanesia, Pimenta, Blepharocalyx, Legrandia, Acca, Myrrhinium, and Luma (Myrtaceae). Flora Neotropica 45: 1-178. [TreeCo refID: 10155]

LEAL, T.DE.S. 2015. Florística e fitossociologia de Cerrado Sentido Restrito em regeneração natural no município de Pirassununga, estado de São Paulo. Dissertação (Mestrado). Universidade Estadual Paulista "Júlio de Mesquita Filho", Rio Claro. 66p. [TreeCo refID: 3348]

LEITÃO FILHO, H.F. 1972. Contribuição ao conhecimento taxonômico da Tribo Vernonieae no Estado de Sãoo Paulo. Tese (Doutorado). Escola Superior de Agricultura "Luiz de Queiroz", Piracicaba. 217p. [TreeCo refID: 10050]

LERF (Unpublished data). Compilation of species traits from the literature from the Laboratório de Ecologia e Restauração Florestal. ESALQ/USP, Piracicaba. (http://www.lerf.eco.br) [TreeCo refID: 10417]

LIMA JUNIOR, M.J.V. (ed). 2010. Manual de Procedimentos para Análise de Sementes Florestais. Universidade Federal do Amazonas, Manaus. 146p. [TreeCo refID: 10053]

LIMA, A.L.A. & RODAL, M.J.N. 2010. Phenology and wood density of plants growing in the semi-arid region of northeastern Brazil.Journal of Arid Environments 74(11): 1363-1373. [TreeCo refID: 10051]

LIMA, H.C. 1986. Tribo Dalbergieae (Leguminosae - Papilionoideae) em estudo morfológico dos frutos, sementes e plântulas e sua aplicação na sistemática. Dissertação (Mestrado). Universidade Federal do Rio de Janeiro, Rio de Janeiro. 127p. [TreeCo refID: 10052]

LIMA, J.A.S. 2009. Biomassa arbórea e estoques de nutrientes em fragmentos florestais da APA Rio São João: o efeito da fragmentação sobre a Mata Atlântica da Baixada Litorânea Fluminense. Tese (Doutorado). Universidade Estadual do Norte Fluminense, Campo dos Goytacazes. 180p. [TreeCo refID: 3028]

LIMA, M.E.L.; CORDEIRO, I. & MORENO, P.R.H. 2011. Estrutura do componente arbóreo em Floresta Ombrófila Densa Montana no Parque Natural Municipal Nascentes de Paranapiacaba (PNMNP), Santo André, SP, Brasil. Hoehnea 38(1): 73-96. [TreeCo refID: 894]

LINDENMAIER, D.S. & BUDKE, J.C. 2006. Florística, diversidade e distribuição espacial das espécies arbóreas em uma floresta estacional na bacia do rio Jacuí, sul do Brasil. Instituto Anchietano de Pesquisas Botânica 57: 193-216. [TreeCo refID: 638]

LINGNER, D.V.; SCHORN, L.A.; VIBRANS, A.C.; MEYER, L.; SEVEGNANI, L.; GASPER, A.L.; SOBRAL, M.G.; KRÜGER, A.; KLEMZ, G.; SCHMIDT, R. & ANASTÁCIO-

JUNIOR, C. 2013. Fitossociologia do componente arbóreo/arbustivo da floresta ombrófila densa em Santa Catarina. In: VIBRANS, A.C.; SEVEGNANI, L.; GASPER, A.L. & LINGNER, D.V. (eds.) Inventário Florístico Florestal de Santa Catarina, Vol. IV, Floresta Ombrófila Densa. Edifurb, Blumenau. 159-200. | MEYER, L.L.; SEVEGNANI, A.L.G.; SCHORN, L.A.; VIBRANS, A.C.; LINGNER, D.V.; SOBRAL, M.; KLEMZ, G.; SCHMITT, R.; ANASTACIO-JR, C. & BROGNI, E. 2013. Fitossociologia do componente arbóreo/arbustivo da Floresta Ombrófila Mista em Santa Catarina. In: VIBRANS, A.C.; SEVGNANI, L.; GASPER, A.L. & LINGNER, D.V. (orgs.) Inventário florístico florestal de Santa Catarina (IFFSC): Floresta Ombrófila Mista. Edifurb, Blumenau. 157-189. | SCHORN, L.A.; LINGNER, D.V.; VIBRANS, A.C.; GASPER, A.L.; SEVEGNANI, L.; SOBRAL, M.; MEYER, L.; KLEMZ, G.; SCHMITT, R.; ANASTACIO-JR, C.; PASQUALLI, V.R. 2013. Estrutura do componente arbóreo/arbustivo da Floresta Estacional Decidual em Santa Catarina. In: VIBRANS, A. C.; SEVGNANI, L.; GASPER, A. L.; LINGNER, D. V. (eds.). Inventário florístico florestal de Santa Catarina (IFFSC): Floresta Estacional Semidecidual. Edifurb, Blumenau. Vol. 2. 142-163. [TreeCo refID: 2394|2668|2773]

LOBÃO, A.Q. 2009. Filogenia de Guatteria (Annonaceae) e revisão taxonômica das espécies da Floresta Atlântica. Tese (Doutorado). Jardim Botânico do Rio de Janeiro, Rio de Janeiro. 156p. [TreeCo refID: 10054]

LOPES, J.DE.C. & MELLO-SILVA, R. 2012. Annonaceae do Parque Estadual de Ibitipoca, Minas Gerais. Boletim de Botânica 30(2): 157-164. [TreeCo refID: 10055]

LOPES, S.D.F.; SCHIAVINI, I.; PRADO-JÚNIOR, J.A.; GUSSON, A.E.; SOUZA-NETO, A.R.; VALE, V.S. & DIAS-NETO, O.C. 2011. Ecological characterization and diametric distribution of arboreal vegetation in remanescent of seasonal semideciduous forest gloria's experimental farm, Uberlandia, MG. Bioscience Journal 27(2): 322-335. [TreeCo refID: 89]

LOREGIAN, A.C.; SILVA, B.B.; ZANIN, E.M.; DECIAN, V.S.; HENKE-OLIVEIRA, C. & BUDKE, J.C. 2012. Padrões espaciais e ecológicos de espécies arbóreas refletem a estrutura em mosaicos de uma floresta subtropical. Acta Botanica Brasilica 26(3): 593-606. [TreeCo refID: 639]

LORENZI, H. 1992. Árvores brasileiras: manual de identificação e cultivo de plantas arbóreas nativas do Brasil, Vol. 1. Editora Plantarum, Nova Odessa. 384p. [TreeCo refID: 10057]

LORENZI, H. 1998. Árvores brasileiras: manual de identificação e cultivo de plantas arbóreas nativas do Brasil, Vol. 2. Editora Plantarum, Nova Odessa. 351p. [TreeCo refID: 10058]

LORENZI, H. 2009. Árvores brasileiras: manual de identificação e cultivo de plantas arbóreas nativas do Brasil, Vol. 3. Editora Plantarum, Nova Odessa. 384p. [TreeCo refID: 10059]

LORENZI, H.; NOBLICK, L.R.; KAHN, F. & FERREIRA, E. 2010. Flora brasileira: Arecaceae (Palmeiras). Instituto Plantarum, Nova Odessa. 382p. [TreeCo refID: 10056]

MAAS, P.J.M.; WESTRA, L.Y.T. & CHATROU, L.W. 2003. Duguetia (Annonaceae). Flora Neotropica Monograph 88: 1-274. [TreeCo refID: 10060]

MAAS, P.J.M.; WESTRA, L.Y.TH.; BROWN, K.S.; MAAS, P.; TER WELLE, B.J.H.; WEBBER, A.C.; LE THOMAS, A.; WAHA, M.; VAN DER HEIJDEN, E.; BOUMAN, F.;

CAVÉ, A.; LEBOEUF, M.; LAPRÉVOTE, O.; KOEK-NOORMAN, J.; MORAWETZ, W. & HEMMER, W. 1992. Rollinia. Flora Neotropica 57: 1-188. [TreeCo refID: 10137]

MAÇANEIRO, J.P.; SEUBERT, R.C. & SCHORN, L.A. 2015. Fitossociologia de uma Floresta Pluvial Subtropical primária no sul do Brasil. Floresta 45(3): 555-566. [TreeCo refID: 3453]

MAGNAGO, L.F.S. (Unpublished data). Measurements and compilations of fruits and seed traits performed by Luiz F. Silva Magnago. Universidade Federal do Sul da Bahia, Itabuna. [TreeCo refID: 10421]

MAGNAGO, L.F.S. 2009. Gradiente vegetacional e pedológico em mata de restinga no estado do Espírito Santo. Dissertação (Mestrado). Universidade Federal de Viçosa, Viçosa. 122p. [TreeCo refID: 24]

MANSANO, V.F. & LIMA, J.R. 2007. O gênero Swartzia Schreb. (Leguminosae, Papilionoideae) no estado do Rio de Janeiro. Rodriguésia 58(2): 469-483. [TreeCo refID: 10183]

MANTOVANI, A.; MORELLATTO, L.P.C. & REIS, M.S. 2004. Fenologia reprodutiva e produção de sementes em Araucaria angustifolia (Bertol.) Kuntze. Revista Brasileira de Botânica 27(4): 787-796. [TreeCo refID: 10407]

MANTOVANI, M.; RUSCHEL, A.R.; PUCHALSKI, Â.; SILVA, J.Z.; REIS, M.S. & NODARI, R.O. 2005. Diversidade de espécies e estrutura sucessional de uma formação secundária da floresta ombrófila densa. Scientia Forestalis 67(1): 14-26. [TreeCo refID: 830]

MANZATTO, A.G.; FURLAN, A.; CESAR, O. & PAGANO, S.N. 1999. Vegetação lenhosa do SESC Interlagos, São Paulo, SP. Universidade Estadual Paulista "Júlio de Mesquista Filho", Rio Claro. 74p. [TreeCo refID: 600]

MARCHIORI, J.N.C. 1997. Dendrologia das angiospermas: das magnoliáceas às flacurtiáceas. Editoria da Universidade Federal de Santa Maria, Santa Maria. 271p. [TreeCo refID: 10127]

MARCHIORI, J.N.C. 1997. Dendrologia das angiospermas: Myrtales. Editoria da Universidade Federal de Santa Maria, Santa Maria. 304p. [TreeCo refID: 10128]

MARCHIORI, J.N.C. 2000. Dendrologia das angiospermas: das bixáceas às rosáceas. Editora da Universidade Federal de Santa Maria, Santa Maria.240p. [TreeCo refID: 10126]

MARCHIORI, N.M.; ROCHA, H.R.; TAMASHIRO, J.Y. & AIDAR, M.P.M. 2016. Composição da comunidade arbórea e biomassa aérea em uma floresta atlântica secundária, Parque Estadual da Serra do Mar, São Paulo, Brazil. CERNE 22(4): 501-514. [TreeCo refID: 3626]

MARÇON, S.L. 2009. Composição florística e estrutura do componente arbustivo-arbóreo do Parque Natural Municipal da Cratera da Colônia, São Paulo, SP. Dissertação (Mestrado). Universidade de São Paulo, Ribeirão Preto. 120p. [TreeCo refID: 1020]

MARCONDES-FERREIRA NETO, W. 1988. Aspidosperma Mart., nom. cons. (Apocynaceae) : estudos taxonomicos. Tese (Doutorado). Universidade Estadual de Campinas, Campinas. 431p. [TreeCo refID: 10402]

MARQUES, S.D.S.; OLIVEIRA, J.D.S.; PAES, J.B.; ALVES, E.S.; SILVA, A. & FIEDLER, N.C. 2012. Estudo comparativo da massa específica aparente e retratibilidade da madeira de

pau-brasil (Caesalpinia echinata Lam.) nativa e de reflorestamento. Revista Árvore 36(2): 373-380. [TreeCo refID: 10063]

MARTINEZ-YRIZAR, A.; SARUKHAN, J.; PEREZ-JIMENEZ, A.; RINCON, E.; MAASS, J.M.; SOLIS-MAGALLANES, A. & CERVANTES L. 1992. Above-Ground Phytomass of a Tropical Deciduous Forest on the Coast of Jalisco, Mexico. Journal of Tropical Ecology 8(1): 87-96. [TreeCo refID: 10064]

MARTINI, A.M.Z.; FIASCHI, P.; AMORIM, A.M. & PAIXÃO, J.L. 2007. A hot-point within a hot-spot: a high diversity site in Brazil's Atlantic Forest. Biodiversity and Conservation 16(11) :3111-3128. [TreeCo refID: 10065]

MARTINS, F.R. 1991. Estrutura de uma floresta mesófila. Editora da UNICAMP, Campinas. 214 p. [TreeCo refID: 1022]

MARTINS, L.T. 2012. Caracterização dendrométrica e crescimento de dez espécies florestais nativas em plantios homogêneos no estado do Espírito Santo. Dissertação (Mestrado). Universidade Federal do Espírito Santo, Jerônimo Monteiro. 102p. [TreeCo refID: 10066]

MARTINS, R. 2005. Florística, estrutura fitossociológica e interações interespecíficas de um remanescente de floresta ombrófila densa como subsídio para a recuperação de áreas degradadas pela mineração de carvão, Siderópolis, SC. Dissertação (Mestrado). Universidade Federal de Santa Catarina, Florianópolis. 93p. [TreeCo refID: 2572]

MATOS, M.Q. & FELFILI, J.M. 2010. Florística, fitossociologia e diversidade da vegetação arbórea nas matas de galeria do Parque Nacional de Sete Cidades (PNSC), Piauí, Brasil. Acta Botanica Brasilica 24(2): 483-496. [TreeCo refID: 1589]

MAZINE, F.F. 2002. Estudo taxonômico das espécies de Myrtaceae ocorrentes nos campos de altitude do Parque Nacional do Caparaó (ES/MG). Dissertação (Mestrado). Universidade de São Paulo, São Paulo. 77p. [TreeCo refID: 10067]

MAZINE, F.F. 2006. Estudos taxonomicos em Eugenia L. (Myrtaceae), com enfase em Eugenia secrt. Racemosa O. Berg. Tese (Doutorado). Universidade de São Paulo, São Paulo. 239p. [TreeCo refID: 10068]

MEDEIROS, M.B & WALTER, B.M.T. 2012. Composição e estrutura de comunidades arbóreas de Cerrado sensu stricto no norte do Tocantins e sul do Maranhão. Revista Árvore 36(4): 673-683. [TreeCo refID: 1900]

MEDEIROS, M.B; WALTER, B.M.T. & SILVA, G.P. 2008. Fitossociologia do Cerrado sensu stricto no município de Carolina, Maranhão, Brasil. CERNE 14(4): 285-294. [TreeCo refID: 1894]

MEIFA, M.N.E. & CASTILLO, M.U. 1992. Poder calorífico de cinco espécies de Bombacaceas. Revista Florestal Del Peru 19(1):93-97. [TreeCo refID: 10069]

MEIRELLES, A.C. & SOUZA, L.A.G. 2015. Germinação natural de oito espécies de Swartzia (Fabaceae, Faboideae) da Amazônia. Scientia Amazonia 4(3): 84-92. [TreeCo refID: 10408]

MEIRELLES, J. 2015. Filogenia de Miconia seção Miconia subseção Seriatiflorae e revisão taxonômica do clado Albicans (Melastomataceae, Miconieae). Universidade Estadual de Campinas, Campinas. 219p. [TreeCo refID: 10185]

MELLO-SILVA, R.; LOPES, J.C. & PIRANI, J.R. 2012. Flora da Serra do Cipó, Minas Gerais: Annonaceae. Boletim de Botânica 30(1): 23-35. [TreeCo refID: 10027]

MELO, E. 1996. Levantamento das espécies de Coccoloba (Polygonaceae) da restinga do Estado da Bahia, Brasil. Sitientibus 15: 49-59. [TreeCo refID: 10028]

MELO, E. 1999. Levantamento da família Polygonaceae no estado da Bahia, Brasil: espécies do semi-árido. Rodriguésia 50 (76-77): 29-47. [TreeCo refID: 10030]

MELO, M.M.R.F.; BARROS, F.; CHIEA, S.A.C.; KIRIZAWA, M.; JUNG-MENDAÇOLLI, S.L. & WANDERLEY, M.G.L. (eds.). 2009. Flora Fanerogâmica da Ilha do Cardoso. Vol.14. Instituto de Botânica de São Paulo, São Paulo. 118p. [TreeCo refID: 10131]

MELO, M.M.R.F.; BARROS, F.; CHIEA, S.A.C.; KIRIZAWA, M.; JUNG-MENDAÇOLLI, S.L. & WANDERLEY, M.G.L. (org.). 2008. Flora Fanerogâmica da Ilha do Cardoso. Vol.13. Instituto de Botânica, São Paulo. 143p. [TreeCo refID: 10130]

MENDONÇA FILHO, C.V.; TOZZI, A.M.G.A. & MARTINS, E.R.F. 2007. Revisão taxonômica de Machaerium sect. Oblonga (Benth.) Taub. (Leguminosae, Papilionoideae, Dalbergieae). Rodriguésia 58(2):283-312. [TreeCo refID: 10161]

MENDONÇA, N.T. 2005. Florística e fitossociologia em fragmento de Mata Atlântica – Serra da Bananeira, Estação Ecológica de Murici, Alagoas. Dissertação (Mestrado). Universidade Federal Rural de Pernambuco, Recife. 83p. [TreeCo refID: 2637]

MISSIO, F.F.; SILVA, A.C.; HIGUCHI, P.; LONGHI, S.J.; BRAND, M.A.; RIOS, P.D.; DALLA ROSA, A.; BUZZI JUNIOR, F.; BENTO, M.A.; GONÇALVES, D.A.; LOEBENS, R. & PSCHEIDT, F. 2017 Atributos funcionais de espécies arbóreas em um fragmento de Floresta Ombrófila Mista em Lages, SC. Ciência Florestal 27: 215-224. [TreeCo refID: 10070]

MITCHELL, J.D. & DALY, D.C. 1991. Cyrtocarpa Kunth (Anacardiaceae) in South America. Annals of the Missouri Botanical Garden 78(1): 184-189. [TreeCo refID: 10071]

MORAES, P.L.R. 2007. Taxonomy of Cryptocarya species of Brazil (Vol. 3). Belgian Focal Point to the Global Taxonomy Initiative-Royal Belgian Institute of Natural Sciences. 191p. [TreeCo refID: 10400]

MOREAU, J.S. 2014. Estrutura e interação entre vegetação e ambiente de uma Floresta Ombrófila Densa das Terras Baixas, Espírito Santo. Dissertação (Mestrado). Universidade Federal do Espírito Santo, Jerônimo Monteiro. 96p. [TreeCo refID: 2890]

NASCIMENTO, A.R.T.; FELFILI, J.M. & MEIRELLES, E.M. 2004. Florística e estrutura da comunidade arbórea de um remanescente de Floresta Estacional Decidual de encosta, Monte Alegre, GO, Brasil. Acta Botanica Brasilica 18(3): 659-669. [TreeCo refID: 1251]

NEGRELLE, R.R.B. 2006. Composição florística e estrutura vertical de um trecho de Floresta Ombrófila Densa de planície quaternária. Hoehnea 33(3): 261-289. [TreeCo refID: 831]

NEGRELLE, R.R.B. 2013. Tree Species Composition and Estructure of A Remnant of A Semidecidual Seasonal Alluvial Forest Remnant in Pantanal Matogrossense, Brazil. Revista Arvore 37(6): 989-999. [TreeCo refID: 3079]

NÓBREGA, M.G.G.; RAMOS, A.E. & SILVA JUNIOR, M.C. 2001. Composição florísitica e estrutura na mata de galeria do Cabeça de Veado, no Jardim Botânico de Brasília, FDF. Boletim do Herbário Ezechias Paulo Heringer 8: 44-65. [TreeCo refID: 1264]

NOGUEIRA JÚNIOR, L.R. 2010.Estoque de carbono na fitomassa e mudanças nos atributos do solo em diferentes modelos de restauração da Mata Atlântica. Tese (Doutorado). Escola Superior de Agricultura "Luiz de Queiroz", Piracicaba. 94 p. [TreeCo refID: 10072]

NOGUEIRA, E.M. 2008. Wood density and tree allometry in forests of Brazil's 'arc of deforestation' implications for biomass and emission of carbon from land-use change in Brazilian Amazonia. Tese (Doutorado). INPA/UFAM, Manaus. 130p. [TreeCo refID: 10073]

NUNES, E.S.; GUIMARÃES JÚNIOR, J.B.; OLIVEIRA, R.J. & GUIMARÃES NETO, R.M. 2012. determinação da densidade básica da madeira de Qualea parviflora Mart. e Qualea grandiflora Mart.(pau-terra) para produção de carvão vegetal. XXI Seminário de Iniciação Científica IV Seminário em Desenvolvimento Tecnológico e Inovação/ Teresina (PI), 24 a 26 de Outubro de 2012. Resumo expandido. ISSN: 1518-7772. [TreeCo refID: 10074]

OLIVEIRA-FILHO, A.T. 2017. NeoTropTree, Flora arbórea da Região Neotropical: Um banco de dados envolvendo biogeografia, diversidade e conservação. Universidade Federal de Minas Gerais. <http://www.neotroptree,info> Acessado: abril, 2019. [TreeCo refID: 10156]

OLIVEIRA-FILHO, A.T. TreeAtlan 2.0, Flora arbórea da América do Sul cisandina tropical e subtropical: Um banco de dados envolvendo biogeografia, diversidade e conservação. Universidade Federal de Minas Gerais. Disponivel em: <http://www.icb.ufmg.br/treeatlan/>. Acessodo: 2013. [TreeCo refID: 10190]

OLIVEIRA-FILHO, A.T.; CARVALHO, D.A.; FONTES, M.A.L.; Van Den BERG, E.; CURI, N. & CARVALHO, W.A.C. 2004. Variações estruturais do compartimento arbóreo de uma floresta semidecídua alto-montana na chapada das Perdizes, Carrancas, MG. Revista Brasileira de Botânica 27(2): 291-309. [TreeCo refID: 219]

OLIVEIRA-FILHO, A.T.; VILELA, E.A.; GAVILANES, M.L. & CARVALHO, D.A. 1994e. Effect of flooding regime and understorey bamboos on the physiognomy and tree species composition of a tropical semideciduous forest in Southeastern Brazil. Vegetatio 113(2): 99-124. [TreeCo refID: 280]

OLIVEIRA, A.M. 2011. Caracterizaçãoo de uma comunidade de árvores e sua infestação por lianas em uma floresta decídua. Dissertação (Mestrado). Universidade Estadual Paulista "Júlio de Mesquita Filho", Botucatu. 99p. [TreeCo refID: 10075]

OLIVEIRA, G.M.V. 2014. Densidade da madeira em Minas Gerais: amostragem, espacialização e relação com variáveis ambientais. Tese (Doutorado). Universidade Federal de Lavras, Lavras. 125p. [TreeCo refID: 10076]

OLIVEIRA, M.M.A. 1999. Frugivoria por aves em um fragmento de floresta de restinga no estado do Espírito Santo, Brasil. Tese (Doutorado), Universidade Estadual de Campinas, Campinas. 153p. [TreeCo refID: 10077]

PASETTO, M.R. 2008. Composição florística e estrutura de fragmento de Floresta Ombrófila Densa Submontana no município de Siderópolis, Santa Catarina. Trabalho de Conclusão de Curso (Graduação). Universidade do Extremo Sul Catarinense - UNESC, Criciúma. 44p. [TreeCo refID: 2715]

PAULA, A. & SOARES, J.J. 2010. Estrutura horizontal de um trecho de floresta ombrófila densa das terras baixas na Reserva Biológica de Sooretama, Linhares, ES. Floresta 41(2): 321-334. [TreeCo refID: 8]

PAULA, A; SILVA, A.F.; MARCO-JÚNIOR, P.; SANTOS, F.A.M. & SOUZA, A.L. 2004. Sucessão ecológica da vegetação arbórea em uma Floresta Estacional Semidecidual, Viçosa, MG, Brasil. Acta Botanica Brasilica 18(3): 401-699. [TreeCo refID: 224]

PAULA, J. E.; IMAÑA-ENCINAS, J. & SUGIMOTO, N. 1998. Levantamento quantitativo em três hectares de vegetação de cerrado. Pesquisa Agropecuária Brasileira 33(5): 613-620. [TreeCo refID: 2410]

PAULA, J.E. & ALVES, J.L.H. 2010. 922 Madeiras nativas do Brasil: anatomia, dendrologia, dendrometria, produção e uso. Ed. Cinco Continentes, Porto Alegre. 461p. [TreeCo refID: 10078]

PAULA, J.E., IMAÑA-ENCIMAS, J.; PEREIRA, B.A.S. 1993. Inventário de um hectare de Mata Ripária. Pesquisa Agropecuária Brasileira 28(2): 143-152. [TreeCo refID: 2411]

PAULA, J.E.; IMAÑA-ENCINAS, J. & PEREIRA, B.A.S. 1996. Parâmetros volumétricos e da biomassa da mata ripária do Córrego dos Macacos. CERNE 2(2): 91-105. [TreeCo refID: 1882]

PEDREIRA, G. & SOUSA, H.C. 2011. Comunidade arbórea de uma mancha florestal permanentemente alagada e de sua vegetação adjacente em Ouro Preto-MG, Brasil. Ciência Floresta 21(4): 663-675. [TreeCo refID: 103]

PEIXOTO, A.L. 1987. Revisão Taxonomica do Genero Mollinedia Ruiz et Pavon (Monimiaceae, Monimioideae). Universidade Estadual de Campinas, Campinas. 401p. [TreeCo refID: 10079]

PENNINGTON, T. 1990. Sapotaceae. Flora Neotropica 52: 1-770. [TreeCo refID: 10136]

PENNINGTON, T., STYLES, B. & TAYLOR, D.A.H. 1981. Meliaceae, with Accounts of Swietenioideae and Chemotaxonomy. Flora Neotropica 28: 1-470. [TreeCo refID: 10133]

PENNINGTON, T.D. 1990. Sapotaceae. Flora Neotropica Monograph 52: 1-770. [TreeCo refID: 10080]

PENNINGTON, T.D. 1997. The genus Inga: botany. The Royal Botanical Garden, Kew. 844p. [TreeCo refID: 10081]

PENNINGTON, T.R. 2003. Monograph of Andira (Leguminosae-Papilionoideae). Systematic Botany Monographs 64: 1-143. [TreeCo refID: 10123]

PERDIZ, R.O.; FERRUCCI, M.S. & AMORIM, A.M.A. 2014. Sapindaceae em remanescentes de florestas montanas no sul da Bahia, Brasil. Rodriguésia 65(4): 987-1002. [TreeCo refID: 10162]

PEREIRA, M.S. 2007. O genêro Coussarea Aubl. (Rubiaceae, Rubioideae, Coussareae) na Mata Atlântica. Tese (Doutorado). Universidade Federal de Pernambuco, Recife. 136p. [TreeCo refID: 10175]

PEREIRA, Z.V. & KINOSHITA, L.S. 2013. Rubiaceae Juss. of Parque Estadual das Várzeas do Rio Ivinhema, Mato Grosso do Sul State, Brazil. Hoehnea 40(2): 205-251. [TreeCo refID: 10082]

PILLAR, V.D. & SOSINSKI, E. 2003. An improved method for searching plant functional types by numerical analysis. Journal of Vegetation Science 14: 323-332. (TRY dataset 77: FAPESP Brazil Rainforest Database). [TreeCo refID: 10425]

PIÑA- RODRIGUES, F.C.M.; FREIRE, J.M.; LELES, P.S.S.; BREIER, T.B. (Org.). Parâmetros técnicos para produção de sementes florestais. Editora da UFRRJ, Seropédica. 188p. [TreeCo refID: 10083]

PINHEIRO, K.; ALVES, M. 2007 Espécies arbóreas de uma área de Caatinga no sertão de Pernambuco, Brasil: dados preliminares. Revista Brasileira de Biociências 5(sup): 426-428. [TreeCo refID: 3506]

PIRANI, J.R. 1987. Flora da Serra do Cipó: Burseraceae. Boletim de Botânica 9: 211-218. [TreeCo refID: 10084]

PIRANI, J.R. 1998. A revision of Helietta and Balfourodendron (Rutaceae-Pteleinae). Brittonia 50(3): 348-380. [TreeCo refID: 10177]

POSSETTE, R.F.DA.S. & RODRIGUES, W.A. 2010. O gênero Inga Mill. (Leguminosae - Mimosoideae) no estado do Paraná, Brasil. Acta Bot. Bras. 24(2): 354-368. [TreeCo refID: 10180]

PRADO-JÚNIOR, J.A.; LOPES, S.F.; VALE, V.S.; OLIVEIRA, A.P.; GUSSON, A.E.; DIAS-NETO, O.C. & SCHIAVINI, I. 2011. Estrutura e caracterização sucessional da comunidade arbórea de um remanescente de floresta estacional semidecidual, Uberlândia, MG. Caminhos de Geografia 12(39): 81-93. [TreeCo refID: 235]

PRADO-JÚNIOR, J.A.P.; VALE, V.S.; OLIVEIRA, A.P.; GUSSON, A.E.; DIAS-NETO, O. C.; LOPES, S.F. & SCHIAVINI, I. 2010. Estrutura da comunidade arbórea em um fragmento de Floresta Estacional Semidecidual localizada na Reserva Legal da Fazenda Irara, Uberlândia, MG. Bioscience Journal 26(4): 638-647. [TreeCo refID: 100]

PRADO JUNIOR, J.A.; FARIA, S.; SCHIAVINI, I.; VALE, V.; OLIVEIRA, A. P.; GUSSON, A. E.; DIAS, N.; OLAVO, C. & STEIN, M. 2012. Fitossociologia, caracterização sucessional e síndromes de dispersão da comunidade arbórea de remanescente urbano de Floresta Estacional Semidecidual em Monte Carmelo, Minas Gerais. Rodriguésia 63(3): 489-499. [TreeCo refID: 1307]

PRANCE, G. & MORI, S. 1979. Lecythidaceae: Part I: The Actinomorphic-Flowered New World Lecythidaceae (Asteranthos, Gustavia, Grias, Allantoma, & Cariniana). Flora Neotropica 21(1): 1-270. [TreeCo refID: 10140]

PRANCE, G.T.; PLANA, V.; EDWARDS, K.S. & PENNINGTON, R.T. 2007. Proteaceae. Flora Neotropica 100: 1-218. [TreeCo refID: 10434]

PROENÇA, C. 1986. Revisão de Siphoneugena (Myrtaceae, Myrteae). Dissertação (Mestrado). Universidade Federal do Rio de Janeiro, Rio de Janeiro. 163p. [TreeCo refID: 10159]

QUIRINO, W.F.; VALE, A.T.; ANDRADE, A.P.A.; ABREU, V.L.S. & AZEVEDO, A.D.S. 2005. Poder calorífico da madeira e de materiais ligno-celulósicos.Revista da Madeira 89: 100-106. [TreeCo refID: 10085]

Rede speciesLink. Data retrieved for record descriptions or images from the speciesLink netwrok. http://www.splink.org.br. [TreeCo refID: 10428]

REIS, A. 1993. Manejo e conservação das florestas catarinenses (Trabalho apresentado para o Concurso de Professor Titular de Botânica Aplicada). Universidade Federal de Santa Catarina, Florianópolis. [TreeCo refID: 10086]

REITZ, P.R. 1965. Plano de coleção. In: REITZ, R. (ed). Flora Ilustrada Catarinense. Herbário Barbosa Rodrigues, Itajaí. 71p. [TreeCo refID: 10087]

REITZ, R. (ED.). 1965 - 1985. Flora Ilustrada Catarinense. Herbário Barbosa Rodrigues, Itajaí. [TreeCo refID: 10176]

REITZ, R.; KLEIN, R.M. & REIS, A. 1979. Madeiras do Brasil - Santa Catarina. REITZ, R. (Ed.). Editora Lunardelli, Florianópolis. 320 p.|REITZ,R.; KLEIN, R.M. & REIS, A. 1983. Projeto Madeira do Rio Grande do Sul. Sellowia 34-35. 525p. [TreeCo refID: 10116]

RENNER, S. & HAUSNER, G. 2005. Siparunaceae. Flora Neotropica 95: 1-247. [TreeCo refID: 10433]

RIBEIRO, B.R. 2014. Onde estão os recrutas? A matriz de pasto pode acelerar alterações na assembléia de árvores grandes em áreas fragmentadas. Monografia (Graduação). Universidade Federal de Alfenas, Alfenas. 39p. [TreeCo refID: 3851]

RIBEIRO, S.C.; FEHRMANN, L.; SOARES, C.P.B.; JACOVINE, L.A.G.; KLEINN, C. & GASPAR, R.O. 2011. Above-and belowground biomass in a Brazilian Cerrado. Forest Ecology and Management 262(3): 491-499. [TreeCo refID: 10088]

ROCHA, D.S.B & AMORIM, A.M.A. 2012. Heterogeneidade altitudinal na Floresta Atlântica setentrional: um estudo de caso no sul da Bahia, Brasil. Acta Botanica Brasilica 26(2): 309-327. [TreeCo refID: 10089]

RODRIGUES, A.V.; BONES, F.L.V.; SCHNEIDERS, A.; OLIVEIRA, L.Z.; VIBRANS, A.C. & GASPER, A.L. 2018. Plant trait dataset for tree-like growth forms species of the subtropical Atlantic Rain Forest in Brazil. Data 3(2): 16. [TreeCo refID: 10090]

RODRIGUES, I.A. 1982. Contribuição à sistemática das espécies do gênero Inga P. Mill. (Leg. Mim.) ocorrentes no Estado do Rio de Janeiro. Dissertação Mestrado, UFRJ, Rio de Janeiro. [TreeCo refID: 10092]

RODRIGUES, I.M.C. & GARCIA, F.C.P. 2007. Papilionoideae (Leguminosae) arbóreas e lianas na estacão de pesquisa, treinamento e educação ambiental (EPTEA), Mata do Paraíso, Viçosa, Zona da Mata Mineira. Revista Árvore 31(3):521-532. [TreeCo refID: 10091]

RODRIGUES, L.A.; CARVALHO, D.A.; OLIVEIRA-FILHO, A.T.; BOTREL, R.T. & SILVA, E.A. 2003. Florística e estrutura da comunidade arbórea de um fragmento florestal em Luminárias, MG. Acta Botanica Brasilica 17(1): 71-87. [TreeCo refID: 75]

RODRIGUES, R.R.; GANDOLFI, S. & SOUZA, V.C. 2006. Diversidade, dinâmica e conservação em florestas do estado de São Paulo: 40,96ha de parcelas permanentes. Universidade de São Paulo, Piracicaba, Brazil, 68p. [TreeCo refID: 936]

RODRIGUES, V.H.P.; LOPES, S.F.; ARAÚJO, G.M. & SCHIAVINI, I. 2010. Composição, estrutura e aspectos ecológicos da floresta ciliar do rio Araguari no Triângulo Mineiro. Hoehnea 37(1): 87-105. [TreeCo refID: 76]

ROHWER, J. 1993. Lauraceae: Nectandra. Flora Neotropica 60: 1-332. [TreeCo refID: 10142]

ROSEIRA, D.S. 1990. Composição florística e estrutura fitossociológica do bosque com Araucaria angustifolia (Bert.) Kuntze no Parque Estadual João Paulo II, Curitiba, Paraná. Dissertação (Mestrado). Universidade Federal do Paraná, Curitiba. 111p. [TreeCo refID: 411]

ROYAL BOTANIC GARDENS KEW. 2017. Seed Information Database (SID). Version 7.1. <http://data.kew.org/sid/> Accessed on: March 2017. [TreeCo refID: 10094]

SÁ, C.F.C. & ARAUJO, D.S.D. 2009. Estrutura e florística de uma floresta de Restinga em Ipitangas, Saquarema, Rio de Janeiro, Brasil. Rodriguésia 60: 147-170. [TreeCo refID: 2848]

SALES, H.R.; SOUZA, S.C.A.; LUZ, G.R.; MORAIS-COSTA, F.; AMARAL, V.B.; SANTOS, R.M.; VELOSO, M.D.M. & NUNES, Y.R.F. 2009. Flora arbórea de uma Floresta Estacional Decidual na APA Estadual do Rio Pandeiros, Januária, MG. Biota 2(3): 31-41. [TreeCo refID: 244]

SALIS, S.M.; LEHN, C.R.; PADILHA, D.R.C. & MATTOS, P.P. 2012. Changes in the structure due to strong winds in forest areas in the Pantanal, Brazil. CERNE 18(3): 387-395. [TreeCo refID: 2350]

SALOMÃO, N.A.; DAVIDE, A.C.; FIRETTI, F.; SOUSA E SILVA, F.C.; CALDAS, L.S.; WETZEL, M.M.V.S.; TORRES, R.A.A. & GONZÁLES, S. 2003. Germinação de sementes e produção de mudas de plantas do Cerrado. Rede de Sementes do Cerrado, Brasília. 93p. [TreeCo refID: 10095]

SALYWON, A.M. & LANDRUM, L.R. 2007. Curitiba (Myrtaceae): A New Genus from the Planalto of Southern Brazil. Brittonia 59(4): 301-307. [TreeCo refID: 10096]

SAMPAIO, A.B.; WALTER, B.M.T. & FELFILI, J.M. 2000. Diversidade e distribuição de espécies arbóreas em duas matas de galeria do riacho Fundo, Distrito Federal. Acta Botanica Brasilisa 14(2): 197-214. [TreeCo refID: 1268]

SAMPAIO, D. 2009. Revisão taxonômica das espécies neotropicais extra-amazônicas de Sloanea L. (Elaeocarpaceae) na América do Sul. Tese (Doutorado). Universidade Estadual de Campinas, Campinas. 168p. [TreeCo refID: 10165]

SANTANA, G.C. 2010. Estrutura de uma floresta ombrófila densa montana com monodominância de dossel por Eremanthus erythropappus (DC.) Macleish (candeia) na serra da Mantiqueira, em Itamonte, Minas Gerais. Dissertação (Mestrado). Universidade Federal de Lavras, Lavras. 58p. [TreeCo refID: 245]

SANTIN, D.A. 1989. Revisão taxonômica do gênero Astronium Jacq. e revalidação do gênero Myracrodruon Fr. Allem. (Anacardiaceae). Dissertação de mestrado, Universidade Estadual de Campinas, Campinas. 187p. [TreeCo refID: 10160]

SANTOS, I.S. & PEIXOTO, A.L. 2001. Taxonomia do gênero Macropeplus Perkins (Monimiaceae, Monimioideae). Rodriguesia 52(81): 65-105. [TreeCo refID: 10097]

SANTOS, K. 2003. Caracterização florística e estrutural de onze fragmentos de mata estacional semidecidual da área de proteção ambiental do município de Campinas – SP. Tese (Doutorado). Universidade de Campinas, Campinas. 225p. [TreeCo refID: 2809]

SARTORI, Â.L.B.; LEWIS, G.P.; MANSANO, V.F. & TOZZI, A.M.G.A. 2015. A revision of the genus Myroxylon (Leguminosae: Papilionoideae). Kew Bulletin 70(4): 48. [TreeCo refID: 10098]

SCHORN, L.A. 2005. Estrutura e dinâmica de estágios sucessionais de uma floresta ombrófila densa em Blumenau, Santa Catarina. Tese (Doutorado). Universidade Federal do Paraná, Curitiba. 192p. [TreeCo refID: 855]

SCHORN, L.A.; GASPER, A.L. de; MEYER, L.; VIBRANS, A.C. 2012. Síntese da estrutura dos remanescentes florestais em Santa Catarina. In: Vibrans, A.C., Sevegnani, L.; Gasper A.L.; Lingner, D.V. (Org.). 2012. Inventário Florístico Florestal de Santa Catarina, Vol. 1, Diversidade e Conservação dos remanescentes florestais. Edifurb, Blumenau, 1 ed: 125-140 (Classificação consensuada utilizada pela FURB a partir de 2011 para o IFSC) [TreeCo refID: 10125]

SECCO, R.S. 2004. Alchorneae (Euphorbiaceae) (Alchornea, Aparisthmium e Conceveiba). Flora Neotropica 93: 1-194. [TreeCo refID: 10432]

SECRETARIA DO MEIO AMBIENTE DO ESTADO DE SÃO PAULO. 1991. Anais do 2° Simpósio Brasileiro sobre Tecnologia de Sementes Florestais. Atibaia, São Paulo, 16 a 19 de outubro de 1989. Instituto Florestal, São Paulo. 319p. [TreeCo refID: 10100]

SENA, C.M. & GARIGLIO, M.A. 2008. Sementes Florestais: Colheita, Beneficiamento e Armazenamento. MMA, Secretaria de Biodiversidade e Florestas, Departamento de Florestas, Programa Nacional de Florestas, Natal. 28p. [TreeCo refID: 10101]

SENA, L.H.M. 2014. Conservação de sementes e produção de mudas de pitombeira (Talisia esculenta (A. St. Hil.) Radlk.). Dissertação (Mestrado). Universidade Federal Rural de Pernambuco, Recife. 122p. [TreeCo refID: 10414]

SENNA, L.M. 1984. Maprounea Aubl. (Euphorbiaceae). Considerações taxonômicas e anatômicas das espécies sul-americanas. Rodriguesia 36(61): 51-78. [TreeCo refID: 10164]

SEVEGNANI, L. (Unpublished data) Information on species traits compiled by Lucia Sevegnani. FURB, Blumenau. [TreeCo refID: 10422]

SEVILHA, A.C.; PAULA, A; LOPES, W.P. & SILVA, A.F. 2001. Fitosociologia de estrato arbóreo de um trecho de Floresta Estacional no Jardim Botânico da Universidade Federal de Viçosa (face sudoeste), Viçosa, Minas Gerais. Revista Árvore 25(4): 431-443. [TreeCo refID: 256]

SILVA-JÚNIOR, J.F. 2004. Estudo fitossociológico em um remanescente de floresta atlântica visando dinâmica de espécies florestais arbóreas no município do Cabo de Santo Agostinho, PE. Dissertação (Mestrado). Universidade Federal Rural de Pernambuco, Recife. 74p. [TreeCo refID: 1570]

SILVA JÚNIOR, M.C.DA. & PEREIRA, B.A.S. 2009. 100 Arvores do Cerrado-Matas de Galeria. Editora Rede de Sementes do Cerrado, Brasilia. 288p. [TreeCo refID: 10061]

SILVA JÚNIOR, M.C.DA. 2005. 100 Árvores do cerrado: guia de campo. Editora Rede de Sementes do Cerrado, Brasilia. 278p. [TreeCo refID: 10001]

SILVA, A.C.; HIGUCHI, P.; AGUIAR, M.D.; NEGRINI, M.; FERT NETO, J. & HESS, A.F. 2012. Relações florísticas e fitossociologia de uma floresta ombrófila mista montana secundária em Lages, Santa Catarina. Ciência Florestal 22(1): 193-206. [TreeCo refID: 828]

SILVA, A.C.D.; VAN DEN BERG, E.; HIGUCHI, P.; OLIVEIRA-FILHO, A.T.; MARQUES, J.J.G.D.S.; APPOLINÁRIO, V.; PIFANO, D.S.; OGOSUKU, L.M. & NUNENS, M. 2009. Tree community floristic and structure of alluvial forest fragments in São Sebastião da Bela Vista, Minas Gerais, Brazil. Brazilian Journal of Botany 32(2): 283-297. [TreeCo refID: 60]

SILVA, C.V. & REIS, M.S. 2009. Produção de pinhão na região de Caçador, SC: aspectos da obtenção e sua importância para comunidades locais. Ciência Florestal 19(4): 363-374. [TreeCo refID: 10406]

SILVA, M.J. & TOZZI, A.M.G.A. 2012. Revisão taxonômica de Lonchocarpus s. str. (Leguminosae, Papilionoideae) do Brasil. Acta Botanica Brasilica 26(2): 357-377. [TreeCo refID: 10104]

SILVA, M.S.; SANTOS, F.A.R.; SILVA, C.R.A. & SILVA, L.B. 2012. Características das fibras e densidade básica da madeira de quatro espécies de Mata Atlântica (Serra da Jibóia, Elísio Medrado, Bahia, Brasil): qualificação para uso e preservação. I Simpósio sobre a Biodiversidade da Mata Atlântica: 129-133. [TreeCo refID: 10062]

SILVA, R.K.S. 2009. Fitossociologia do componente arbóreo em áreas ciliares e de nascentes de um fragmento de floresta ombrófila densa de terras baixas, em Sirinhaém, Pernambuco. Dissertação (Mestrado). Universidade Federal Rural de Pernambuco, Recife. 80p. [TreeCo refID: 2740]

SILVEIRA, N.M.; ALVES, J.D.; DOUSSEAU, S. & ALVARENGA, A.A. 2013. Technology seed Sebastiania membranifolia Mull Arg (Euphorbiaceae). Cerne 19(4): 669-75. [TreeCo refID: 10410]

SILVEIRA, P. 2008. Métodos indiretos de estimativa do conteúdo de biomassa e do estoque de carbono em um fragmento de floresta ombrófila densa. Tese (Doutorado). Universidade Federal do Paraná, Curitiba. 129p. [TreeCo refID: 10105]

SLEUMER, H.O. 1980. Flacourtiaceae. Flora Neotropica 22: 1-499. [TreeCo refID: 10132]

SLEUMER, H.O. 1984. Olacaceae. Flora Neotropica 38: 1-158. [TreeCo refID: 10438]

SLUSARSKI, S.R. & SOUZA, M.C. 2012. Analysis of floristic similarity between forest remnants from the upper Paraná river floodplain, Brazi. Acta Scientiarum, Biological Sciences 34(3): 343-352. [TreeCo refID: 1159]

SOBRAL, M. 2011. Eugenia (Myrtaceae) no Parana. Eduel, Londrina. 236 p. [TreeCo refID: 10166]

SOSINSKI, E.; JOLY, C.A. & PILLAR, V.D. (Unpublished data). TRY dataset 77: FAPESP Brazil Rainforest Database. [TreeCo refID: 10424]

SOUSA-JÚNIOR, P.R.C. 2006. Estrutura da comunidade arbórea e da regeneração natural em um fragmento de floresta urbana, Recife - PE. Dissertação (Mestrado). UFRPE, Recife. 91p. [TreeCo refID: 2636]

SOUZA, A.L.; BOINA, A.; SOARES, C.P.B.; VITAL, B.R.; GASPAR, R.O. & LANA, J.M. 2012. Estrutura fitossociológica, estoques de volume, biomassa, carbono e dióxido de carbono em floresta estacional semidecidual. Revista Árvore 36(1): 169-179. [TreeCo refID: 97]

SOUZA, I.M.; FUNCH, L.S. & QUEIROZ, L.P 2016. Flora da Bahia: Leguminosae – Hymenaea (Caesalpinioideae: Detarieae). Sitientibus, série Ciências Biológicas 16: http://dx.doi.org/10.13102/scb1092 [TreeCo refID: 10179]

SOUZA, J.S.; ESPÍRITO-SANTO, F.D.B.; FONTES, M.A.L.; OLIVEIRA-FILHO, A.T. & BOTEZELLI, L. 2003. Análise das variações florísticas e estruturais da comunidade arbórea de um fragmento de floresta semidecídua às margens do rio Capivari, Lavras-MG. Revista Árvore 27(2): 185-206. [TreeCo refID: 82]

SOUZA, M.C. 2009. Estudos taxônomicos em Myrtaceae no Brasil: Revisão de Neomitranthes Kausel ex D.Legrand e contribuição ao conhecimento da diversidade e conservação de Plinia L. (Myrtaceae Juss.) no Dominío Atlântico. Tese (Doutorado). Jardim Botânico do Rio de Janeiro, Rio de Janeiro. [TreeCo refID: 10107]

SPINA, A.P. 2004. Estudos taxonomico, micro-morfologico e filogenetico do genero Himatanthus Willd. ex Schult. (Apocynaceae: Rauvolfioideae - Plumerieae). Tese (Doutorado). Universidade Estadual de Campinas, Campinas. 191p. [TreeCo refID: 10178]

SÜHS, R.B.; PUTZKE, J. & BUDKE, J.C. 2010. Relações florístico-geográficas na estrutura de uma floresta na região central do Rio Grande do Sul, Brasil. Floresta 40(3): 635-646. [TreeCo refID: 741]

TABANEZ, A.A.J.; VIANA, V.M. & DIAS, A.S. 1997. Conseqüências da fragmentação e do efeito de borda sobre a estrutura, diversidade e sustentabilidade de um fragmento de floresta de Planalto de Piracicaba, SP. Revista Brasileira de Biologia 57(1): 47-60. [TreeCo refID: 1077]

TAMASHIRO, J.Y. 1989. Estudos taxonomicos e morfologicos do genero Piptadenia sensu Dentham no sudoeste do Brasil: avaliação das modificações taxonomicas recentemente propostas. Dissertação (Mestrado). Universidade Estadual de Campinas, Campinas. 99p. [TreeCo refID: 10188]

TAVARES, R.P. 2010. Morfoanatomia foliar de espécies de Brunfelsia L. do Sul do Brasil. Dissertação (Mestrado). Universidade Federal de Santa Catarina, Florianópolis. 76p. [TreeCo refID: 10191]

THOMAS, W.W.; CARVALHO, A.M.V.; AMORIM, A.M.; HANKS, J.G. & SANTOS, T.S. 2008. Diversity of Woody Plants in the Atlantic Coastal Forest of Southern Bahia, Brazil. In: W.W. THOMAS (ed.). The Atlantic Coastal Forest of Northeastern Brazil. Memoirs of the New York Botanical Garden 100: 21-66. [TreeCo refID: 10109]

TOMASETTO, F. 2003. Composição florística e estrutura do componente arbóreo de um trecho de floresta estacional semidecidual na Estação Ecológica de Paulo de Faria – SP. Dissertação (Mestrado). Universidade Estadual Paulista, Rio Claro. 133 p. [TreeCo refID: 1344]

TONIATO, M.T.Z.; OLIVEIRA-FILHO, A.T. 2004. Variations in tree community composition and structure in a fragment of tropical semideciduous forest in southeastern Brazil related to different human disturbance histories. Forest Ecology and Management 198(1): 319-339. [TreeCo refID: 889]

TOZZI, A.M.G.A. 1989. Estudos taxônomicos dos gêneros Lonchocarpus Kunth e Deguelia Aubl. no Brasil. Tese (Doutorado). Universidade Estadual de Campinas, Campinas. 341p. [TreeCo refID: 10110]

TOZZI, A.M.G.A.; MELHEM, T.S.; FORERO, E.; FORTUNA-PEREZ, A.P.; WANDERLEY, M.G.L.; MARTINS, S.E.; ROMANINI, R.P.; PIRANI, J. R.; MELO, M.M.R.F.DE.; KIRIZAWA, M.; YANO, O. & CORDEIRO, I (eds.). 2016. Flora Fanerogâmica do Estado de São Paulo, Vol. VIII, Leguminosae. Instituto de Botânica, São Paulo. 441p. [TreeCo refID: 10036]

VALE, A.T. (Unpublished data). Measurements of wood specific gravity made by Ailton T. Vale for Cerrado species. Universidade de Brasília, Brasília. [TreeCo refID: 10419]

VALE, A.T. 2000. Caracterização da biomassa lenhosa de um cerrado sensu stricto da região de Brasília para uso energético. Tese (Doutorado). Universidade Estadual de São Paulo "Júlio de Mesquita Filho", Botucatu. 111p. | VALE, A.T.; BRASIL, M.A.M.; LEÃO, A.L. 2002. Quantificação e caracterização energética da madeira e casca de espécies do cerrado. Ciência Florestal, 12(1):71-80. | VALE, A.T. & FELFILI, J.M. 2005. Dry biomass distribution in a cerrado sensu stricto site in Brazil central. Revista Árvore, 29(5): 661-669. [TreeCo refID: 10119]

VALE, A.T.; DIAS, Í.S. & SANTANA, M.A.E. 2010. Relações entre propriedades químicas, físicas e energéticas da madeira de cinco espécies de cerrado. Ciência Florestal 20(1): 137-145. [TreeCo refID: 10111]

VAZ, A.M.S.F. & TOZZI, A.M.G.A 2003. Bauhinia ser. Cansenia (Leguminosae: Caesalpinioideae) no Brasil. Rodriguésia 54(83): 55-143. [TreeCo refID: 10122]

VEIGA, L.G. 2010. Estoque de madeira morta ao longo de um gradiente altitudinal de Mata Atlântica no nordeste do estado de São Paulo. Dissertação (Mestrado). Universidade Estadual de Campinas, Campinas. 71p. [TreeCo refID: 10112]

VENZKE, T.S.L. 2012. Florística, estrutura e síndrome de dispersão de sementes em estágios sucessionais de mata ciliar no município de Arroio do Padre, RS, Brasil. Dissertação (mestrado). Universidade Federal de Viçosa, Viçosa, 82p. [TreeCo refID: 798]

VIANI, R.A.G.; COSTA, J.C.; ROZZA, A.F.; BUFO, L.B.V.; FERREIRA, M.A.P. & OLIVEIRA, A.C.P. 2011. Caracterização florística e estrutural de remanescentes florestais de Quedas do Iguaçu, Sudoeste do Paraná. Biota Neotropica 11(1): 115-128. [TreeCo refID: 360]

VIGNOLI-SILVA, M. 2009. O gênero Cestrum L. (Solanaceae) no Brasil extra-amazônico. Tese (Doutorado). Universidade Federal do Rio Grande do Sul, Porto Alegre. 317p. [TreeCo refID: 10173]

VILELA, E.A.; OLIVEIRA-FILHO, A.T.; CARVALHO, D.A. & CURI. N. 1998. Estudos florísticos e fitossociológicos em remanescentes de florestas ripárias do Baixo Rio Paranaíba e Alto Rio São Francisco. Boletim técnico 01000-GE/PA-1, Companhia Energética de Minas Gerais (CEMIG) , Belo Horizonte. 23p. [TreeCo refID: 274]

WANDERLEY, M.G.L.; SHEPHERD, G.J. & GIULIETTI, A.M. (coords.). 2002. Flora Fanerogâmica do Estado de São Paulo. Vol. 2. Ed. Hucitec, São Paulo. 386p. [TreeCo refID: 10144]

WANDERLEY, M.G.L.; SHEPHERD, G.J.; GIULIETTI, A.M. & MELHEM, T.S. (coords.). 2003. Flora Fanerogâmica do Estado de São Paulo. Vol. 3. Ed. Hucitec, São Paulo. 386p. [TreeCo refID: 10145]

WANDERLEY, M.G.L.; SHEPHERD, G.J.; MELHEM, T.S. & GIULIETTI, A.M. (coords.) 2007. Flora Fanerogâmica do Estado de São Paulo. Vol. 5. Ed. Hucitec, São Paulo. 523p. [TreeCo refID: 10147]

WANDERLEY, M.G.L.; SHEPHERD, G.J.; MELHEM, T.S. & GIULIETTI, A.M. (coords.). 2005. Flora Fanerogâmica do Estado de São Paulo. Vol. 4. Ed. Hucitec, São Paulo. 437p. [TreeCo refID: 10146]

WANDERLEY, M.G.L.; SHEPHERD, G.J.; MELHEM, T.S.; GIULIETTI, A.M. & MARTINS, S.E. (coords.) 2009. Flora Fanerogâmica do Estado de São Paulo. Vol. 6. Ed. Hucitec, São Paulo. 330p. [TreeCo refID: 10148]

WANDERLEY, M.G.L.; SHEPHERD, G.J.; MELHEM, T.S.; GIULIETTI, A.M. & MARTINS, S.E. (coords.) 2012. Flora Fanerogâmica do Estado de São Paulo. Vol. 7. Ed. Hucitec, São Paulo. 393p. [TreeCo refID: 10149]

WEBSTER, G.L. 1982. Systematic status of the genus Kleinodendron (Euphorbiaceae). Taxon 31(3): 535-539. [TreeCo refID: 10431]

WIESBAUER, M.B.; GIEHL, E.L.H. & JARENKOW, J.A. 2008. Padrões morfológicos de diásporos de árvores e arvoretas zoocóricas no Parque Estadual de Itapuã, RS, Brasil. Acta Bot Brasilica 22(2):425-435. [TreeCo refID: 10113]

WITTMANN F.; SCHÖNGART J.; PAROLIN P.; WORBES M.; PIEDADE MTF. & JUNK W.J. 2006b. Wood specific gravity of trees in Amazonian white-water forests in relation to flooding. IAWA J, 27:255–266. apud WITTMANN, F.; ZORZI, B.T.; TIZIANEL, F.A.T.; URQUIZA, M.V.S.; FARIA, R.R.; SOUZA, N.M.; MÓDENA, É.S.; GAMARRA, R.M. &

ROSA, A.L.M. 2008. Tree species composition, structure, and aboveground wood biomass of a riparian forest of the Lower Miranda River, southern Pantanal, Brazil. Folia Geobotânica 43(4): 397-411. [TreeCo refID: 10168]

WITTMANN, F.; SCHÖNGART, J.; DE BRITO, J.M.; WITTMANN, A.DEO.; PIEDADE, M.T.F.; PAROLIN, P.; JUNK, W.J. & GUILLAUMET, J.-L. 2010. Manual of trees in Central Amazonian várzea floodplains: taxonomy, ecology, and use. Editora INPA, Manaus. 286p. apud WITTMANN, F.; ZORZI, B.T.; TIZIANEL, F.A.T.; URQUIZA, M.V.S.; FARIA, R.R.; SOUZA, N.M.; MÓDENA, É.S.; GAMARRA, R.M. & ROSA, A.L.M. (2008). Tree species composition, structure, and aboveground wood biomass of a riparian forest of the Lower Miranda River, southern Pantanal, Brazil. Folia Geobotânica 43(4), 397-411. [TreeCo refID: 10169]

WITTMANN, F.; ZORZI, B.T.; TIZIANEL, F.A.T.; URQUIZA, M.V.S.; FARIA, R.R.; SOUSA, N.M.; MÓDENA, É.D.S.; GAMARRA, R.M. & ROSA, A.L.M. 2008. Tree species composition, structure, and aboveground wood biomass of a riparian forest of the lower Miranda River, Southern Pantanal, Brazil. Folia Geobotanica 43(4): 397-411. [TreeCo refID: 10118]

YUNCKER, T.G. 1972. The Piperaceae of Brazil I: Piper -Group I, II, III, IV. Hoehnea 2: 19-366. [TreeCo refID: 10342]

YUNCKER, T.G. 1973. The Piperaceae of Brazil II: Piper: Group V; Ottonia; Pothomorphe; Sarcorhachis. Hoehnea 3: 29-284. [TreeCo refID: 10343]

ZAMA, M.Y.; BOVOLENTA, Y.R.; CARVALHO, E.D.S.; RODRIGUES, D.R.; ARAUJO, C.G.D.; SORACE, M.A.D.F. & LUZ, D.G. 2012. Floristic composition and diaspore dispersal syndromes of shrubs and tree species in Parque Estadual Mata São Francisco, Paraná State, Brazil. Hoehnea 39(3): 369-378. [TreeCo refID: 10117]

ZAPPI, D. 2003. Revision of Rudgea (Rubiaceae) in Southeastern and Southern Brazil. Kew Bulletin 58(3): 513-596. [TreeCo refID: 10115]

ZECHINI, A.A.; SCHUSSLER, G.; SILVA, J.Z.; MATTOS, A.G.; PERONI, N.; MANTOVANI, A. & REIS, M.S. 2012. Produção, comercialização e identificação de variedades de pinhão no entorno da Floresta Nacional de Três Barras–SC. Biodiversidade Brasileira 2(2): 74-82. [TreeCo refID: 10404]

ZICKEL, C.S. 1989. Revisão taxonômica do gênero Lamanonia Vell. (Cunoniaceae). Dissertação (Mestrado). Universidade Estadual de Campinas, Campinas. 116p. [TreeCo refID: 10182]
