# Supplementary material for: The erosion of biodiversity and biomass in the Atlantic Forest biodiversity hotspot
Source: Nat Commun. 2020 Dec 11;11:6347. doi: 10.1038/s41467-020-20217-w (PMC7733445; doi:10.1038/s41467-020-20217-w)
Supplement: Supplementary file 6 — Reporting Summary [file 41467_2020_20217_MOESM6_ESM.pdf]

nature research

Corresponding author(s): Renato A. F. de Lima

Last updated by author(s): Oct 29, 2020

Reporting Summary

Nature Research wishes to improve the reproducibility of the work that we publish. This form provides structure for consistency and transparency in reporting. For further information on Nature Research policies, see Authors & Referees and the Editorial Policy Checklist .

Statistics

For all statistical analyses, confirm that the following items are present in the figure legend, table legend, main text, or Methods section.

n/a	Confirmed	
☐	☒	The exact sample size (*n*) for each experimental group/condition, given as a discrete number and unit of measurement
☐	☒	A statement on whether measurements were taken from distinct samples or whether the same sample was measured repeatedly
☐	☒	The statistical test(s) used AND whether they are one- or two-sided<br>*Only common tests should be described solely by name; describe more complex techniques in the Methods section.*
☐	☒	A description of all covariates tested
☐	☒	A description of any assumptions or corrections, such as tests of normality and adjustment for multiple comparisons
☐	☒	A full description of the statistical parameters including central tendency (e.g. means) or other basic estimates (e.g. regression coefficient) AND variation (e.g. standard deviation) or associated estimates of uncertainty (e.g. confidence intervals)
☐	☒	For null hypothesis testing, the test statistic (e.g. *F*, *t*, *r*) with confidence intervals, effect sizes, degrees of freedom and *P* value noted<br>*Give P values as exact values whenever suitable.*
☐	☒	For Bayesian analysis, information on the choice of priors and Markov chain Monte Carlo settings
☒	☐	For hierarchical and complex designs, identification of the appropriate level for tests and full reporting of outcomes
☐	☒	Estimates of effect sizes (e.g. Cohen's *d*, Pearson's *r*), indicating how they were calculated

Our web collection on statistics for biologists contains articles on many of the points above.

Software and code

Policy information about availability of computer code

Data collection	No specific code was used to collect the data used in this study. All data extraction and processing from SIG products were performed in R (version 3.5.1), using the packages ‘raster’ (version 2.5-8), ‘rgdal’ (version 1.3.6), ‘rgeos’ (version 0.4.2), ‘sp’ (version 1.2.7), ‘maptools’ (version 0.9.4), ‘SDMTools’ (version version 1.1-20), ‘fasterize’ (version 1.0) and ‘cleangeo’ (version 0.2.2).
Data analysis	All data analyses were performed in R (version 3.5.1), using the packages ‘ridittools’ (version 0.1), ‘lme4’ (version 1.1.19), ‘piecewiseSEM’ (version 2.0.2), ‘MuMIn’ (version 1.15.6), ‘r2glmm’ (version 0.1), ‘MCMCglmm’ (version 2.26), ‘segmented’ (version 0.5.3), ‘merTools’ (version 0.4.1), and ‘landscapeR’ (version 1.2).

For manuscripts utilizing custom algorithms or software that are central to the research but not yet described in published literature, software must be made available to editors/reviewers. We strongly encourage code deposition in a community repository (e.g. GitHub). See the Nature Research guidelines for submitting code & software for further information.

Data

Policy information about availability of data

All manuscripts must include a data availability statement. This statement should provide the following information, where applicable:

- Accession codes, unique identifiers, or web links for publicly available datasets
- A list of figures that have associated raw data
- A description of any restrictions on data availability

Survey, species abundances and trait data used in this study were extracted from the Neotropical Tree Communities database (TreeCo, version 4.0) and are available upon request at http://labtrop.ib.usp.br/doku.php?id=projetos:treeco:start. The list of surveys extracted from the TreeCo database is provided in Supplementary Data 1, together with the corresponding metadata. The sources of survey and species abundance data are referenced in Supplementary Data 1 and sources of species properties data referenced in the Methods or in the Supplementary Notes. The soil profile database used in this study was accessed at: www.esalq.usp.br/gerd. Other relevant data are available from the corresponding author upon reasonable request. Source data for Figures 1-7 and Tables 2-3 are provided with this paper.

Field-specific reporting

Please select the one below that is the best fit for your research. If you are not sure, read the appropriate sections before making your selection.

☐ Life sciences ☐ Behavioural & social sciences ☒ Ecological, evolutionary & environmental sciences

For a reference copy of the document with all sections, see nature.com/documents/nr-reporting-summary-flat.pdf

Ecological, evolutionary & environmental sciences study design

All studies must disclose on these points even when the disclosure is negative.

Study description	Ground-data on forest biomass, species diversity and trait composition were compiled from 1,819 published surveys (total of 1.45 million trees and 1,238 hectares) and used to quantify human-induced impacts in Atlantic Forest remnants. Forest descriptions and species abundances were extracted from surveys and associated with environmental, human-related, methodological and bio-geographical co-variables. We also obtained from the literature information on different species attributes. Linear mixed-effects regression models were used to quantify site-specific human-impacts, based on differences between observed data and predictions from a human-free scenario. The human impact for each response variable was related to each other. The type and size of protected areas are used to explain possible variations in human impacts. Finally, the impact on forest biomass was used to project and value carbon losses across the entire Atlantic Forest.
Research sample	All data comes from the TreeCo database (version 4.0) that stores published information on the structure, diversity and composition of Neotropical tree communities and on the attributes of their species. The database is composed by different tables, namely: the survey table (survey methods, results and associated metadata), the species abundance table (species names, abundance and vouchers per survey), the environmental table (climate, topography, soil, landscape and patch metrics) and the species trait table (species names and their traits). All information is associated with a reference or source from which surveys or species/trait records were compiled. Species names in TreeCo follow the Brazilian Flora project.
Sampling strategy	All surveys of natural Atlantic Forests (diameter at breast height (dbh) ≥3, ≥5 and ≥10 cm) available in the TreeCo database were included in the analysis, with exception to dry deciduous and early secondary forests. Since the data come from published studies, no statistical methods were used to pre-determine the sample sizes. Therefore, all sample sizes available in the database were used in the analysis.
Data collection	Surveys available in the TreeCo database were filtered according to do the criteria described above. All meta-data associated with each survey were compiled from the literature available online and validated by the authors RAFL, GRP, ALG and ACV. The same was true for species trait information.
Timing and spatial scale	The list of surveys used in the analysis covers the entire range of the Atlantic Forest (4–34o S latitude, 35–57o W longitude), which represents an area of about 136 million hectares in eastern and southern Brazil, eastern Paraguay and northeastern Argentina. Surveys used in this study were mainly conducted between 1995 and 2017 (95% of the total of surveys), with the oldest survey being conducted in 1982.
Data exclusions	The TreeCo database has more surveys than the ones used in this study. To obtain the final list of surveys used during analysis, the database was filtered to get only the surveys conducted in natural Atlantic Forests (TreeCo database has a pre-established field for planted and natural forests), including trees with diameter at breast height (dbh) ≥3, ≥5 and ≥10 cm and using plots or point-centered quarters sampling methods. Surveys conducted in dry deciduous forests and early secondary forests, locally known as 'capoeiras' and 'capoeirões', were also excluded as these forests function differently from moist and rain forest remnants.
Reproducibility	N/A (this study does not involve experiments)
Randomization	No randomized block design was conducted, although the eco-regions of the Atlantic Forest were used as a random effect within the mixed-effect regression modeling approach.
Blinding	N/A (this study does not involve blinding)

Did the study involve field work? ☐ Yes ☒ No

Reporting for specific materials, systems and methods

We require information from authors about some types of materials, experimental systems and methods used in many studies. Here, indicate whether each material, system or method listed is relevant to your study. If you are not sure if a list item applies to your research, read the appropriate section before selecting a response.

Materials & experimental systems

n/a	Involved in the study
☒	☐ Antibodies
☒	☐ Eukaryotic cell lines
☒	☐ Palaeontology
☒	☐ Animals and other organisms
☒	☐ Human research participants
☒	☐ Clinical data

Methods

n/a	Involved in the study
☒	☐ ChIP-seq
☒	☐ Flow cytometry
☒	☐ MRI-based neuroimaging
